# An ETFDH-driven metabolon supports OXPHOS efficiency in skeletal muscle by regulating coenzyme Q homeostasis

**DOI:** 10.1038/s42255-023-00956-y

**Published:** 2024-01-19

**Authors:** Juan Cruz Herrero Martín, Beñat Salegi Ansa, Gerardo Álvarez-Rivera, Sonia Domínguez-Zorita, Pilar Rodríguez-Pombo, Belén Pérez, Enrique Calvo, Alberto Paradela, David G. Miguez, Alejandro Cifuentes, José M. Cuezva, Laura Formentini

**Affiliations:** 1 Departamento de Biología Molecular, Centro de Biología Molecular Severo Ochoa (CBMSO, UAM–CSIC), Universidad Autónoma de Madrid (UAM), Madrid, Spain; 2 Laboratorio Foodomics, Instituto de Investigación en Ciencias de la Alimentación (CIAL), Consejo Superior de Investigaciones Científicas (CSIC)–Universidad Autónoma de Madrid (UAM), Madrid, Spain; 3 Centro de Investigación Biomédica en Red de Enfermedades Raras (CIBERER), ISCIII, Madrid, Spain; 4 Instituto de Investigación Hospital 12 de Octubre (i+12), Madrid, Spain; 5 Instituto Universitario de Biología Molecular (IUBM), Universidad Autónoma de Madrid (UAM), Madrid, Spain; 6 Centro de Diagnóstico de Enfermedades Moleculares (CEDEM), Universidad Autónoma de Madrid (UAM), Madrid, Spain; 7 Instituto de Investigación Universitaria La Paz (IDIPAZ), Madrid, Spain; 8 Proteomics Unit, Centro Nacional de Investigaciones Cardiovasculares (CNIC) Carlos III, Madrid, Spain; 9 Proteomics Unit, Centro Nacional de Biotecnología (CNB)–Consejo Superior de Investigaciones Científicas (CSIC), Madrid, Spain; 10 Departamento de Física de la Materia Condensada, IFIMAC, Universidad Autónoma de Madrid (UAM), Madrid, Spain

**Keywords:** Mechanisms of disease, Molecular medicine, Organelles, Enzyme mechanisms

## Abstract

Coenzyme Q (Q) is a key lipid electron transporter, but several aspects of its biosynthesis and redox homeostasis remain undefined. Various flavoproteins reduce ubiquinone (oxidized form of Q) to ubiquinol (QH_2_); however, in eukaryotes, only oxidative phosphorylation (OXPHOS) complex III (CIII) oxidizes QH_2_ to Q. The mechanism of action of CIII is still debated. Herein, we show that the Q reductase electron-transfer flavoprotein dehydrogenase (ETFDH) is essential for CIII activity in skeletal muscle. We identify a complex (comprising ETFDH, CIII and the Q-biosynthesis regulator COQ2) that directs electrons from lipid substrates to the respiratory chain, thereby reducing electron leaks and reactive oxygen species production. This metabolon maintains total Q levels, minimizes QH_2_-reductive stress and improves OXPHOS efficiency. Muscle-specific *Etfdh*^−/−^ mice develop myopathy due to CIII dysfunction, indicating that ETFDH is a required OXPHOS component and a potential therapeutic target for mitochondrial redox medicine.

## Abstract (web summary)

Electron-transfer flavoprotein dehydrogenase (ETFDH) is shown to associate with mitochondrial complex III (CIII) physically and functionally, thereby promoting electron channelling to increase CIII efficiency.

## Main

During oxidative phosphorylation (OXPHOS), the respiratory chain (electron transport chain (ETC)) transports electrons from whole-cell substrate oxidation to oxygen, allowing proton (H^+^) translocation outside the inner mitochondrial membrane (IMM) and storing energy in the mitochondrial membrane potential (ΔΨm). ΔΨm is coupled with ATP production, protein import to mitochondria, reactive oxygen species (ROS) production and calcium signalling^1^. As a result, OXPHOS ultimately regulates intracellular events such as cell death, immunity, organelle communication, thermogenesis and metabolism^2^.

All electrons enter the ETC through either integral (complex I (CI) and CII) or peripheral (non-proton-pumping dehydrogenases) IMM iron–sulfur flavoproteins^3^ that retrieve the energy provided by reducing equivalents (reduced forms of NADH and FADH_2_) and transfer electrons to the mobile carrier coenzyme Q (Q, also called ubiquinone in its oxidized form and ubiquinol (QH_2_) in its reduced form). QH_2_ is then oxidized to Q by CIII in the so-called Q cycle^4,5^. This is a two-step reaction involving the formation of a semiquinone radical (QH•) that is stored in the haem group of the CIII subunit cytochrome (Cyt) *b* while waiting for another QH_2_ molecule to complete the cycle^5^. Due to the high QH• instability and standby time, the reaction has been widely debated and has undergone modifications over the years^5^. Intriguingly, the original Q cycle proposed by Peter D. Mitchell involved an IMM flavoprotein that assists CIII in oxidizing QH_2_ (refs. ^6,7^). Although it has been elegantly demonstrated in in vitro chromatophores that CII does not participate in the Q cycle^5,8^ and CIII acts with a self-contained mechanism^9,10^, the involvement of other Q reductases in assisting CIII’s catalytic activity in higher-complexity models such as mitochondria has not been evaluated. Among these flavoproteins, which integrate OXPHOS with pyrimidine synthesis and sulfur, lipid and amino acid metabolism^3^, electron-transfer flavoprotein dehydrogenase (ETFDH) links free fatty acid (FFA) β-oxidation (FAO), choline metabolism and branched-chain amino acid (BCAA) catabolism to oxygen respiration. ETFDH is a 64-kDa IMM protein monomer containing one 4Fe–4S cluster, one FAD molecule and one Q-binding site^11^. As FAO’s final electron acceptor, ETFDH receives electrons from electron-transfer flavoprotein (ETF) and reduces Q to QH_2_ (ref. ^11^). Pathological mutations in *ETFDH* lead to multiple acyl-coenzyme A (CoA) dehydrogenase deficiency (MADD; Online Mendelian Inheritance in Man:231680, ORPHA:394532), a rare autosomal recessively inherited disorder of FFA and BCAA metabolism^12,13^. MADD has heterogeneous clinical manifestations, including encephalomyopathy with interfibre lipid droplets, hypoglycaemia and metabolic aciduria with blood accumulation of the FAO intermediate glutaric acid^14,15^. Currently, there is no cure for MADD. Treatments are limited to low-fat, low-protein, high-carbohydrate diets supplemented with Q or riboflavin and fasting avoidance^12,16^. Interestingly, MADD has been associated with ETC dysfunctions^17^ and secondary Q deficiency, suggesting that ETFDH participates in OXPHOS and Q homeostasis^4,16–18^. In this regard, a substrate-mediated Q compartmentalization^19–21^ has been hypothesized: when FFAs or BCAAs are used as energy sources, electrons may be directed towards a flavoprotein-dependent Q pool^22,23^. Consistent with this, a direct interaction between ETC and FAO proteins has been proposed^24–26^. This raises the possibility that, in skeletal muscle (Skm), the major site of FAO and BCAA catabolism, ETFDH may regulate the biosynthesis of the FAD-dependent Q pool, which might also explain the Q deficiency associated with MADD. However, the role of ETFDH in Q homeostasis remains unclear due to a lack of viable in vivo models, as total-body ETFDH deletion is incompatible with life.

Herein, using both in vitro and in vivo Skm-specific ETFDH-knockout (ETFDH-ko) models, we demonstrate that ETFDH is essential for OXPHOS efficiency by participating in the Q cycle and biosynthesis. Following depletion of Skm ETFDH, the Cyt *b* subunit of CIII becomes constitutively reduced and inhibited, leading to pathological QH_2_ accumulation and reductive stress, which ultimately causes aberrant myoblast cell cycle and myogenesis. Accordingly, the conditional and Skm-specific *Etfdh*^−/−^ mouse presents myopathy due to CIII dysfunction. Replacing ETFDH with a mutant version lacking a functional Q-binding site mimicked this redox imbalance. Introducing alternative oxidase (AOX)^23^ reduced ROS levels but did not restore ΔΨm and CIII activity, indicating that a functional ETFDH is required for OXPHOS.

We have also identified a metabolon (comprising ETFDH, CIII and the Q-biosynthesis regulator COQ2) that maintains Q homeostasis. Consistent with this, limiting the amount of QH_2_ in mouse myocytes and patient-derived fibroblasts minimized reductive stress, suggesting that MADD-associated Q deficiency is not a comorbidity but a cellular strategy to compensate for ETFDH dysfunction.

**Fig. 1:**
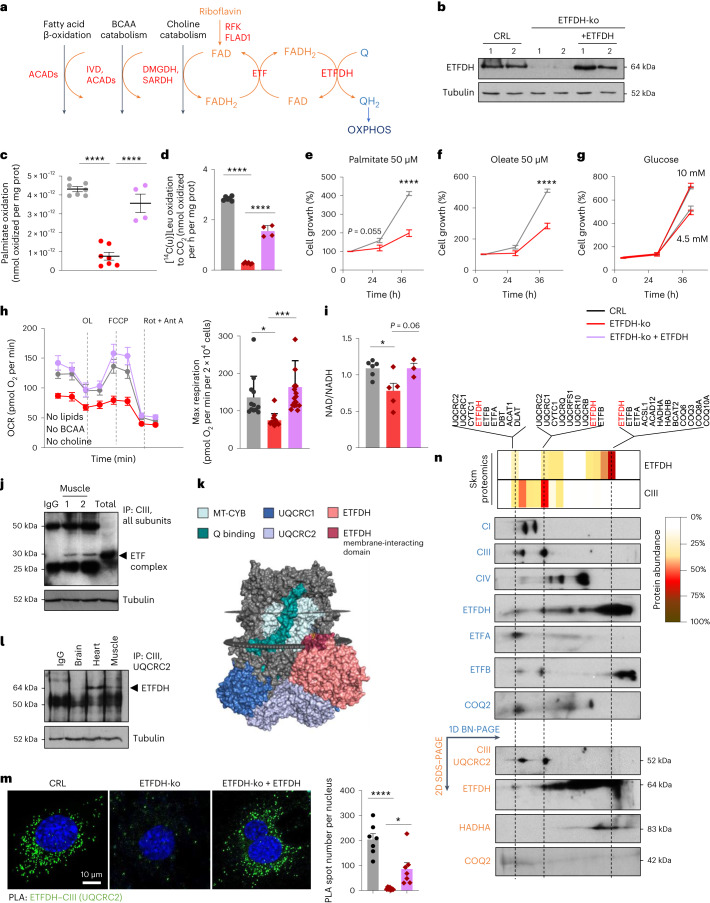
ETFDH–CIII interaction is required for OXPHOS efficiency. **a**, Scheme depicting how the iron–sulfur flavoprotein ETFDH canalizes electrons from FAO, BCAA catabolism and choline metabolism to OXPHOS. The FAD (orange) and Q (blue) redox reactions are illustrated, with the proteins of the ETFDH pathway highlighted in red. ACADs, acyl-CoA dehydrogenases; IVD, isovaleryl-CoA dehydrogenase; DMGDH, dimethylglycine dehydrogenase; SARDH, sarcosine dehydrogenase; RFK, riboflavin kinase; FLAD1, FAD synthetase 1. **b**, Representative western blot of ETFDH protein levels in CRL, ETFDH-ko and cDNA-rescued ETFDH-ko (+ETFDH) myoblasts. Two samples per condition. Tubulin is shown as a loading control. **c**, FAO fluxes in CRL, ETFDH-ko and cDNA-rescued ETFDH-ko myoblasts. *n* = 3. **d**, [^14^C(u)]leucine (Leu) oxidation to CO_2_ in myoblasts. *n* = 4 replicates per condition. **e**–**g**, Cell growth in CRL and ETFDH-ko myoblasts cultured in substrate-free medium supplemented with palmitate (**e**), oleate (**f**) or glucose (**g**). *n* = 3, seven replicates per condition. **h**, Left, representative respiratory profile of CRL (grey trace), ETFDH-ko (red trace) and cDNA-rescued ETFDH-ko (lavender trace) myoblasts. Right, quantification histogram. *n* = 3. **i**, NAD/NADH levels in CRL, ETFDH-ko and cDNA-rescued ETFDH-ko myoblasts. *n* = 3. **j**, Immunocapture (IP) of all subunits of CIII blotted with anti-ETF antibody (ETFA) in Skm from mouse hindlimb. Tubulin is shown as a loading control. IgG, immunoglobulin G. **k**, PyMOL representation of a ClusPro docking study for CIII–ETFDH interaction. **l**, Immunocapture (IP) of UQCRC2 blotted with anti-ETFDH antibody in mouse brain, heart and Skm extracts. Tubulin is shown as a loading control. **m**, Left, representative images of PLA between CIII (UQCRC2) and ETFDH in CRL, ETFDH-ko and cDNA-rescued ETFDH-ko myoblasts. Right, quantification histogram. Seven images per condition, *n* = 3. **n**, Representative 1D BN-PAGE and 2D SDS–PAGE of mitochondrial membrane proteins from wt mice, followed by proteomics. Migration in 1D BN-PAGE of respiratory CI, CIII and CIV; ETF complex subunits ETFDH, ETFA and ETFB; and COQ2 (in blue) are shown. Migration in 2D SDS–PAGE of CIII, ETFDH, the FAO enzyme HADHA, and COQ2 (in orange) are shown. The upper image shows the proteomic analysis of 12 bands from 1D BN-PAGE. White to red scale indicates protein abundance within a band. ETFDH was found to comigrate with CIII in two bands. Results are shown as the mean ± s.e.m. of the indicated *n*. *, **, ***, ****P < 0.05, 0.01, 0.001 and 0.0001 when compared with CRL by one-way ANOVA with Tukey’s test (**c**, **d**, **h**, **i**, **m**) or two-way ANOVA with Šidák’s test (**e**, **f**, **g**). Source data

**Fig. 2:**
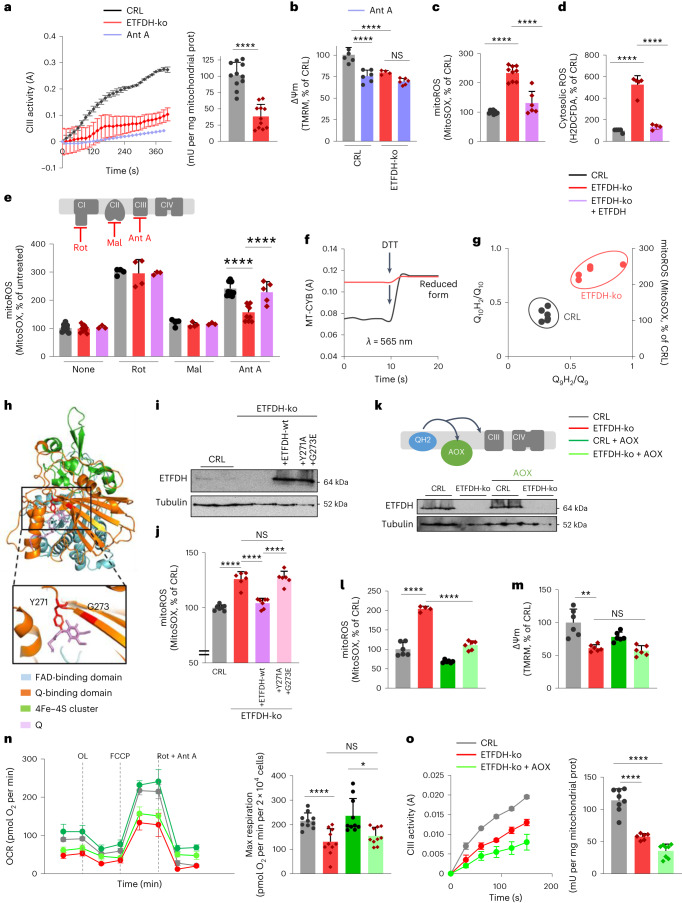
ETFDH participates in the Q cycle. **a**, CIII enzymatic activity in isolated mitochondria from CRL and ETFDH-ko myocytes. The left panel shows absorbance (A) changes over time. In the right panel, CIII activity is presented as mU per mg of mitochondrial protein. *n* = 3. Ant A was used as a CIII inhibitor. **b**, ΔΨm in CRL and ETFDH-ko myocytes in the presence or absence of 1 µM Ant A. *n* = 3. **c**–**e**, Mitochondrial (**c**, **e**) and cytosolic (**d**) ROS in CRL, ETFDH-ko and cDNA-rescued ETFDH-ko (+ETFDH) myoblasts in the presence or absence of Rot, Mal and Ant A. The upper scheme in **e** illustrates the site of action of each inhibitor. *n* = 3. mitoROS, mitochondrial ROS. **f**, Absorbance of Cyt *b* (*λ* = 565 ± 5 nm) before and after DTT administration in isolated mitochondria of CRL (black trace) and ETFDH-ko (red trace) myocytes. *n* = 3. **g**, Relation between Q_10_H_2_/Q_10_ ratio, Q_9_H_2_/Q_9_ ratio and mitochondrial ROS in CRL and ETFDH-ko myoblasts. *n* = 3. **h**, ETFDH structure highlighting two highly conserved amino acids in the Q-binding domain: Y271 and G273. **i**,**j**, Representative western blot analysis of ETFDH protein levels (**i**) and mitochondrial ROS (**j**) in CRL and ETFDH-ko myoblasts expressing native (ETFDH-wt) or mutated (ETFDH-Y271A,G273E) ETFDH protein. *n* = 3. **k**, Schematic of AOX functioning (top) and representative western blot of ETFDH expression (bottom) in CRL and ETFDH-ko cells expressing or not expressing AOX. **l**–**o**, Mitochondrial ROS (**l**), ΔΨm (**m**), respiratory profile (**n**) and CIII activity (**o**) in CRL and ETFDH-ko cells expressing or not expressing AOX. *n* = 3. Results are shown as the mean ± s.e.m. of the indicated *n*. NS, not significant. *, **, ***, ****P < 0.05, 0.01, 0.001 and 0.0001 when compared with CRL by two-tailed Student’s *t* test (**a**), one-way ANOVA with Tukey’s test (**c**, **d**, **j**, **l**–**o**), and two-way ANOVA with Šidák’s test (**b**) or Tukey’s test (**e**). Source data

**Extended Data Fig. 1:**
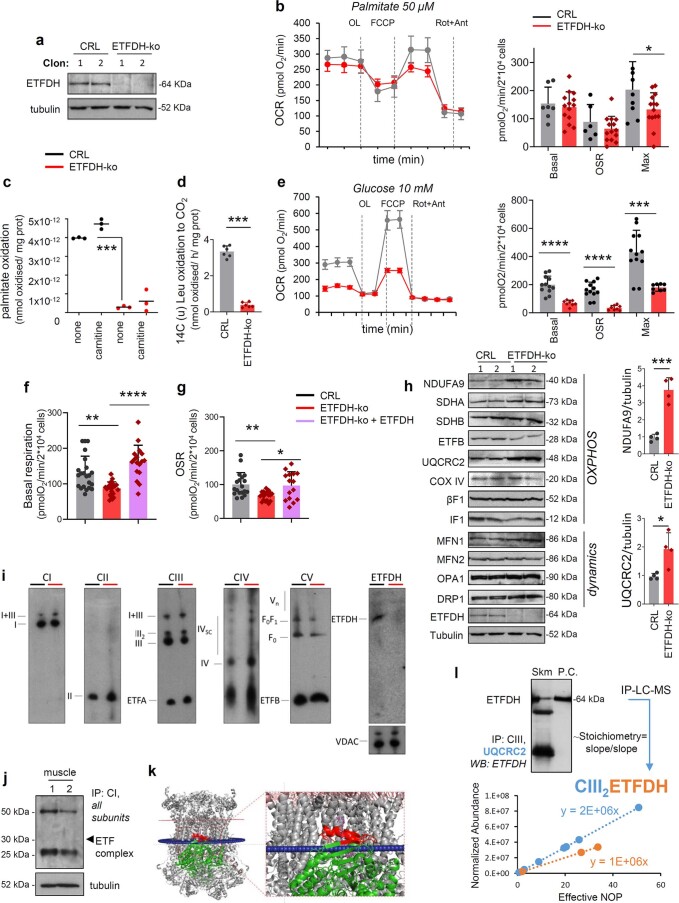
The absence of ETFDH impairs oxygen consumption. **a**) Representative western blot of ETFDH in 2 clones of control (CRL) and ETFDH-ko myocytes. Tubulin as loading control. **b, e**) Representative respiratory profile using palmitate (**b**) or glucose (**e**) as a substrate. OCR, oxygen consumption rate; OL, oligomycin; Rot, rotenone; Ant, antimycin A. Analysis in the right histograms. n = 3. **c**) FFA β-oxidation fluxes in control (CRL) and ETFDH-ko myocytes in the presence or absence of 1 mM carnitine. n = 3. **d**) 14 C(u)-leucine oxidation to CO_2_ in myocytes expressing or not ETFDH. n = 3 experiments, 6 replicates/condition. **f, g**) Basal and oligomycin-sensitive respiration in control (CRL), ETFDH-ko and cDNA-rescued ETFDH-ko (+ETFDH) myoblasts, corresponding to Fig. 1h. **h**) Representative western blot expression of proteins related to mitochondrial OXPHOS and dynamics. Subunits NDUFA9 (CI), SDHA and SDHB (CII), ETFB and ETFDH (ETF complex), UQCRC2 (CIII), COX IV (CIV), β-F1 ATPase and IF1 (CV) are shown. Mitofusins I and II (MFN1), OPA1-Mitochondrial Dynamin Like GTPase (OPA1) and Dynamin 1 Like (DRP1) are also shown. Two samples per condition. Tubulin as loading controls. 6 replicates, n = 3. **i**) Representative BN^−^PAGE of mitochondria from CRL and ETFDH-ko myocytes. The migration of the respiratory complexes/supercomplexes CI-V is indicated. ETFDH was observed to comigrate with CI + CIII. VDAC is shown as a loading control. **j**) Immunocapture (IP) of all subunits of CI blotted with anti-ETF antibody (ETFA) in Skm from mouse hindlimb. Tubulin as loading control. **k**) Pymol representation of ClusPro docking study for CIII-ETFDH interaction. **l**) Immunocapture (IP) of UQCRC2 blotted with anti-ETFDH antibody in Skm extracts from mouse hindlimb. In the lower panel, the graph shows the number of tryptic peptides (effective NOP) plotted against the proteomic normalized abundance of each protein in the CIII and ETF complexes within the IP sample. The stoichiometry of ETFDH-CIII complexes is estimated as the ratio of the slopes. Results are shown as the mean ± SEM of the indicated n. *, **, ***, **** p < 0.05; 0.01, 0.001 and 0.0001 when compared to CRL by two-tailed Student’s t-test (b, d, e), one-way ANOVA with Tukey’s test (f, g), and two-way ANOVA with Sidak’s test (c). Source data

To understand the impact of limiting electron transfer (Fig. 1a) on OXPHOS efficiency and Skm homeostasis, we generated, by clustered regularly interspaced short palindromic repeats (CRISPR)–Cas9 technology, different clones of mouse myoblasts lacking ETFDH (Fig. 1b and Extended Data Fig. 1a). To discard off-target effects, results are shown including cDNA-rescue experiments (Figs. 1 and 2). Both ETFDH-ko myoblasts (Fig. 1c,d) and derived myocytes (Extended Data Fig. 1b–d) had impaired FAO rates (Fig. 1c and Extended Data Fig. 1b,c) and BCAA catabolism (Fig. 1d and Extended Data Fig. 1d). As a result, cells failed to proliferate when palmitate (Fig. 1e) or oleate (Fig. 1f) were used as nutrients. On the contrary, when myoblasts relied on glucose and electron flow bypassed ETFDH, cell growth was similar to control (CRL) regardless of glucose concentrations (Fig. 1g). Under similar conditions, with glucose as the primary ATP source and electrons mainly entering through CI (and to a lesser extent through CII and glycerol-3-phosphate dehydrogenase (GPD2)), oxygen consumption rates (OCRs) were expected to be comparable with or without ETFDH. However, ETFDH-ko cells exhibited lower basal and maximal respiration (Extended Data Fig. 1e). To rule out the possibility that this was due to residual ETFDH-mediated catabolism, we repeated the experiment in FFA-, BCAA- and choline-free medium (Fig. 1h and Extended Data Fig. 1f,g). Interestingly, when glucose was the sole energy substrate, the ETFDH-dependent decrease in maximal respiration (Fig. 1h) and in the NAD/NADH ratio (Fig. 1i) suggested an unexpected role of ETFDH in maintaining ETC efficiency.

**Extended Data Fig. 2:**
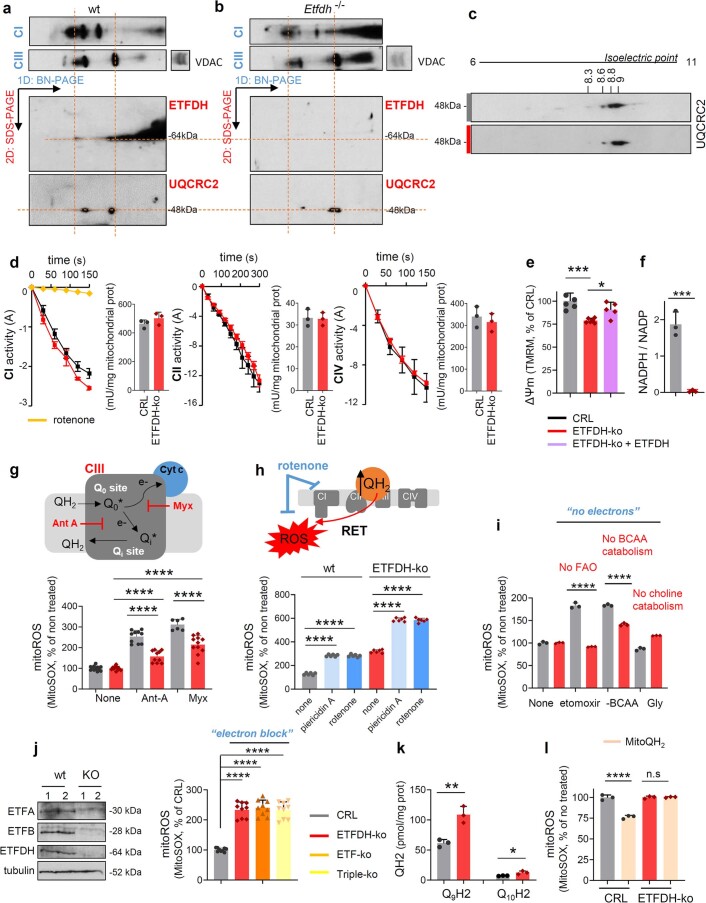
Disruption of the ETFDH-CIII metabolon leads to ROS and QH2 accumulation. **a, b**) Representative 1D-BN^−^PAGE/2D-SDS-PAGE of mitochondrial membrane proteins from wt and *Etfdh*^-/-^ mice. Migration in 1D-BN-PAGE of respiratory CI, CIII (in blue) and in 2D-SDS-PAGE of CIII and ETFDH (in red) are shown. ETFDH was found to comigrate with CIII. **c**) 2D-PAGE IEF gels of CRL and ETFDH-ko myocyte extracts. The isoelectric point (pI) of the CIII subunit UQCRC2 was calculated by protein migration in pH 3–10 NL strips. **d**) CI, CII and CIV enzymatic activity in isolated mitochondria from control (CRL) and ETFDH-ko myocytes. n = 3. Rotenone, carboxin and CN^-^ were used as control for calculating specific CI, CII and CIV activity, respectively. **e**) Mitochondrial membrane potential ΔΨm in control (CRL), ETFDH-ko and cDNA-rescued ETFDH-ko (+ETFDH) myoblasts. n = 3. **f**) NADPH/NADP levels in control (CRL) and ETFDH-ko myoblasts. n = 3. **g–i**) Mitochondrial ROS in control (CRL) and ETFDH-ko myocytes, in the presence or absence of CIII inhibitors antimycin A (Ant-A) and myxothiazole (Myx) (**g**) or CI inhibitors piericidin A and rotenone (**h**). The upper scheme in **g** illustrates the site of action of each inhibitor; the one in **h** the RET mechanism of action. In **i**, myocytes were treated with etomoxir (no FAO), glycine (no choline catabolism) or grown in no-BCAA media (no BCAA catabolism). n = 3. **j**) Left panel. Representative western blot of proteins related to ETF complex, ETFA, ETFB, ETFDH in 2 clones of control (CRL) and triple ETFA-ko, ETFB-ko, ETFDH-ko myoblasts. Tubulin as loading control. Right panel. Mitochondrial ROS in control (CRL) and ETFDH-ko, ETFA-ko and triple ETFA-ko, ETFB-ko, ETFDH-ko myocytes. n = 3. **k**) Q_9_H_2_ and Q_10_H_2_ levels in control (CRL) and ETFDH-ko myoblasts. n = 3. **l**) Mitochondrial ROS in control (CRL) and ETFDH-ko cells treated or not with the reduced form of MitoQ (MitoQH_2_, 20 nM). n = 3. Results are shown as the mean ± SEM of the indicated n. *, **, ***, **** p < 0.05; 0.01, 0.001 and 0.0001 when compared to CRL or wt by two-tailed Student’s t-test (d, f, k), one-way ANOVA with Tukey’s test (e, j) or with Sidak’s test (l), and two-way ANOVA with Sidak’s test (g-i). Source data

To unveil ETFDH-mediated ETC dysfunction, we analysed the expression and assembly of OXPHOS complexes in myocytes (Extended Data Fig. 1h,i). The absence of ETFDH slightly upregulated the CI subunit NDUFA9 and the CIII subunit QH_2_–Cyt *c* reductase core protein 2 (UQCRC2; Extended Data Fig. 1h), which did not explain the observed OCR reduction. No significant changes were observed in the expression, assembly and superassembly of other complexes or in proteins related to mitochondrial dynamics (Extended Data Fig. 1h,i). Intriguingly, on blue native (BN) gels, the ETFDH migration pattern resembled that of the supercomplex CI + CIII, suggesting a potential association (Extended Data Fig. 1i). Indeed, a multifunctional complex formed by the ETC and FAO machinery, aimed at conferring kinetic advantages^26^, has been previously hypothesized^24^. Notably, the ETF complex coimmunoprecipitated with CIII subunits in Skm mitochondria isolated from mouse hindlimbs (Fig. 1j) but not with CI (Extended Data Fig. 1j). This result is consistent with the proposed ETC superstructure reorganization in response to FAO, which leads to CIII release from CI + CIII supercomplexes to receive FADH_2_-derived electrons^23^. ClusPro docking analysis proposed a conformational arrangement for ETFDH–CIII interaction in which the ETFDH hydrophobic Q-binding domain, anchored to the IMM, directly bound CIII at the IMM–matrix interface, involving the CIII subunits UQCRC1 and UQCRC2 (Fig. 1k and Extended Data Fig. 1k) and thus facilitating QH_2_ transfer. The coimmunoprecipitation (co-IP) of UQCRC2–ETFDH (Fig. 1l) and the proximity ligation assay (PLA; Fig. 1m and Supplementary Fig. 1) confirmed their interaction, with proteomic analysis of the IP revealing an approximate 1:2 stoichiometry of ETF–CIII complexes (Extended Data Fig. 1l). As additional evidence, ETFDH and UQCRC2 comigrated in first-dimension (1D) BN-PAGE and second-dimension (2D) SDS–PAGE immunoblots of Skm mitochondrial membrane proteins (Fig. 1n and Extended Data Fig. 2a,b). We next divided the BN gel into 12 bands and performed proteomics on each. CIII subunits and the ETF complex subunits (ETFDH, ETFA and ETFB) were codetected in two bands, corresponding to similar BN-PAGE migration patterns (Fig. 1n). Notably, both ETFDH and CIII also exist as free monomers and dimers without binding to each other (Fig. 1n). Further studies are needed to unveil specific conditions for increasing or limiting CIII–ETF interaction. Importantly, ETFDH–UQCRC2 binding did not alter the isoelectric point of the CIII subunit (Extended Data Fig. 2c), indicating no ETFDH-mediated post-translational modifications of UQCRC2 affecting its protein charge.

To determine whether the lack of CIII–ETF interaction affects OXPHOS, we assessed the enzymatic activity of the four ETC complexes in isolated mitochondria from CRL and ETFDH-ko myocytes (Fig. 2a and Extended Data Fig. 2d). Consistent with a specific binding to CIII, no changes were observed in CI, CII and CIV activities (Extended Data Fig. 2d). However, myocyte-derived ETFDH-ko mitochondria showed a 60% decrease in the CIII-mediated reduction of Cyt *c* (Fig. 2a). This CIII dysfunction led to inefficient H^+^ translocation through the IMM, resulting in decreased ΔΨm (Fig. 2b and Extended Data Fig. 2e). Remarkably, the CIII inhibitor antimycin A (Ant A) had the same impact as the absence of ETFDH in reducing ΔΨm in CRL myocytes (Fig. 2b). In contrast, it showed no effect on ETFDH-ko cells (Fig. 2b), confirming that CIII was already inhibited in this condition.

CIII dysfunctions are linked to an increase in ROS^27–30^. Accordingly, ETFDH-ko myoblasts produced higher amounts of mitochondrial (superoxide; Fig. 2c) and cytosolic (H_2_O_2_; Fig. 2d) ROS compared with CRL. This was also accompanied by a decrease in the NADPH/NADP ratio (Extended Data Fig. 2f). Interestingly, while rotenone (Rot) and malonate (Mal), inhibitors of CI and CII, respectively^31^, induced the same spike in ROS in CRL and ETFDH-ko cells, Ant A (Fig. 2e) and myxothiazole (Extended Data Fig. 2g) generated a significantly lower ROS increase in the absence of ETFDH. This further confirms that CIII was already inhibited in ETFDH-ko cells.

Changes in FAD-derived electron flux have been suggested to trigger reverse electron transport (RET), resulting in Rot-sensitive superoxide production^32,33^ and CI oxidation and degradation^23^. Surprisingly, (1) no CI degradation was observed (Extended Data Fig. 1i) and (2) neither Rot nor piericidin A (inhibitors of CI) reduced the ROS burst in ETFDH-ko cells (Extended Data Fig. 2h), suggesting no RET in our model. Genetic FAO defects are known to increase ROS^34^. However, limiting ETFDH pathway use by substrate deprivation—inhibiting FAO with etomoxir, omitting BCAA from the medium or hindering choline metabolism by glycine (‘no electron’ situation)—induced a substantial increase in mitochondrial ROS only in CRL and not in ETFDH-ko myocytes (Extended Data Fig. 2i). On the contrary, when FFA, BCAA and choline were present in the medium, forcing pathway use, the deletion of any protein of the ETF complex (ETFA, ETFB or ETFDH, or all of them; Extended Data Fig. 2j) led to a similar increase in ROS levels (Extended Data Fig. 2j; ‘electron block’ situation). These results are in line with clinical data on FAO dysfunctions^34^ and suggest that maintaining a homoeostatic electron flux through the ETFDH pathway is essential for preventing harmful CIII-mediated ROS production, underlining ETFDH as a necessary OXPHOS component.

This also raised the possibility that ETFDH may participate in CIII’s mechanism of action—the Q cycle—in which two QH_2_ are oxidized while one Q is reduced^5^. In line with this hypothesis, following the deletion of ETFDH, the radical semiquinone should be trapped in the haem group of the catalytic subunit of CIII, mitochondrial Cyt *b* (MT-CYB), making it unable to accept electrons. Consistent with this, MT-CYB was constitutively reduced in ETFDH-ko myocyte mitochondria (Fig. 2f), as in the presence of Ant A^35^. As a further demonstration, we measured the amounts of reduced and oxidized forms of Q_10_ and Q_9_ in myoblasts (Extended Data Fig. 2k), which are the most common forms of Q in humans and rodents, respectively^20^. Surprisingly, the deletion of a Q reductase did not prompt the expected accumulation of its substrate, oxidized Q, but led to increased Q_10_H_2_/Q_10_ and Q_9_H_2_/Q_9_ ratios (Fig. 2g and Extended Data Fig. 2k), as observed by inhibiting CIII with myxothiazol^36^. In line with a pathological increase in endogenous QH_2_ levels (Extended Data Fig. 2k), the reduced form of the mitochondrial scavenger MitoQ (MitoQH_2_) decreased superoxide production in CRL but not in ETFDH-ko cells (Extended Data Fig. 2l), suggesting that QH_2_ accumulation following ETFDH deletion already caused reductive stress.

**Extended Data Fig. 3:**
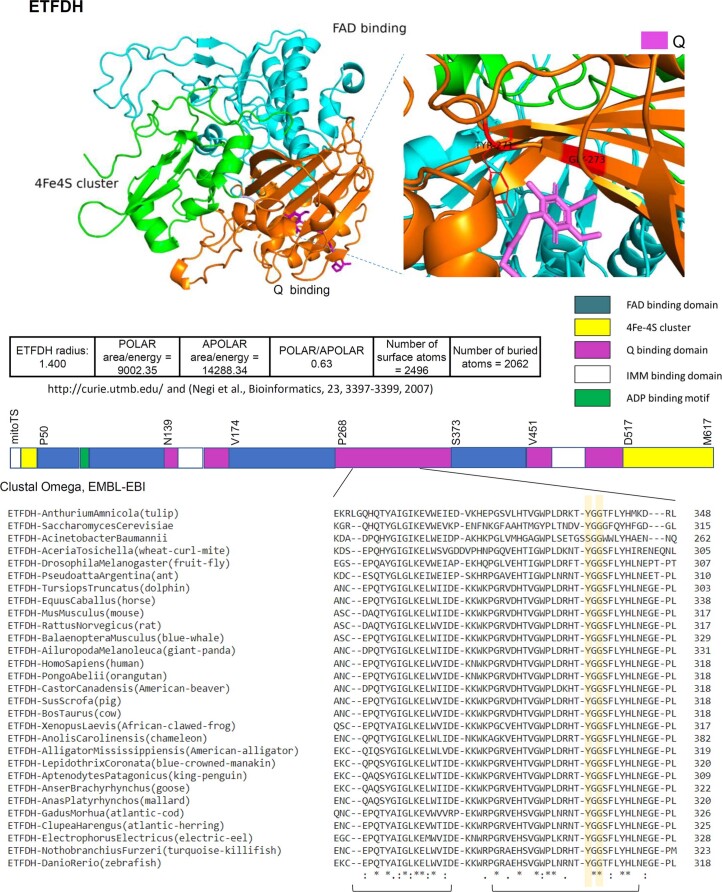
ETFDH 3D-structure and domains. Fe/S cluster, FAD-binding and Q-binding domains are shown in different colours. ETFDH’s biophysical parameters are also shown (InterProSurf, http://curie.utmb.edu/; see (Negi et al., Bioinformatics, 23, 3397-3399, 2007). In the lower panel, ETFDH sequence alignment across species (Clustal Omega, EMBL-EBI). Note that Y271 and G273 (in yellow) are conserved across almost all species analysed.

**Extended Data Fig. 4:**
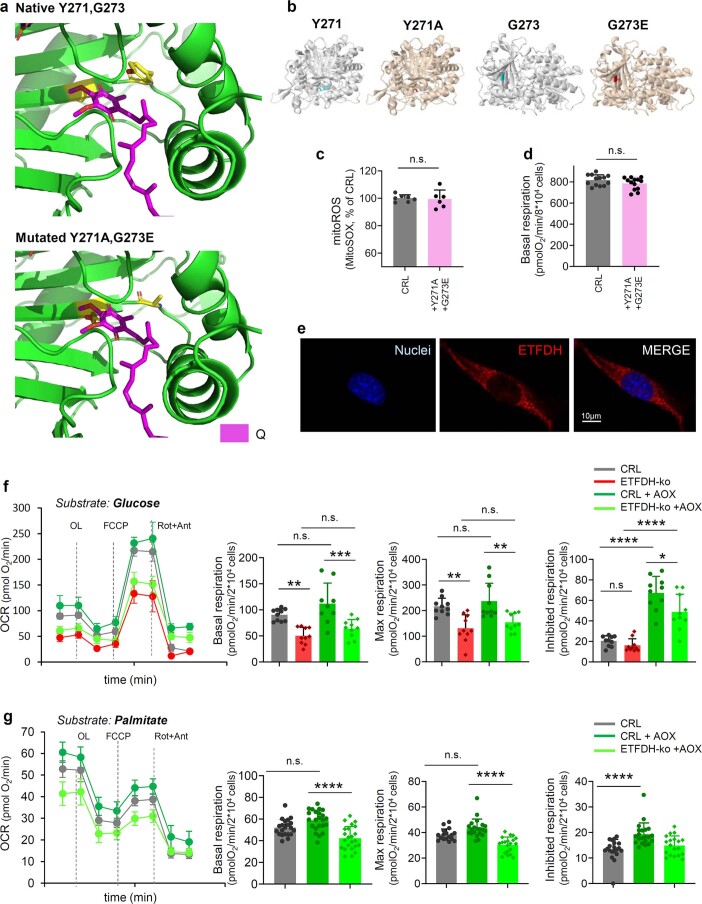
An efficient ETFDH protein is essential for optimal CIII and OXPHOS activities. **a**) Pymol representation of Q-binding site in ETFDH_wt_ and ETFDH_Y271A,G273E_ proteins. **b**) Missense-3D structure of native (Y271, G273) and mutated (Y271A and G273E) ETFDH. UniProt ID: Q16134. PDB code: 2gmh. PDB chain ID: A. The Y304A substitution (Y271A in PDB) does not trigger MolProbity clash alert. The local clash score for wild type is 47.24 and the local clash score for mutant is 45.34. This substitution does not alter the secondary structure, nor trigger disallowed phi/psi alert. However, the Y271A substitution leads to the expansion of Q-binding cavity volume by 133.488 Å^3. The G306E substitution (G273E in PDB) does not trigger MolProbity clash alert. The local clash score for wild type is 46.92 and the local clash score for mutant is 54.06. This substitution does not alter the secondary structure (extended strand in parallel and/or anti-parallel β-sheet conformation), nor trigger disallowed phi/psi alert. However, the G273E substitution leads to the contraction of Q-binding cavity volume by 58.968 Å^3. **c, d**) Mitochondrial ROS (**c**) and basal respiration (**d**) in control (CRL) and ETFDH_Y271A,G273E_ CRL myoblasts. n = 3. **e**) Immunofluorescence from ETFDH-ko myoblasts expressing ETFDH_Y271A,G273E_. Red, ETFDH; blue, nuclei: DAPI; Images are representative of 10 pictures/condition. **f, g**) Representative respiratory profile of control (CRL, gray trace), ETFDH-ko (red traces), wt expressing AOX (dark green traces) and ETFDH-ko expressing AOX (light green traces) cells, using glucose (**f**) or palmitate (**g**) as a substrate. OCR, oxygen consumption rate; OL, oligomycin; FCCP, carbonyl cyanide 4-(trifluoromethoxy)phenylhydrazone; Rot, rotenone; Ant, antimycin A. Analysis in the right histograms. 10 and 24 replicates/condition, n = 3. Results are shown as the mean ± SEM of the indicated n. *, **, ***, **** p < 0.05; 0.01, 0.001 and 0.0001 when compared to CRL by two-tailed Student’s t-test (c, d), and one-way ANOVA with Tukey’s test (f, g). Source data

This also raised the possibility that a correct ETFDH-to-CIII electron transfer is needed to avoid reductive stress. In this regard, a region in the ETFDH sequence (Protein Data Bank (PDB) ID: 2GMH) is highly conserved and participates in Q binding^11^. Specifically, two amino acids from the Q-binding domain are conserved among almost all the species analysed (Extended Data Fig. 3) and are responsible for direct interaction with the Q benzoquinone ring^11^: Y271 and G273 (numeration in PDB, corresponding to Y304 and G306 in the UniProt FASTA sequence) (Fig. 2h and Extended Data Fig. 3). Bioinformatic three-dimensional analysis of the protein structure predicted that the double-substitution Y271A and G273E (Fig. 2h and Extended Data Fig. 4a) results in steric hindrance with a modification of the Q-binding cavity volume (Extended Data Fig. 4a,b), which, according to previous data^11^, impairs electron transfer. Overexpression of the ETFDH-Y271A,G273E variant in CRL myoblasts did not result in significant changes in OCR and ROS production (Extended Data Fig. 4c,d), indicating no dominant functional changes. However, overexpression of the ETFDH-Y271A,G273E variant in ETFDH-ko myoblasts (which generates a still folded mitochondrial protein recognizable by the ETFDH antibody; Extended Data Fig. 4e) was sufficient to reproduce the total deletion of ETFDH in terms of CIII-mediated superoxide production (Fig. 2i,j), pointing out perturbations in Q binding and electron flux within the ETFDH structure as responsible for CIII dysfunction.

Finally, overexpression of AOX (Fig. 2k), which oxidized mitochondrial QH_2_ to Q without pumping protons^23^, significantly reduced superoxide levels following ETFDH deletion (Fig. 2l). However, AOX expression failed to restore ΔΨm (Fig. 2m), maximal respiration in both glucose and palmitate (Fig. 2n and Extended Data Fig. 4f,g), and CIII activity (Fig. 2o) in ETFDH-ko cells. These data indicate that (1) ROS are not the cause of Skm CIII inhibition and (2) altering the QH_2_/Q ratio alone is insufficient for restoring ETC functionality, suggesting that a functional ETFDH is necessary for CIII activity.

**Extended Data Fig. 5:**
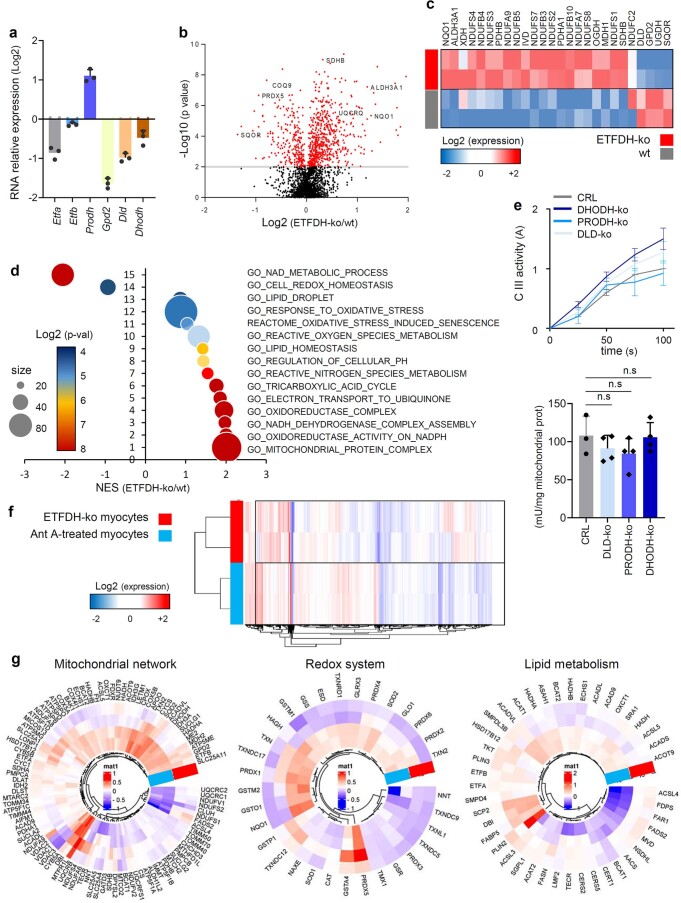
Quantitative proteomics in wt, Ant A-treated and ETFDH-ko myocytes. **a**) RNA relative expression of other IMM-flavoproteins in ETFDH-ko myoblasts compared to CRL. Results are shown as the mean ± SEM of 3 independent experiments. **b, c**) Quantitative proteomic analysis (TMT) of CRL and ETFDH-ko myocytes. Volcano plot (**b**) and heat-map (**c**) heat-map. A t-tests to compare groups in a pairwise fashion was used. A (-) log2 p-value > 4 was considered statistically significant. A 3-color palette was used: white, non-significance; progressively more saturated values of blue and red indicate increased significance. Blue and red also means downregulated or upregulated protein, respectively. **d**) Bioinformatic analysis of TMT proteomics included a dot chart displaying the Normalized Enrichment Score (NES) for 15 perturbed pathways, as determined through GSEA bioinformatics analysis. In this chart, dot color and size respectively denote NOM-p-values (nominal p-value) and the number of altered proteins within each pathway. As per the GSEA program output, for statistics, we used the NES value, representing the normalized enrichment score for the gene set across the analyzed gene sets, and the NOM p-value, which represents the statistical significance of the enrichment score as a measure of pathway enrichment. The nominal p-value is not adjusted for gene set size or multiple hypothesis testing; therefore, we introduced a third variable to account for the size (diameter of the circle) of the gene set. **e**) CIII enzymatic activity in isolated mitochondria from control (CRL) and other IMM-flavoprotein-ko myocytes (DLD, PRODH and DHODH). Antimycin A was used as control for calculating specific activity. Results are shown as the mean ± SEM of 3 independent experiments. **f**, **g**) Quantitative proteomic analysis (TMT) of ETFDH-ko and Antimycin A-treated CRL myocytes. Heat-map representation of overexpressed (red) and downregulated (blue) proteins. A (-) log2 p-value > 4 was considered statistically significant. Statistics compared to CRL by one-way ANOVA with Dunnett’s test (e). Source data

Notably, both the RNA (Extended Data Fig. 5a) and protein expression (Extended Data Fig. 5b,c) levels of other IMM Q reductases were decreased following ETFDH deletion, presumably in a futile attempt to counter reductive stress due to QH_2_ accumulation and to ameliorate the observed cellular distress (Extended Data Fig. 5d) by limiting further Q reduction. However, the specific myocyte deletion of proline dehydrogenase (PRODH), dihydroorotate dehydrogenase (DHODH) or dihydrolipoyl dehydrogenase (DLD) did not alter CIII activity (Extended Data Fig. 5e), indicating that ETFDH, but no other Q-reducing flavoprotein, is required for CIII and OXPHOS efficiency.

Accordingly, defects in CIII or ETFDH exhibit similarities in terms of perturbations in the myoblast proteome or patients’ urine and blood metabolome (Extended Data Fig. 5f,g and Supplementary Table 1). However, as expected, the impact of Ant A treatment (Extended Data Fig. 5f,g) or mutations affecting CIII (Supplementary Table 1) was observed to be more pronounced than that of ETFDH deletion, which resulted in only a ⁓50% inhibition of CIII activity.

**Fig. 3:**
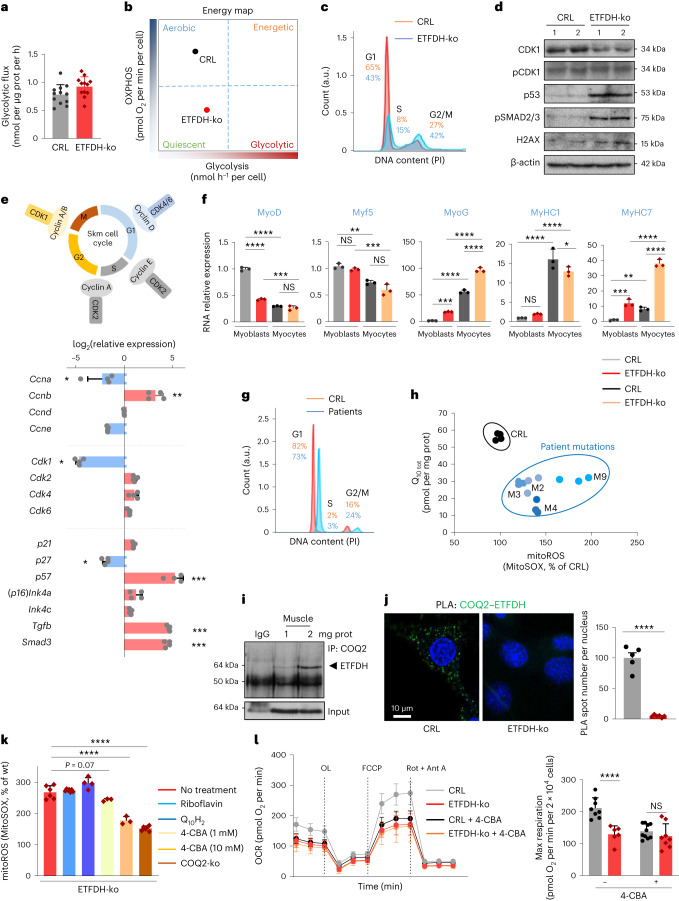
Senescence, aberrant myogenesis and Q deficiency are associated with ETFDH deletion. **a**,**b**, Lactate production (**a**) and energy map (**b**) in CRL and ETFDH-ko myoblasts. *n* = 4. **c**, PI staining of the cell cycle in CRL and ETFDH-ko myoblasts. *n* = 3 independent repeats. Cell percentages in the G1, S and G2/M phases are indicated. **d**, Representative western blot of proteins related to the cell cycle and senescence in two clones of CRL and ETFDH-ko myoblasts. β-Actin is shown as a loading control. **e**, RNA relative expression of cyclins, CDKs and other proteins related to the cell cycle in CRL and ETFDH-ko myoblasts. The upper scheme illustrates Skm cell-cycle regulators. *n* = 3. **f**, RNA relative expression of proteins related to myogenesis (MyoD, Myf5, MyoG, MyHC1 and MyHC7) in CRL and ETFDH-ko myoblasts and myocytes. *n* = 3. **g**, PI staining of the cell cycle in CRL and patient-derived fibroblasts. *n* = 4, three independent repeats. **h**, Relation between total Q_10_ and mitochondrial ROS in CRL and patient-derived fibroblasts. *n* = 4, three independent repeats. **i**, Representative immunocapture (IP) of COQ2 blotted with anti-ETFDH antibody in mouse Skm. Tubulin is shown as a loading control. **j**, Left, representative images of PLA between COQ2 and ETFDH in CRL and ETFDH-ko myoblasts. Right, quantification histogram. Five images per condition, *n* = 3. **k**, Mitochondrial ROS in the presence or absence of the COQ2 inhibitor 4-CBA in ETFDH-ko and double-knockout (COQ2-ko, ETFDH-ko) cells. *n* = 3. **l**, Left, representative respiratory profile of CRL (grey traces) and ETFDH-ko (red traces) myocytes treated or not with 0.5 mM 4-CBA. Glucose was used as a substrate. Right, histogram of the quantification of maximal respiration. *n* = 8 replicates per condition. Results are shown as the mean ± s.e.m. of the indicated *n*. *, **, ***, ****P < 0.05, 0.01, 0.001 and 0.0001 when compared with CRL by two-tailed Student’s *t* test (**a**, **j**), one-way ANOVA with Dunnett’s test (**k**) and two-way ANOVA with Šidák’s test (**f**, **l**). Source data

OXPHOS and glycolysis compensate for each other^37,38^. However, ETFDH deletion caused only a slight increase in glycolytic flux in ETFDH-ko myoblasts (Fig. 3a,b). The cell-cycle profiles (Fig. 3c) and cyclin/cyclin-dependent kinase (CDK) expression panel (Fig. 3d,e) highlighted a G2/M arrest in the absence of ETFDH, compatible with the high ROS levels detected. Additionally, ETFDH-ko myoblasts and fibroblasts appeared senescent, expressing high levels of p53, p57 and phosphorylated SMAD family member 2/3 (pSMAD2/3)/SMAD3 (Fig. 3d). Interestingly, myoblast markers of myogenesis^39^ were altered and the differentiation pattern was compromised and anticipated (Fig. 3f).

**Extended Data Fig. 6:**
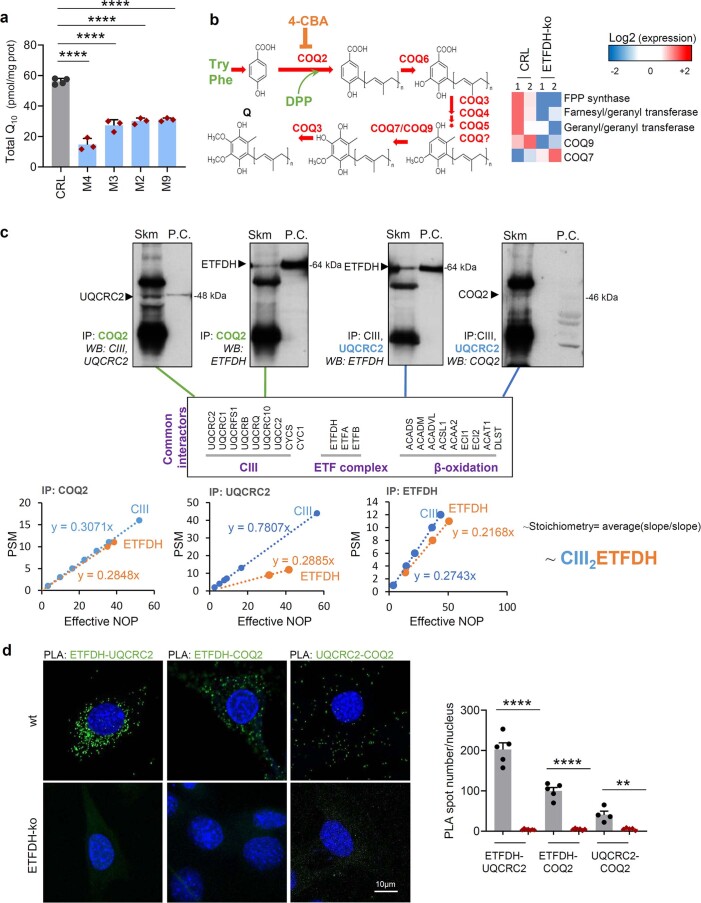
A metabolon comprising ETFDH, CIII, and the regulator of Q biosynthesis, COQ2. **a**) Total Q_10_ levels in control (CRL) and patient-derived fibroblasts (M2, M3, M4, M9). n = 3. **b**) Left panel, schematic represents Q biosynthesis. Right panel, heat-map representation of quantitative proteomic analysis (TMT) of CRL and ETFDH-ko myoblasts. A t-tests to compare groups in a pairwise fashion was used. A (-) log2 p-value > 4 was considered statistically significant. A 3-color palette was used: white, non-significance; progressively more saturated values of blue and red indicate increased significance. Blue and red also means downregulated or upregulated protein, respectively. **c**) Immunocapture (IP) of COQ2 blotted with anti-UQCRC2 and ETFDH antibodies; and IP of UQCRC2 blotted with anti-ETFDH and anti-COQ2 antibodies in Skm extracts. P.C. Positive control (total extract). In the square, common interactors in the proteomic analysis of the 4 IPs. Graphs in lower panel represents the number of tryptic peptides (NOP) plotted against the number of PSM of each protein. The stoichiometry of ETFDH-CIII is estimated by plotting the observed PSM per protein from each complex against their effective NOP; the stoichiometry between the two complexes is estimated as the ratio of the slopes from each one of them. **d**) Proximity ligation assay (PLA) between ETFDH-UQCRC2, ETFDH-COQ2 and UQCRC2-COQ2 in control (CRL) and ETFDH-ko myoblasts. Quantification in right histograms. 5 images/condition, n = 3. Results are shown as the mean ± SEM of the indicated n. *, **, ***, **** p < 0.05; 0.01, 0.001 and 0.0001 when compared to CRL by two-tailed Student’s t-test (d) and one-way ANOVA with Dunnett’s test (a). Source data

**Extended Data Fig. 7:**
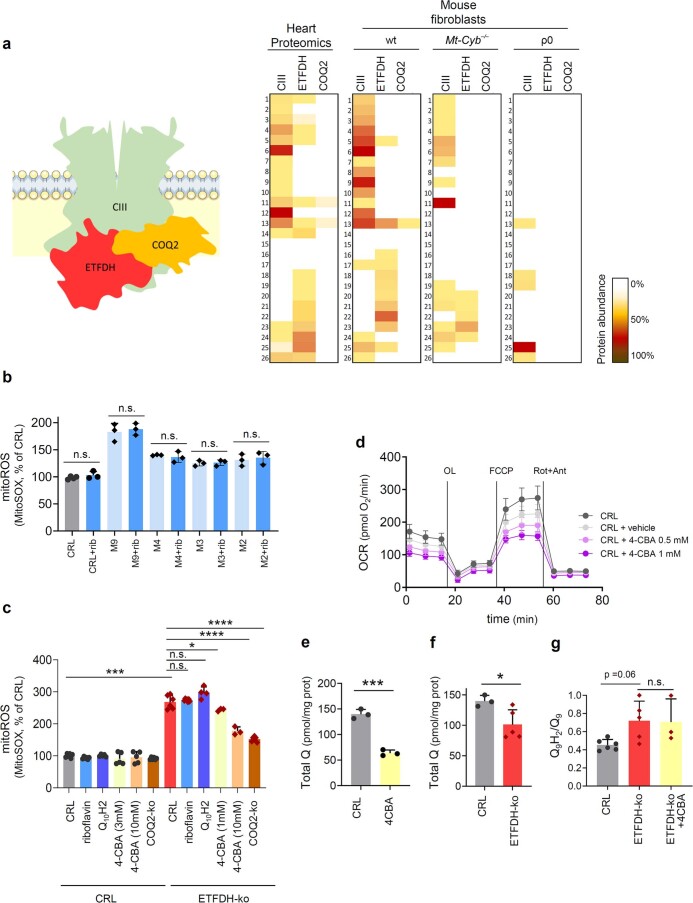
Reducing Q levels as a strategy to counteract reductive stress resulting from dysfunctional ETFDH. **a**) Left panel, schematic represents the metabolon. Right panel, proteomic analysis of 26 bands from 1D-BN-PAGE immunoblots of mitochondrial membrane proteins from wt mouse hearts and wt, *Mt-Cyb*^-/-^ and ρ*0* mouse fibroblasts. White to brown scale indicates the abundance of the protein within a band. ETFDH was found to comigrate with CIII and COQ2 within the band 13 in basal conditions. **b**) Mitochondrial ROS in the presence or absence of riboflavin (rib) in control (CRL) and patient-derived fibroblasts (M2, M3, M4, M9). n = 3. **c**) Mitochondrial ROS in the presence or absence of the COQ2 inhibitor 4-CBA in control (CRL), ETFDH-ko and double COQ2-ko, ETFDH-ko myoblasts. n = 3. **d**) Representative respiratory profile of control (CRL, gray traces) myoblasts treated or not with different doses of 4-CBA. OCR, oxygen consumption rate; OL, oligomycin; FCCP; Rot, rotenone; Ant, antimycin A. Glucose was used as a substrate. 10 replicates/condition. **e**, **f**) Total Q_9_ levels in control in wt and ETFDH-ko myoblasts treated or not with 4-CBA. n = 3. **g**) Q_9_H_2_/Q_9_ ratio in control in wt and ETFDH-ko myoblasts treated or not with 4-CBA. n = 3. Results are shown as the mean ± SEM of the indicated n. *, **, ***, **** p < 0.05; 0.01, 0.001 and 0.0001 when compared to CRL by two-tailed Student’s t-test (b, e, f), one-way ANOVA with Tukey’s test (g), and two-way ANOVA with Dunnett’s test (c). Source data

Surprisingly, fibroblasts from patients with pathological mutations in *ETFDH* or in genes coding for upstream proteins presented normal cell-cycle and proliferation rates (Fig. 3g). In search of an adaptive response in these cells, we found that the total amount of Q was diminished (Fig. 3h and Extended Data Fig. 6a), which is consistent with secondary Q deficiency in patients with MADD^4,18^. This raised the possibility that lowering Q levels in a condition of high QH_2_/Q ratio, such as in the presence of dysfunctional ETFDH, may provide an advantage in terms of hindering reductive stress. Accordingly, several enzymes involved in Q synthesis were found to be downregulated in a quantitative proteomics (tandem mass tag (TMT)) study of ETFDH-ko myoblasts compared with CRL (Extended Data Fig. 6b). More importantly, BN-PAGE in mouse Skm mitochondria (Fig. 1n), co-IP in Skm extracts (Fig. 3i and Extended Data Fig. 6c) and PLA in myoblasts (Fig. 3j) showed a direct interaction between ETFDH and the COQ2 enzyme from the Q-biosynthesis pathway^18,40,41^. This suggests COQ2 as part of the metabolon comprising ETFDH and CIII. Further evidence came from COQ2–UQCRC2 co-IP (Extended Data Fig. 6c) and PLA (Extended Data Fig. 6d). Using a similar approach as before, we portioned a BN gel into 26 bands and used proteomics to identify interacting partners (Extended Data Fig. 7a). COQ2 was exceptionally challenging to detect by proteomics, as it is one of the least abundant proteins within the cell^42^. However, COQ2 was successfully identified in heart extracts and wild-type (wt) mouse fibroblasts within the same band where UQCRC2 and ETFDH were also present (band 13 in Extended Data Fig. 7a). These findings were corroborated in cells exhibiting CIII defects (*Mt-Cyb*^−/−^ and ρ0 fibroblasts). Under these conditions, CIII failed to assemble into supercomplexes (Extended Data Fig. 7a). Intriguingly, in both cases, neither ETFDH nor COQ2 was detected in the band, emphasizing the critical role of CIII in the metabolon-assembly process. Likewise, following the deletion of ETFDH, the number of COQ2–UQCRC2 PLA spots was significantly reduced (Extended Data Fig. 6d), implying that ETFDH is mediating their interaction.

We next hypothesized that the metabolon modulates Q levels to escape reductive stress. To test our hypothesis, we compared decreasing Q biosynthesis with classical MADD treatments (riboflavin or Q_10_H_2_ administration) regarding their capacity to reduce ROS or modify oxygen consumption in our model. While riboflavin or Q_10_H_2_ had almost no effect in myoblasts and patient-derived fibroblasts (Fig. 3k and Extended Data Fig. 7b,c), both pharmacological (4-chlorobenzoic acid (4-CBA) treatment^40^; Extended Data Fig. 6b) and genetic (COQ2-ko) impairment of COQ2 reduced mitochondrial ROS production in ETFDH-ko cells (Fig. 3k). Treatment with 4-CBA in CRL myoblasts reduced oxygen consumption in a dose-dependent manner (Extended Data Fig. 7d), consistent with its capacity to decrease the Q pool^40^ (Extended Data Fig. 7e). Interestingly, this reduction was significantly lower in the absence of ETFDH (Fig. 3l). This suggests that Q biosynthesis is already inhibited following ETFDH deletion. Indeed, Q levels were limited in ETFDH-ko cells compared with CRL (Extended Data Fig. 7f), as in patients with MADD. However, the QH_2_/Q ratio in ETFDH-ko myoblasts was the same in the presence or absence of 4-CBA (Extended Data Fig. 7g), indicating that CIII is inhibited following ETFDH deletion regardless of COQ2 inhibition. Nevertheless, the lower amount of Q after 4-CBA treatment corresponds to less electron escape to oxygen (which means lower ROS production; Fig. 3k). Overall, our data suggest that, rather than being a comorbidity, MADD-associated Q deficiency might represent a cellular strategy to mitigate reductive stress resulting from ETFDH dysfunction.

**Fig. 4:**
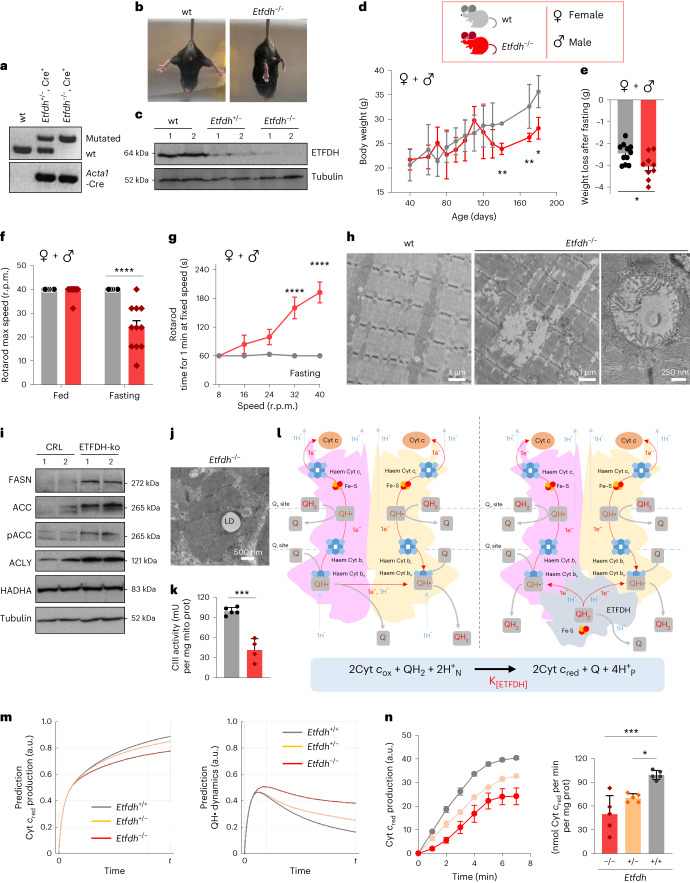
A conditional and Skm-specific *Etfdh*^−/−^ mouse with inhibited CIII activity. **a**, PCR analysis of the native and mutant *Etfdh* and *Acta1*-rtTA-Cre constructs in Skm from wt, heterozygous *Etfdh*^+/−^ and homozygous *Etfdh*^−/−^ mice. **b**, Representative images of wt (left) and *Etfdh*^−/−^ (right) mice. **c**, Western blot analysis of ETFDH protein levels in Skm from wt, *Etfdh*^+/−^ and *Etfdh*^−/−^ mice. **d**,**e**, Body weight over time (**d**) and weight loss after fasting (150-day-old mice) (**e**) in wt and *Etfdh*^−/−^ mice. *n* = 11 mice per genotype. **f**,**g**, Motor behaviour assays in male + female mice: rotarod maximum speed (**f**) and time for 1 min at a fixed number of rotations per minute (r.p.m.) (**g**) in fed and fasting 6-month-old wt and *Etfdh*^−/−^ mice. *n* = 11 animals per genotype (six females and five males). Individual analysis for male and female mice are shown in Extended Data Fig. 8. **h**, TEM images of longitudinal soleus slices from wt and *Etfdh*^−/−^ mice. Extended sarcomere disorganization and debris were observed following ETFDH deletion. Mitochondria appeared aberrant, empty and with cristae disorganization. Images are representative of *n* = 5 mice per genotype. Ten images per mouse. **i**, Representative western blot expression of Skm proteins from de novo lipogenesis in CRL and ETFDH-ko myoblasts. The expression of ATP citrate lyase (ACLY), acetyl-CoA carboxylase (ACC) and its phosphorylated form (pACC), and FA synthase (FASN) is shown. The expression of the hydroxyacyl-CoA dehydrogenase trifunctional multienzyme HADHA is also shown. Two samples per condition; each sample contains protein extracts from three mice. Tubulin is shown as a loading control. *n* = 6 mice per genotype. **j**, TEM images of longitudinal soleus slices from *Etfdh*^−/−^ mice. Intrafibre lipid droplet (LD) surrounded by mitochondria in the subsarcolemma is shown. Image is representative of *n* = 5 mice per genotype. Ten images per mouse. **k**, CIII enzymatic activity in isolated Skm mitochondria from wt and *Etfdh*^−/−^ mice. *n* = 3 mice per genotype. **l**, Current (left) and new version (right) of the Q cycle. The proposed new version involves ETFDH and is composed of two steps. Step 1: as in the current theory, a QH_2_ molecule in each monomer enters the cycle and is reduced to Q; one electron is donated to the high-potential chain in the ISP–Cyt *c*_1_–Cyt *c* axis, and the other electron proceeds in a bifurcated reaction through Cyt *b*_H_ and Cyt *b*_L_ and is donated to one Q molecule, forming the radical QH•. This electron gradient is accompanied by the uptake of one H^+^_N_ from the matrix and the extrusion of two H^+^_P_ to the IMM. The stoichiometry of the reaction for the two monomers is Cyt_ox_ + QH_2_ + Q + 2H^+^_N_ → Cyt_red_ + Q + QH• + 4H^+^_P_. Step 2*:* the second electron is directly donated by ETFDH in two one-electron and one-H^+^ reactions, reducing two molecules of QH• to QH_2_ and liberating one molecule of Q. The stoichiometry of this reaction is QH_2_ + 2QH• + ETFDH_FADH2_ → Q + 2QH_2_ + ETFDH_FAD+_. Therefore, the total stoichiometry is the same as in the current Q cycle, but with the participation of ETFDH: 2Cyt *c*_ox_ + 3QH_2_ + 2Q + ETFDH_FADH2_ + 2H^+^_N_ → 2Cyt *c*_red_ + 3Q + 2QH_2_ + ETFDH_FAD+_ + 4H^+^_P_ = 2Cyt *c*_ox_ + QH_2_ + ETFDH_FADH2_ + 2H^+^_N_ → 2Cyt *c*_red_ + Q + ETFDH_FAD+_ + 4H^+^_P_ = 2Cyt *c*_ox_ + QH_2_ + 2H^+^_N_ → 2Cyt *c*_red_ + Q + 4H^+^_P_. The observation that CIII retains 50% of its activity following ETFDH deletion indicates that CIII can function autonomously, consistent with previous theories. However, this new version proposes a lower QH• standby time, minimizing electron leak and consequent ROS production, thus increasing OXPHOS efficiency. Details are provided in Extended Data Fig. 9. **m**, Mathematical simulations of the Q cycle using the equations in Extended Data Fig. 9. ODEs describe the dynamics of Cyt *c* (left) and Q (right) over time in *Etfdh*^+/+^, *Etfdh*^+/−^ and *Etfdh*^−/−^ conditions, highlighting higher efficiency of CIII in the presence of ETFDH. Details are provided in Extended Data Fig. 10. **n**, Cyt *c*_red_ production over time in isolated Skm mitochondria from wt, *Etfdh*^+/−^ and *Etfdh*^−/−^ mice. Absorbance (a.u) over time (left) and nmol of product per min per mg of protein (right). *n* = 3 mice per genotype. Results are shown as the mean ± s.e.m. of the indicated *n*. *, **, ***, ****P < 0.05, 0.01, 0.001 and 0.0001 when compared with wt by two-tailed Student’s *t* test (**d**, **e**, **k**), one-way ANOVA with Tukey’s test (**n**) and two-way ANOVA with Šidák’s test (**f**, **g**). Source data

**Extended Data Fig. 8:**
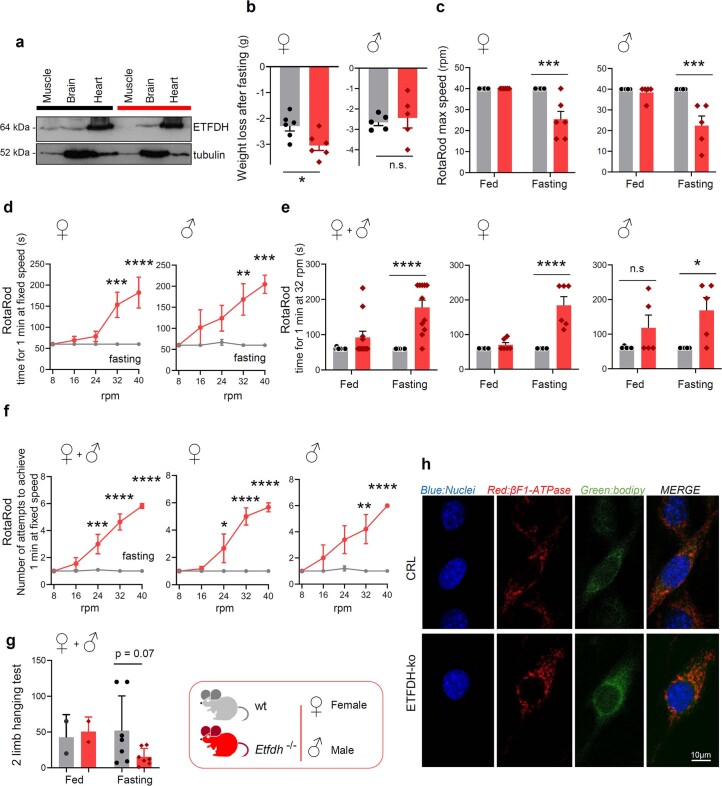
Female and male *Etfdh*-/- mice display myopathy. **a**) Representative western blot analysis of ETFDH protein levels in Skm, brain and heart from wt and *Etfdh* ^-/-^ mice. ETFDH is absent specifically in muscle. **b**) Weight loss after fasting (150 days old mice) of wild-type (wt) in male and female *Etfdh*^-/-^ mice. n = 10 mice/genotype. **c–g**) Motor behavior assays in female (♀) and male (♂) mice. RotaRod max speed (**c**), time for 1 min at fixed rpm (**d**) time for 1 min at 32 rpm (**e**) and number of attempts to achieve 1 min at fixed speed (**f**) in 6 months-old wt and *Etfdh* ^-/-^ mice. In **g**, 2 limb hanging test in 6 months-old wt and *Etfdh* ^-/-^ mice. n = 11 animals/genotype (6 females and 5 males). **h**) Immunofluorescence from wt and ETFDH-ko myoblasts. Red, mitochondria: β-F1-ATPase; blue, nuclei: DAPI; green, lipid droplets: bodipy. Images are representative of 10 pictures/condition. Results are shown as the mean ± SEM of the indicated n. *, **, ***, **** p < 0.05; 0.01, 0.001 and 0.0001 when compared to wt by two-tailed Student’s t-test (b, g), and two-way ANOVA with Sidak’s test (c-f). Source data

**Extended Data Fig. 9:**
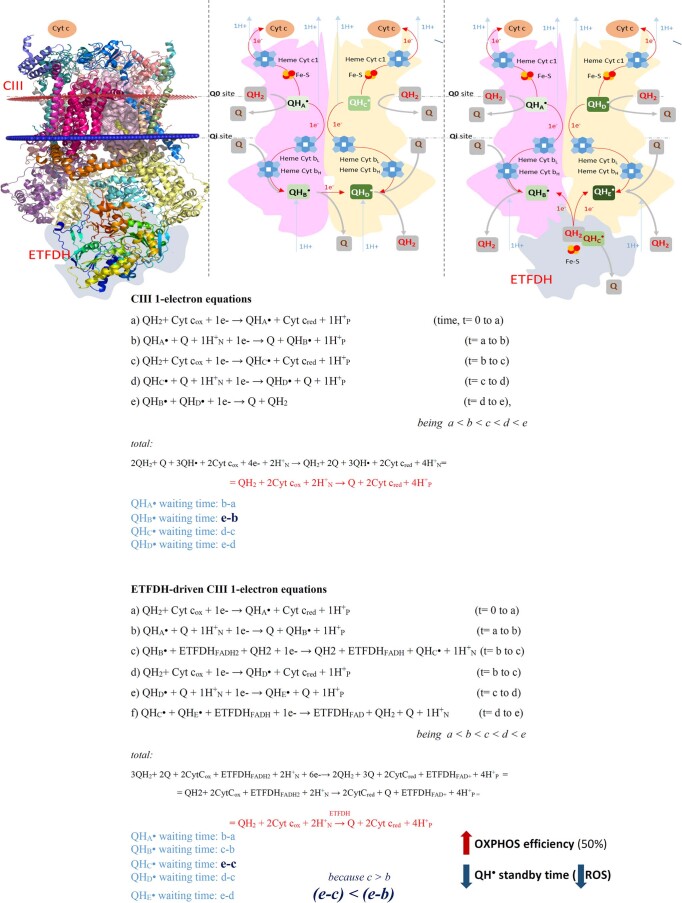
A revised version of the Q cycle involving ETFDH. Current (left panel) and new version (right panel) of the Q cycle. Despite the total stoichiometry is the same than in the current Q cycle, this new version proposes a lower QH• standby time, minimizing electron leak and consequent ROS production, thus increasing OXPHOS efficiency.

**Extended Data Fig. 10:**
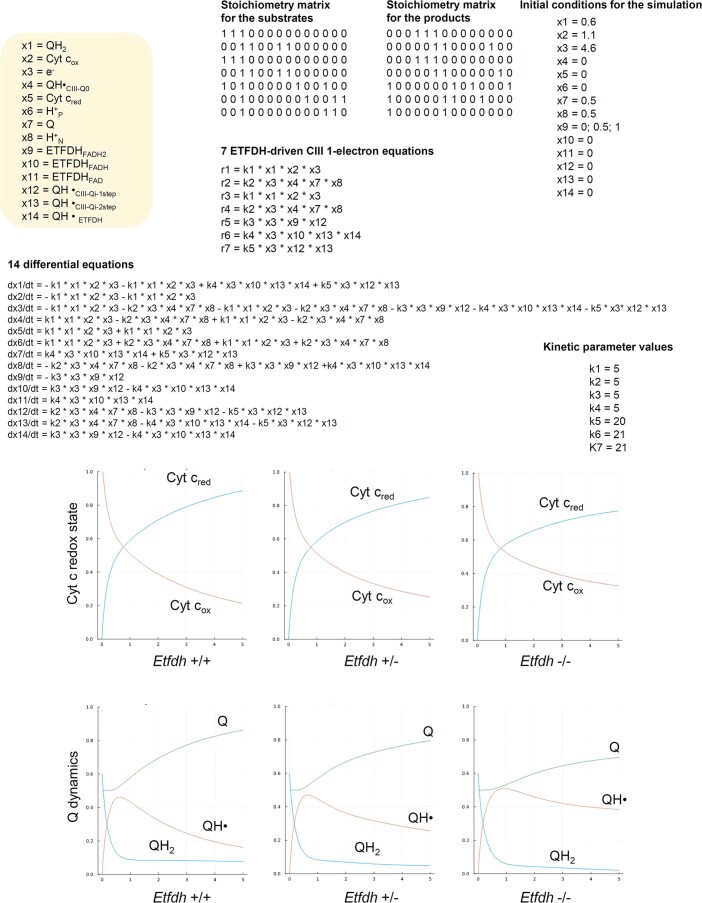
Numerical simulations of the equations in Extended Data Fig.9. The ordinary differential equations (ODEs) describe the dynamics of each of the reactants involved. Kinetic parameter values for the interactions are tuned to ensure changes in the variables during the simulation, and obtain a response that resembles qualitatively the experiments. The results obtained are robust to moderate changes in the values of the kinetic constants chosen. Graphs represent the Cyt c and Q redox dynamics during time in *Etfdh*^+/+^, *Etfdh* ^+/-^ and *Etfdh* ^-/-^ conditions.

To confirm the results in vivo, we generated a conditional and Skm-specific mouse model of ETFDH deletion. To achieve this, we bred the floxed *Etfdh*-Tm1c mouse (Fig. 4a) with the *Acta1*-rtTA-tetO-Cre^+^ mouse^31,43^ (Fig. 4a), which integrates the Cre construct into its genome under an Skm-specific tetracycline-regulated promoter. The resulting *Etfdh*-Tm1c^+/+^-Cre^+^ transgenic animal (Fig. 4a,b; *Etfdh*^−/−^) did not express ETFDH protein specifically in Skm (Fig. 4c and Extended Data Fig. 8a). The hemi-knockout *Etfdh*-Tm1c^+/–^Cre^+^ mouse (Fig. 4a,b; *Etfdh*^+/−^) showed reduced ETFDH expression (30% of wt; Fig. 4c). Following the growth of mice up to day 200, we observed reduced body weight in *Etfdh*^−/−^ mice compared with wt littermates (Fig. 4d) and a poorer response to fasting (Fig. 4e and Extended Data Fig. 8b) and general impairment in 6-month-old *Etfdh*^−/−^ animals (Fig. 4b). This resulted in lower performance in motor behaviour assays such as the rotarod test and two-limb hanging test compared with wt (Fig. 4f,g and Extended Data Fig. 8c–g). On examining the Skm structure using transmission electron microscopy (TEM), we observed fibre shrinkage, sarcomere disorganization and debris in *Etfdh*^−/−^ mice (Fig. 4h), indicating the presence of myopathy. It is worth noting that, in the absence of ETFDH, some mitochondria appeared swollen, empty and with dysfunctional cristae arrangement (Fig. 4h). Moreover, consistent with the inhibition of FAO, *Etfdh*^−/−^ mice presented increased Skm de novo lipid synthesis (Fig. 4i) and interfibre lipid droplets (Fig. 4j and Extended Data Fig. 8h) in close contact with dysfunctional mitochondria (Fig. 4j). Interestingly, this phenotype recapitulates the phenotype observed in Skm from patients with MADD^15,17^. In line with what was observed in ETFDH-ko myoblasts and myocytes, Skm CIII activity in isolated mitochondria from *Etfdh*^−/−^ mice was reduced compared with wt littermates (Fig. 4k). Hence, we defined one-electron equations for describing the observed dependency of CIII activity on the presence of ETFDH (Fig. 4l and Extended Data Fig. 9). To learn how this alternative set of reactions modulates the Q cycle, we developed a system of differential equations that describes the redox dynamics of each of the reactants involved (Extended Data Fig. 10). The resulting 14 differential equations were simulated numerically (Extended Data Fig. 10). The model predicted an increase in Cyt *c*_red_ and a reduced QH• standby time in the presence of ETFDH (Fig. 4m and Extended Data Fig. 10), thus diminishing electron leak to oxygen during OXPHOS (limiting ROS formation, as in Fig. 2) and finally increasing CIII efficiency. Interestingly, Cyt *c*_red_ production in isolated mitochondria from *Etfdh*^+/−^ and *Etfdh*^−/−^ mice was reduced to 73% and 54% of the wt, respectively (Fig. 4n), in qualitative agreement with the model prediction in Fig. 4m.

In light of all the results presented, we can speculate that the iron–sulfur flavoprotein ETFDH is a necessary component of the metabolon that sustains OXPHOS efficiency in skeletal muscle. OXPHOS efficiency in Skm relies on the proper transfer of electrons from reductive equivalents and QH_2_ to the ETC. Any limitation or leakage in the electron flow results in a reduction in ATP generation and an increase in ROS production. In addition, NADH, FADH_2_ and QH_2_, generated through FA and BCAA catabolism in Skm, are potentially oxidized in the reactive environment of the mitochondrial matrix. The organization of the ETC in supercomplexes facilitates the channelling of substrates, limits their spontaneous oxidation and improves OXPHOS efficiency^22,44,45^. Depending on the tissue and the availability of a substrate, evolution may have led to the formation of other metabolons that facilitate electron transfers from specific catabolic pathways. In line with observations on other FAO proteins^24^, we here demonstrate that in Skm, one of the major sites of FA and BCAA catabolism, an FAO–OXPHOS–Q biosynthesis metabolon exists, which improves the catalytic efficiency of energy production.

The lipid electron transporter Q has a key role in metabolism and redox homeostasis^46–48^. However, many aspects of Q biology remain undefined. We hypothesize that the metabolon formed by at least CIII, the ETF complex (ETFDH, ETFA, ETFB) and the Q-biosynthesis regulator COQ2 (ref. ^40^) aims to regulate Q metabolism. Moreover, the dependency of CIII activity on ETFDH suggests that BCAA catabolism and FAO are necessary for OXPHOS in Skm, with ETFDH being an essential ETC component in muscle. When electrons from these catabolic pathways fail to enter the ETC (that is, due to ETFDH deletion), reductive stress is postulated to be reduced^36^. However, the lack of ETFDH resulted in a significant increase in the QH_2_/Q ratio, leading to increased superoxide production. The absence of a Q reductase causing QH_2_ instead of Q accumulation is a paradoxical observation that prompted us to hypothesize an alternative role for ETFDH beyond its classical one. We propose that ETFDH participates in the molecular mechanism of CIII. Consistent with this, deficiencies in either CIII or ETFDH activity generate similar perturbations in both the myoblast proteome and patients’ clinical biochemistry. Additionally, ETFDH alterations have been previously associated with CII–CIII deficiency in the liver and muscle of patients with MADD^49^. Of note, the Q-cycle scheme Mitchell had first suggested^7^ required input of an electron from the dehydrogenases to complete the reduction of Q on the matrix side. With CII considered as the involved dehydrogenase, the major criticisms about Mitchell’s Q cycle were as follows: (1) no CII–CIII binding was observed with the technology of the time; (2) CIII activity does not require CII; (3) Ant A does not inhibit CII; and (4) the isolated Cyt *bc*_1_ complex (prokaryotic CIII analogue) works autonomously with a self-contained mechanism^5,6^. In this study, we used different approaches to demonstrate that (1) Skm CIII interacts with ETFDH; (2) CIII activity is dependent on ETFDH; and (3) both CIII inhibitors, Ant A and myxothiazol, inhibit ETFDH activity^50^ and FAO^51,52^. The observation that CIII retains ⁓40–50% of its activity following ETFDH deletion means that it can still function autonomously (Extended Data Fig. 9), consistent with Garland’s previous findings. Incorporating the dimeric nature of the CIII complex^53^ and the latest theories on the Q-cycle mechanism of action^5^, our findings support a model whereby Skm ETFDH may help in reducing the semiquinone QH• produced at the CIII Q_i_ site. Notably, ETFDH is reduced by ETF in two one-electron transfer half-reactions^11^, generating and stabilizing QH• within its structure^54^. The ETFDH Q-binding mechanism differs from other Q reductases, such as CII^55^, which have conserved domains with Tyr/Trp or His residues forming hydrogen bonds with O1 and/or O4 of the benzoquinone ring. In the ETFDH Q-binding domain, no hydrogen bond is formed between the O1 atom and amino acids^11^, suggesting a rapid electron transfer to CIII during the Q cycle. Although the final stoichiometry of the reaction is the same as in the classical Q cycle (Extended Data Fig. 9), this new version proposes a lower QH• standby time, which minimizes electron escaping and ROS production and thus increases OXPHOS efficiency. Further studies are needed to reveal other interactors of the metabolon or a possible allosteric regulation of CIII through its binding with ETFDH, or whether the loss of this binding might cause a conformational change similar to the one produced by CIII inhibitors^56^. However, several similarities exist between Ant A (or myxothiazol) and the absence of ETFDH: both inhibit CIII activity, increase the QH_2_/Q ratio^36^, modify the redox state of Cyt *b*^57^ and boost ROS^29^, suggesting a similar mechanism of action at the Q_i_ site. In support of this hypothesis, the introduction of a mitochondrial AOX, which decreased QH_2_ and ROS levels^23^, did not restore ∆Ψm, OCR and CIII activity, thus ruling out a direct effect of ROS on CIII. Furthermore, the ETFDH substitutions Y271A and G273E, which hinder Q binding but maintain the ETFDH structure, resulted in a similar increase in superoxide production as the complete deletion of ETFDH. This finding further supports the necessity of a functional ETFDH for proper CIII activity.

Mutations in *ETFDH*, *ETFA* and/or *ETFB* have been associated with MADD^12–14,16^ and secondary Q deficiencies^16,18,58,59^. Current treatments are limited to low-FA diets. Only a few patients respond to riboflavin, carnitine or Q_10_ supplementation^12,13,58^, despite efforts to enhance cellular Q absorption^18^ and the success of oral Q_10_ treatment in primary Q deficiency^18^. Consistent with these clinical data, mouse ETFDH-ko myoblasts and patient-derived fibroblasts do not respond to either riboflavin or Q_10_H_2_ administration. In contrast, in our model, inhibiting Q biosynthesis, either pharmacologically or genetically, was beneficial for reducing ROS production. This may be related to the observed CIII–ETFDH–COQ2 metabolon. We speculate that the disruption of the complex inhibits the synthesis of Q in an attempt to minimize the QH_2_-driven reductive stress generated by CIII dysfunctions in the absence of ETFDH. Accordingly, the Q-biosynthesis enzymes COQ9, farnesyl pyrophosphate synthase (FDPS), farnesyltransferase α-subunit (FNTα) and Rab geranylgeranyltransferase subunit α (RabGGTα)^60^ are downregulated in our model, and ETFDH-ko myoblasts presented reduced Q levels. Interestingly, and similarly to what occurs in patients with MADD and in *Etfdh*^−/−^ mice, alterations in COQ9 have been associated with dysfunctions in CI + III and CII + CIII activity, leading to myopathies^61,62^. Likewise, patients with MADD have been demonstrated to have multiple ETC enzyme deficiencies^15,17^, likely due to instability of the metabolon. Hence, we speculate that Q_10_H_2_ supplementation may be counterproductive for treating MADD, as Q deficiency in MADD is a cellular compensatory response to ETFDH-mediated reductive stress.

Overall, our results highlight ETFDH as an ETC component and offer molecular insights into how ETFDH regulates OXPHOS efficiency, providing a new therapeutic target for refined mitochondrial redox medicine.

## Methods

### Reagents

A comprehensive list of reagents, primers and antibodies used is detailed in Supplementary Table 2.

### Ethical considerations

#### Human

All human studies were performed following EU ethical guidelines and approved by institutional committees (UAM University and Madrid Community, Spain; CEI-129-2655, CEI-105-2052). We obtained informed consent from all participants. Participants did not receive compensation for participating in this study.

#### Mice

Animal studies were performed following EU ethical and ARRIVE (Animal Research: Reporting of In Vivo Experiments) guidelines. Procedures were approved by institutional review boards (UAM University and Madrid Community Ethical Committees, Spain; PROEX 183/17, PROEX 207.5/22).

### Patient-derived cells and samples

Patient (paediatric age)-derived cells and samples (urine, blood) were collected for genetic diagnosis from individuals suspected of having an inborn error of metabolism at Centro de Diagnóstico de Enfermedades Moleculares, Spain. Primary skin fibroblasts from paediatric patients (one female, two males, one with unreported sex) and CRLs (male) were cultured in DMEM (4.5 g l^−1^ glucose) supplemented with ʟ-glutamine, 10% FBS and 1% penicillin/streptomycin. No additional information on population characteristics was collected for the study. Cultures were maintained at 37 °C and 5% CO_2_ following standard procedures. CRL fibroblasts (NHDF (Neonatal Human Dermal Fibroblasts), male) and human fibroblasts with mutations in *ACAD9* (M1, heterozygous for c.359delT corresponding to p.Phe120Serfs*9 and c.796C>T corresponding to p.(Arg266Trp)), *ETFDH* (M2, heterozygous for p.Val451Leu and p.Term618Gln; female), *BCKDHB* (M3, homozygous for c.348delA, corresponding to p.Lys116fs; male) and *DBT* (M4, heterozygous for p.Ala422Ser and p.Asp145Glufs*28; male) were used. For in vitro experiments, samples were individually treated (not pooled).

### Animal studies

The B6;C3-Tg(*Acta1*-rtTA,tetO-cre, Skm-Cre mice)102MonK/J mouse was purchased from The Jackson Laboratory. The *Etfdh*-tm1a (EUCOMM) Hmgu (European Mouse Mutant Archive (EMMA) ID: 09069) mouse was purchased from EMMA INFRAFRONTIER and bred with the C57BL/6-FLPe mouse to obtain the *Etfdh*-Tm1c mouse (E mice). *Etfdh*^−/+,Acta1-Cre+^ (*Etfdh*^+/−^) and *Etfdh*^−/−,Acta1-Cre+^ (*Etfdh*^−/−^) mice were obtained by breeding Skm-Cre with E mice for two generations. Mice were maintained on the (C57BL/6x C3H)F2 background. Animals were maintained in a 12-h light/12-h dark cycle at ~18–23 °C with 40–60% humidity. Doxycycline (2 mg ml^−1^) was administered in drinking water for at least 1 week to induce Skm expression of the Cre protein. All experiments were performed on age-matched 6-month-old male and female littermate wt and *Etfdh*^−/−^ mice.

To minimize the number of animals, we used power analysis to calculate the minimum sample size using the free software DOEUMH (https://samplesizeumh.shinyapps.io/DOEUMH) based on the TrialSize library of the R program (R Core Team). We selected the procedure KMeans—analysis of variance (ANOVA), with significance fixed at 0.05, power set to 0.08 and an anticipated dropout of 5%. We considered differences between averages of about 1.5- to 2-fold. The minimum number of mice per group was eight.

Mouse motor functions were evaluated using (1) a rotarod test and (2) a two-limb hanging test^43^. All tests were performed in a blinded fashion. Randomization was assessed by equally distributing experimental groups across multiple cages and balancing the location of the mouse cages on the racks.

#### Rotarod test

The rotarod test was performed in 3 days. Mice were trained during the first and second days, and the test was performed on the third day. For all tests, a soft padded surface was placed at the base of the apparatus to cushion any fall of the animals.

Day 1 was used to train the mice in four sessions of 2-min walking at 4 r.p.m. The three resting times were (1) 30 min, (2) 1 h and (3) 30 min.

Day 2 (training) was identical to day 1, but the walking speed was set to 4 r.p.m. and gradually increased to 12 r.p.m.

On day 3 (test), the starting speed was 4 r.p.m. and the maximum speed was 40 r.p.m. The animals were allowed to stay on the rod until they fell off. Speed was increased after the mice completed 1 min at a given speed. The time taken to complete 1 min at each speed was measured, as well as the maximum speed at which 1 min on the rod was completed. The experiment ended when mice had fallen off six times or when 1 min at 40 r.p.m. was achieved.

#### Hanging test: two limbs

Forelimb muscle strength was measured by monitoring the ability of mice to exhibit sustained limb tension to oppose their weight. Mice were placed in a 2-mm-thick metal bar at 35 cm of a padded surface, and the time until falling was recorded. The test ended after a hanging time of 2 min was achieved or otherwise after three sessions. The longest hanging time (s) and minimal holding impulse (body mass × hang time) were calculated.

#### Tissue isolation

After being killed, wt, *Etfdh*^+/−^ and *Etfdh*^−/−^ mice were perfused with 1× PBS. The heart, liver, brain, and soleus and gastrocnemius muscles were dissected after removing the animals’ hair, skin and fascia, as in ref. ^63^.

### Cell culture and growth assays

All cells were cultured at 37 °C in a 5% CO_2_ atmosphere in a Thermo Forma incubator. Mouse-derived primary myoblasts and C2C12 mouse myoblasts (ATCC catalogue no. CRL-1772) were cultured in DMEM supplemented with 10% FBS, 2 mM glutamine, 100 U ml^−1^ penicillin, 0.1 mg ml^−1^ streptomycin and 400 μM nonessential amino acids. CRL and AOX-expressing mouse fibroblasts (kindly gifted by J.A. Enriquez) were cultured in the same DMEM with 5% FBS. Patient-derived fibroblasts were cultured in minimum essential medium (MEM) supplemented with 10% FBS, 1 mM glutamine and amino acids.

When indicated, cells were grown in different glucose concentrations (0.5, 4.5 and 10 mM) or without BCAA, FA and choline. For other experiments, glucose-free DMEM was supplemented with 50 μM palmitate, 50 μM oleate, 600 μM leucine, 600 μM isoleucine or 600 μM valine.

#### Primary cultures of myotubes

Primary Skm cell cultures (myotubes) were established from hindlimb muscle biopsies from wt or *Etfdh*^−/−^ male mice. Cells were propagated in SkGM medium (Lonza) and supplemented with the accompanying bullet kit but with insulin omitted. At 80–90% confluence, cells were differentiated in α-MEM containing 2% FBS and 100 nM insulin. Differentiation of C2C12 mouse myoblasts was assessed at 80% confluence by changing the medium to D medium (DMEM + 2% FBS, 1 mM glutamine and amino acids, 100 nM insulin) for at least 72 h.

Cell growth was assessed by monitoring the number of live cells over time by measuring the fluorescence emission of calcein (Invitrogen, 1 μM) in a FLUOstar microplate reader (BMG Labtech, *λ*_ex_ = 490 nm and *λ*_em_ = 515 nm).

### Recombinant DNA, cloning and transfection

Amplification of the *Etfdh* gene or its mutated variants was performed with a high-fidelity polymerase kit (CloneAmp HiFi PCR Premix, Takara) using 100 ng of cDNA and the corresponding primers (Supplementary Table 2; 5 μM). PCR product analysis was performed by agarose-gel fractionation using Gel-Red (Biotium) as a dye and 1-kb Plus Ladder (Thermo Fisher Scientific) as a band-size reference.

Amplified genes and pcDNA3.1 and pCMV-Sport6 plasmids were digested using HindIII and EcoRI restriction enzymes (1 h at 37 °C followed by 20 min at 65 °C to inactivate the enzymes). The digested parts were ligated at a 1:3 insert/plasmid ratio with ligase T4 (Thermo Fisher Scientific, 1 h). Amplified genomic DNA fragments were cloned using the pGEM-T Easy Vector system (Promega). Checking of DNA fragments inserted into pGEM-T, pSpCas9(BB)-2A-GFP or mouse genotyping was done using the GoTaq polymerase (Promega) with different primers (Supplementary Table 2).

#### Plasmid and genomic DNA extraction

Plasmid extraction was performed using the Wizard Plus SV Minipreps DNA purification system (Promega) or the Plasmid Maxi kit (Qiagen) following the manufacturer’s instructions. Purification of DNA bands on agarose gels was done with the Wizard SV Gel and PCR Clean-Up System (Promega). For mouse genomic DNA extraction, 1-mm^3^ tail or ear samples were used. Briefly, DNA was extracted using KAPA Express Extract Enzyme and KAPA Express Extract Buffer (Roche) following the manufacturer’s instructions.

#### Transient transfection

At ~70% confluence, 3 × 10^5^ cells were transfected with CRL or ETFDH plasmids (pcDNA3.1(+) and pCMV-SPORT6 plasmids were used^64^) using Lipofectamine 3000 (Invitrogen) or FuGENE (Promega) transfection reagents following the manufacturer’s instructions. Details of plasmid constructs are reported in Supplementary Table 2. Experiments were performed 24–48 h after transfection.

### Generation of stable knockout myoblasts

CRISPR–Cas9 technology was used as described in ref. ^65^ to generate stable knockout cell lines for the genes *Etfa*, *Etfb*, *Etfdh*, *Prodh*, *Gpd2*, *Dld*, *Dhodh* and *Coq2*. Single-guide RNAs (sgRNAs) were obtained using an online CRISPR design tool (http://www.rgenome.net/cas-designer/), targeting exon 2 of the selected genes (sequences are available in Supplementary Table 2). sgRNAs were cloned into pSpCas9(BB)-2A-GFP plasmid (Addgene ID: 48138) using the BbsI restriction enzyme, as described above. The designed primers (10 μM) were aligned (1-h incubation at room temperature (RT) on 10× NEB 2.1 buffer). Generated constructs were sequenced by Macrogen. Cells were transfected with this plasmid and incubated for 48 h. Next, cells were sorted by flow cytometry, and green fluorescent protein (GFP)-expressing cells were selected and cultured in previously described conditions. The gene knockout mediated by CRISPR–Cas9 was checked by western blotting or sequencing (when no specific antibodies were available).

### qPCR

RNA was extracted using TRIzol reagent (Thermo Fisher Scientific) following standard TRIzol/chloroform protocols and quantified using NanoDrop. Purified RNA (1 μg) was retrotranscribed into cDNA using the High-Capacity cDNA Reverse Transcription Kit (Thermo Fisher Scientific) following the manufacturer’s instructions.

qPCR was performed using Fast SYBR Master Mix and the ABI Prism 7900HT sequence detection system at the Genomics and Massive Sequencing Facility (Centre for Molecular Biology Severo Ochoa (CBMSO), UAM). β-Actin (*Actb*) and glyceraldehyde phosphate dehydrogenase (*Gapdh*) were selected as housekeeping genes to normalize the mRNA levels. All primer sequences are included in Supplementary Table 2. Standard curves with serial dilutions of pooled cDNA were used to assess the amplification efficiency of the primers and to establish the dynamic range of cDNA concentration for amplification. SDS 2.4 software was used for data collection, and the relative expression of mRNAs was determined with the comparative ΔΔCt method.

### Mitochondria purification

#### Cell-derived mitochondria

Cells were resuspended in a hypotonic medium (83 mM sucrose, 10 mM MOPS, pH 7.2) at a 7:1 buffer/pellet ratio (v/v). After 2 min of incubation on ice, mitochondria were homogenized in a glass–glass homogenizer, diluted in half in a hypertonic medium (250 mM sucrose, 30 mM MOPS, pH 7.2) and centrifuged (1,000*g*, 5 min, 4 °C) to discard intact cells. The supernatant was homogenized and centrifuged again to discard nuclei (1,000*g*, 5 min, 4 °C). The resulting supernatant was centrifuged (12,000*g*, 15 min, 4 °C), and the pellet was immediately used for experiments or stored.

#### Skm mitochondria

Fresh collected hindlimb muscles from wt and *Etfdh*^−/−^ mice were minced and homogenized in a glass–glass homogenizer in buffer A. Skm mitochondria were obtained by centrifugation^64^. Briefly, unbroken cells and tissue were removed by centrifugation at 1,000*g* for 5 min at 4 °C; mitochondrial pellets were obtained by supernatant centrifugation at 11,000*g* for 15 min at 4 °C.

For experiments, mitochondria were resuspended in buffer A (320 mM sucrose, 1 mM EDTA and 10 mM Tris–HCl, pH 7.4) or respiration buffer (225 mM sucrose, 5 mM MgCl_2_, 10 mM KCl, 10 mM phosphate buffer, 1 mM EGTA, 0.05% BSA and 10 mM Tris–HCl, pH 7.4).

### OCR

The OCR in myotubes, fibroblasts and C2C12 cells was determined in an XF96 extracellular flux analyser with the XFe96 Flux Pack (Agilent) using glucose (10 mM) as a substrate following the manufacturer’s protocols. For OCR determination in the presence of palmitate, cells were starved for 12 h in low-glucose DMEM (0.05 mM glucose, 1% FBS). Then, the medium was changed to KHB (111 mM NaCl, 4.7 mM KCl, 1.25 mM glutamine, 5 mM HEPES, pH 7.4). BSA-conjugated palmitate (1 mM sodium palmitate, 0.17 mM BSA solution) was added as the main substrate. To assess the effect of COQ2 inhibition on the OCR, we treated the cells with 4-CBA (0.5 or 1 mM) for 24 h.

In all assays, the final concentration and order of injected substances was 3 μM oligomycin (OL), 5 µM carbonyl cyanide 4-(trifluoromethoxy)phenylhydrazone (FCCP), 1 μM Rot and 1 μM Ant A.

### Measurement of OXPHOS enzymatic activities

Isolated mitochondria (100 µg) were used for the spectrophotometric determination of respiratory complexes CI–CIV, according to ref. ^66^, with minor modifications.

CI activity was measured by monitoring absorbance at 340 nm. Mitochondria were resuspended in 1 ml of CI/CII buffer (25 mM K_2_HPO_4_, 5 mM MgCl_2_, 3 mM KCN and 2.5 mg ml^−1^ BSA) containing 0.1 mM Q_1_, 0.1 M NADH and 1 mg ml^−1^ Ant A. Activity inhibition was accomplished by adding 1 μM Rot.

CII activity was measured by monitoring absorbance at 600 nm. Mitochondria were resuspended in 1 ml of CI/CII buffer containing 30 µM 2,6-dichlorophenolindophenol, 1 µM Rot, 1 µM Ant A, 10 mM succinate and 6 mM phenazine methosulfate. Mal (100 μM) or carboxin (100 μM) was used to inhibit CII.

CIII activity was measured by monitoring absorbance at 550 nm using the Mitochondrial Complex III Activity Assay Kit (Sigma-Aldrich). Ant A (1 μM) was used to inhibit CIII activity.

CIV activity was measured by monitoring the oxidation of the NaBH_4_-reduced Cyt *c* at 550 nm in KP buffer (10 mM phosphate buffer, pH 7). CN^−^ (1 mM) was used as a CIV inhibitor.

### ROS and ΔΨm determination

Superoxide (mitochondrial ROS) or H_2_O_2_ (cytosolic ROS) production in myoblasts, myocytes and other cells was monitored by flow cytometry using 5 μM MitoSOX or H2DCFDA dyes, respectively^31^ (Supplementary Fig. 2). Cells were analysed in a BD FACScan. Where indicated, ETC inhibitors were added at the following concentrations: 1 μM Rot, 100 μM Mal and 1 μM Ant A. When indicated, cells were treated with etomoxir (10 or 100 μM) for 16 h or 4-CBA (3 or 10 mM) for 24 h. At least 10,000 cells were analysed per triplicate, and data were analysed using FlowJo software v10.6.2.

ΔΨm was determined by flow cytometry using tetramethylrhodamine methyl ester (TMRM, Thermo Fisher Scientific).

### Cell-cycle analysis

Cells were fixed with iced 70% ethanol for at least 12 h, washed and stained in cell-cycle buffer (BD Pharmigen) supplemented with 100 μg ml^−1^ RNase A and 50 μl propidium iodide (PI) on PBS. After 30 min of incubation, cells were analysed by flow cytometry.

### NAD, NADP and lactate determinations

Metabolites were measured in 3 × 10^5^ cells or 40 mg of hindlimb muscle from wt and *Etfdh*^−/−^ mice using a FLUOstar Omega spectrophotometer (BMG Labtech). NAD/NADH and NADP/NADPH quantification kits (Sigma-Aldrich) were used to assess NAD/NADH and NADP/NADPH levels following the manufacturer’s instructions. Absorbance was read at 450 nm, and fluorescence at ex/em = 540/590 nm was measured.

#### Glycolytic flux

The rates of aerobic glycolysis in C2C12 cells were monitored spectrophotometrically by quantifying lactate production over time. At different time points, culture medium aliquots were precipitated with 6% perchloric acid for 45 min on ice. After neutralization with KOH, the reaction was initiated in lactate buffer (1 M glycine, 0.4 mM hydrazine, 1.3 mM EDTA, pH 9.5) by adding NAD and lactate dehydrogenase enzyme. After 40 min, absorbance was measured at 340 nm.

### FAO

Four days after differentiation, primary myotubes derived from wt or *Etfdh*^−/−^ mice or C2C12 cells were incubated in serum-free α-MEM containing [9,10-^3^H(N)]palmitic acid (Perkin Elmer, 0.2 µCi, final concentration = 20 µmol l^−1^). After incubation, 100 µl of the culture medium was placed over an ion-exchange resin, and the Poly-Prep chromatography column was washed with water. Intact FFAs (charged state) were retained by the resin, whereas the oxidized portion passed freely^64^. The oxidized portion was measured in a scintillation counter with Ultima Gold LLT scintillation fluid (Perkin Elmer). All results were adjusted for total cellular protein.

### [^14^C(u)]leucine catabolism

For the measurement of substrate catabolism, total oxidation and CO_2_ production were analysed in myoblasts and myocytes incubated in HBSS containing 0.3 mM l-leucine or 5 mM d-glucose + 2 μCi per ml of labelled [^14^C(u)]l-leucine or [^14^C(u)]d-glucose (Perkin Elmer). For CO_2_ production, incubation was carried out in flasks in the presence of a paper filter imbibed in 0.2 M KOH/NaOH solution. The [^14^C]CO_2_ activity was measured in a scintillation counter with Ultima Gold LLT scintillation fluid (Perkin Elmer).

### Q amount and redox-state analysis

Quantification of total Q_9_ and Q_10_ levels and analysis of the redox state (Q_*x*_/Q_*x*_H_2_) in C2C12 cells and patient-derived fibroblasts were performed by high-performance liquid chromatography (HPLC)–tandem mass spectrometry (MS/MS), as described elsewhere^67^, in collaboration with the Instituto de Investigación en Ciencias de la Alimentación.

#### Q extraction from cell cultures

To extract Q from cells, the cell suspension in 200 μl PBS was transferred to an ice-cold extraction solution (200 μl acidified methanol and 300 μl hexane), followed by vortexing. The Q-containing hexane layer was separated by centrifugation (5 min, 17,000*g*, 4 °C) and then dried in a SpeedVac system at 4 °C for 30 min. Dried samples were then resuspended in methanol containing 2 mM ammonium formate, overlaid with nitrogen and stored at −20 °C until analysis.

#### Q and QH_2_ analyses by LC–MS/MS

For LC–MS/MS analysis, a Thermo Scientific instrument consisting of an Accela HPLC pump/autosampler coupled to a Quantum Access triple quadrupole mass spectrometer equipped with an electrospray ionization (ESI) source was used. Chromatographic separation was performed using a Mediterranea C18 column (150 mm × 2.1 mm, 3 μm) from Teknokroma. The mobile phase was composed of 2 mM ammonium formate in methanol (solvent A) and isopropanol (solvent B). A 5-μl aliquot of the sample was injected at a flow rate of 0.3 ml min^−1^ during gradient elution. The gradient programme was as follows: 0 min, 0% B; 1 min, 0% B; 5 min, 100% B; 15 min, 100% B; 16 min, 0% B; 17 min, 0% B.

The mass spectrometer and ESI source were used in positive mode, with the following parameters: skimmer offset (0 V), sheath gas (20 a.u. (arbitrary units)), auxiliary gas (5 a.u.), capillary temperature (350 °C), ion sweep cone gas (0 a.u.) and spray voltage (4,500 V). The target Q species were directly introduced into the ion source, and the collision energies of the selective reaction monitoring transitions were optimized for each species. The first mass transition was used for quantification, and the second was used for identification and confirmation. The transitions used for quantification were as follows: Q_9_, 812.9 > 197.2 (35 eV); Q_10_, 880.9 > 197.2 (34 eV); Q_9_H_2_, 814.9 > 197.2 (35 eV); Q_10_H_2_, 882.9 > 197.2 (35 eV). The peak width was 0.7 Da in both quadrupoles 1 and 3, and the argon pressure in the collision cell (quadrupole 2) was set to 0.002 mbar. Q quantitation was performed using Thermo Xcalibur software. External calibration was used for sample quantification.

### MT-CYB redox-state measurement

The redox state of Cyt *b* in isolated mitochondria from wt and ETFDH-ko myocytes was measured in a Novaspec II spectrophotometer by following the change in absorbance at *λ* = 565 ± 5 nm after adding 100 nM dithionite (DTT)^68^.

### Immunofluorescence and PLA

Hindlimb Skm from wt and *Etfdh*^−/−^ mice was sliced, histologically prepared and stained with haematoxylin and eosin by the Histology Facility at Centro Nacional de Biotecnología (CNB)–Consejo Superior de Investigaciones Científicas, UAM University, Spain. Deparaffination was performed at 60 °C for 1 h, followed by hydration (xylene, ethanol 100%, ethanol 90%, ethanol 70% and distilled H_2_O). C2C12 cells were grown on coverslips. PLA experiments were performed using the Duolink In Situ Green Starter Kit Mouse/Rabbit (Sigma-Aldrich) following the manufacturer’s instructions. For immunofluorescence imaging, cells were fixed with PBS/2% paraformaldehyde (PFA) for 5 min (RT) followed by 20 min at 4 °C with PBS/4% PFA. Autofluorescence was eliminated with 10-min incubation (RT) with PBS/1 mg ml^−1^ NaBH_4_, pH 8. Cells were permeabilized with PBS/0.1% Triton X-100 (10 min, RT) and incubated in blocking buffer (PBS supplemented with 1% BSA, 1% horse serum and 0.1% Triton X-100) for 30 min at RT. Primary antibodies and fluorescent secondary antibodies diluted in blocking buffer were used (see Supplementary Table 2 for a complete list of antibodies used). DAPI (1 μg ml^−1^) was used to stain cell nuclei, and 2 μM BODIPY 493/503 was used to stain lipids. Images were acquired on a Leica DMRE light microscope or by confocal microscopy using a Bio-Rad Radiance 2000 Zeiss Axiovert S100TV. ImageJ 1.53t software was used for quantification and image analysis.

### TEM

Sample preparation was performed by the Electron Microscopy Facility at the CBMSO, UAM University, Spain. Muscles were fixed with 4% PFA and 2% glutaraldehyde in 0.1 M phosphate buffer and treated with 1% osmium tetroxide at 4 °C for 1 h. Then, they were dehydrated with ethanol and embedded in TAAB812 epoxy resin. Ultrathin (80-nm) sections of the embedded tissue were obtained using an ultramicrotome (Ultracut E, Leica) and mounted on carbon-coated copper 75-mesh grids. The sections were stained with uranyl acetate and lead citrate and examined at 80 kV in a JEOL JEM 1010 electron microscope. Images were recorded with a TemCam F416 (4k × 4k) digital camera from TVIPS. Sarcomere and mitochondrial measurements were performed using ImageJ 1.53t software.

### 1D and 2D SDS–PAGE

#### Western blot

Skm, brain, heart and liver samples were freeze-clamped in liquid nitrogen. Tissue or myocyte proteins were extracted in lysis buffer (50 mM Tris–HCl, 1% NaCl, 1% Triton X-100, 1 mM DTT, 0.1% SDS and 0.4 mM EDTA, pH 8.0) supplemented with protease and phosphatase inhibitor cocktails. Supernatants were fractionated by SDS–PAGE and transferred onto polyvinylidene fluoride (PVDF) or nitrocellulose membranes for immunoblot analysis. The primary monoclonal antibody anti-β-F1-ATPase (1:20,000) was developed in our laboratory^69,70^. Other antibodies used are listed in Supplementary Table 2. Blots were revealed using the Novex ECL HRP Chemiluminescent reagent, and the intensity of the bands was quantified using a Bio-Rad GS-900 densitometer and ImageJ 1.53t analysis software.

#### Isoelectric focusing (IEF)

IEF was performed with 13-cm Immobiline DryStrips 3–10 NL (not linear) pH range using an Ettan IPGphor 3 IEF unit (GE Healthcare). In brief, 200 µg of protein from fresh-frozen Skm, diluted in 250 µl of rehydration buffer (DeStreak Rehydration Solution, GE Healthcare) containing 0.5% of the corresponding IPG buffer (GE Healthcare), was loaded on the 13-cm strips. The equilibrated strips were transferred to the top of a 9% SDS–polyacrylamide gel. Electrophoresis was carried out using a PROTEAN II xi system (Bio-Rad) with constant current (30 mA per gel) at 4 °C for 3 h. Western blot analysis of the fractionated proteins was performed as described above.

### 1D BN-PAGE followed by 2D SDS–PAGE

#### First dimension

Isolated mitochondria from C2C12 cells or from Skm of wt and *Etfdh*^−/−^ mice were suspended in 50 mM Tris–HCl, pH 7.0, containing 1 M 6-aminohexanoic acid at a final concentration of 10 mg ml^−1^. Membranes were solubilized by adding 10% digitonin (4:1 digitonin/mitochondrial protein). Serva Blue G dye (5%) in 1 M 6-aminohexanoic acid was added to the solubilized membranes. NativePAGE Novex 3–12% Bis–Tris protein gels were loaded with 30 μg of mitochondrial protein. Electrophoresis was performed at a constant voltage of 70 V for 15 min, followed by 1 h at a constant amperage of 10 mA. BN cathode buffer: 50 mM tricine, 15 mM Bis–Tris, pH 7.0, 0.02% Serva Blue G. BN anode buffer: 50 mM Bis–Tris, pH 7.0.

#### Second dimension

For 2D SDS–PAGE, BN-gel slices corresponding to one lane were incubated in 2× SDS sample buffer (12.5 mM Tris, 4% SDS, 20% glycerol, 0.02% bromophenol blue, pH 6.8) for 10 min at RT. Then, the slices were boiled for 20 s in a microwave oven and incubated in hot SDS sample buffer for another 15 min at RT. The BN-PAGE gel slices were loaded in the large well over the stacking gel of the 9% SDS–PAGE gel while avoiding air bubbles. After fractionation, the gel was transferred onto PVDF (0.45-μm pore, Immobilon-P, Merck, IPVH00010) membranes and processed as for western blotting.

### IP assays

All subunits of mitochondrial CI and CIII were immunocaptured from isolated mitochondria of C2C12 cells and mouse Skm using the CI immunocapture kit (Abcam, ab109711) and CIII immunocapture kit (Abcam, ab109800), respectively, following the manufacturer’s instructions. CIII subunit UQCRC2 and COQ2 proteins were immunocaptured from isolated mitochondria of mouse Skm, heart and brain solubilized with 1% *n*-dodecyl-β-d-maltoside. Protein (1–3 mg) was incubated with 12 μg of the indicated antibody bound to EZview Red Protein G Affinity Gel (Sigma-Aldrich) at 4 °C overnight. The beads were washed twice before proteins were eluted and fractionated on SDS–PAGE.

### TMT quantitative proteomics

TMT sixplex isobaric mass tagging analysis was carried out in the CBMSO Proteomics Unit (ProteoRed, PRB3-ISCIII and UAM University, Spain)^43^.

#### In-gel digestion

Protein extracts were fractionated in an SDS–PAGE gel (0.75-mm-thick, 4% stacking and 10% resolving). Protein bands were visualized by Coomassie staining, cut into cubes (2 mm × 2 mm), destained in acetonitrile/water (ACN/H_2_O, 1:1), reduced and alkylated (disulfide bonds from cysteinyl residues were reduced with 10 mM DTT for 1 h at 56 °C, and then thiol groups were alkylated with 10 mM ACN for 30 min at RT) and digested in situ with sequencing-grade trypsin. Gel pieces were dried in a SpeedVac and reswollen in 100 mM Tris–HCl, pH 8, 10 mM CaCl_2_ with 60 ng µl^−1^ trypsin at a 5:1 protein/enzyme (w/w) ratio. Samples were desalted onto OMIX Pipette tips C18 until MS analysis.

#### TMT labelling

The resultant peptide mixture from the tryptic digest (60 µg) of desalted proteins was labelled using chemicals from the TMT sixplex Isobaric Mass Tagging Kit (Thermo Fisher Scientific) as described by the manufacturer.

#### Fractionation

The sample was then fractionated using the Pierce High pH Reversed-Phase Peptide Fractionation Kit (Thermo Fisher Scientific).

#### Quantitative analysis by reversed-phase LC–MS/MS

The fractions were resuspended in 10 µl of 0.1% formic acid and analysed in an EASY-nLC II system coupled to an ion-trap LTQ Orbitrap Velos Pro hybrid mass spectrometer (Thermo Scientific). The peptides were concentrated by reversed-phase chromatography using a 0.1 mm × 20 mm C18 reversed-phase precolumn (Thermo Scientific) and then separated using a 0.075 mm×250 mm C18 reversed-phase column (Thermo Scientific) operating at 0.3 μl min^−1^. Peptides were eluted using a 90-min dual gradient. The gradient profile was set as follows: 5–25% solvent B for 68 min, 25–40% solvent B for 22 min, 40–100% solvent B for 2 min and 100% solvent B for 18 min (solvent A: 0.1% formic acid in water, solvent B: 0.1% formic acid, 80% ACN in water). ESI ionization was done using a Nano-bore emitters Stainless Steel inner diameter 30-μm (Proxeon) interface at 2.1-kV spray voltage with an S-lens of 60%. The instrument method consisted of a data-dependent top 20 experiment with an Orbitrap MS1 scan at a resolution (*m*/Δ*m*) of 30,000 followed by 20 high-energy collision dissociation (HCD) MS/MS scans performed in the Orbitrap at 7,500 (Δ*m*/*m*) resolution. The minimum MS signal for triggering MS/MS was set to 500. The lock mass option was enabled for both MS and MS/MS mode, and the polydimethyl cyclosiloxane ions (protonated (Si(CH_3_)_2_O)_6_, 445.120025 *m*/*z* (mass/charge ratio)) were used for internal recalibration of the mass spectra. Peptides were detected in survey scans from 400 to 1,600 amu (1 μscan) using an isolation width of 1.3 u (in mass-to-charge ratio units), normalized collision energy of 40% for HCD fragmentation, and dynamic exclusion applied for 60-s periods. Charge-state screening was enabled to reject unassigned and singly charged protonated ions.

#### Quantitative data analysis

Peptide identification from raw data was carried out using the PEAKS Studio Xpro search engine (Bioinformatics Solutions). Database search was performed against the UniProt *Mus musculus* FASTA database. The search engine was configured to match potential peptide candidates with a mass-error tolerance of 10 ppm and a fragment-ion tolerance of 0.025 Da, allowing for up to two missed tryptic cleavage sites, considering fixed carbamidomethyl modification of cysteine and variable oxidation of methionine and acetylation of protein N-termini. The false discovery rate was set at 1%, and only proteins identified with at least one unique peptide were allowed. Peptide signal abundances were extracted and normalized to obtain the signal abundances at the protein level.

Quantitation of TMT-labelled peptides was performed with PEAKS Studio Xpro, with ‘Reporter Ion Quantification iTRAQ/TMT’ selected under the ‘Quantifications’ options. We used the auto-normalization mode, which calculates a global ratio from the total intensity of all labels in all quantifiable peptides. The −10LgP, Quality and Reporter Ion Intensity (1e4) parameters were used for the spectrum filter, and significance (PEAKSQ method) was used for peptide and protein abundance calculation.

After normalization and filtering steps, proteomic data were analysed by Gene Set Enrichment Analysis v4.1.0 (http://www.gsea-msigdb.org/gsea/index.jsp) and visualized by Cytoscape v3.6.1 and SRplot free software.

### IP coupled with LC–MS analysis

IP coupled with LC–MS analysis was performed at the CNB Proteomics Unit, Madrid, Spain. IP samples were digested with trypsin on S-Trap columns (Protifi). Tryptic peptides were dried and resuspended at 120 ng μl^−1^ according to Qubit quantification (Thermo Fisher Scientific). Five microlitres of each sample (equivalent to 600 ng) was loaded online on a C18 PepMap 300-µm inner diameter, 0.3 mm × 5 mm trapping column (5 µm, 100 Å, Thermo Fisher Scientific) and analysed by LC–ESI–MS/MS using a Thermo Ultimate 3000 RSLCnano UPLC coupled to a Thermo Orbitrap Exploris OE240 mass spectrometer. Peptides were separated on a 15 cm × 75 µm reversed-phase C18 column in a 60-min-long gradient at a 300 nl min^−1^ flow rate. The LC system was coupled to the mass spectrometer through an EASY-Spray source. Data acquisition was performed using a data-dependent method in full-scan positive mode, scanning from 375 to 1,250 *m*/*z*. Survey scans were acquired at a resolution of 60,000 at 200 *m*/*z*, with a normalized automatic gain control (AGC) target of 300% and the maximum injection time set to auto. The top 20 most intense ions from each MS1 scan were selected and fragmented through HCD. The resolution for HCD spectra was set to 30,000 at 200 *m*/*z*, with an AGC target of 100% and the maximum ion injection time set to auto. Isolation of precursors was performed with a window of 0.7 *m*/*z*, dynamic exclusion of 5 s and HCD collision energy of 30. Precursor ions with single, unassigned, or six and higher charge states from fragmentation selection were excluded.

#### Proteomics data analysis and sequence search

Raw files were converted to mgf files, and MS/MS spectra were searched using Proteome Discoverer 2.5 (Thermo Fisher Scientific) and Mascot v2.7 as a search engine against a composite target database built from the *Mus musculus* reference proteome. The search engine was configured as above.

### BN-PAGE proteomics by DiS MS (BN-Dis)

BN-Dis was carried out in the CNB and CNIC (Centro Nacional de Investigaciones Cardiovasculares) Protein Facilities (Madrid, Spain). DiS is a data-independent acquisition method that covers all possible fragmentations of precursors in the 400–1,100 *m*/*z* range in two LC–MS runs and uses narrow MS/MS windows of 2 *m*/*z*, typical of data-dependent acquisition methods. The BN-DiS workflow generated a multidimensional, high-resolution, time-fragment mass map for all possible precursors in each BN-PAGE fraction^21^. In brief, BN-PAGE gels were divided into 12 or 26 slices, taking as reference some discrete Coomassie-stained bands. All slices were cut into cubes (2 mm × 2 mm), reduced with 10 mM dithiothreitol, alkylated with 55 mM iodoacetamide and subjected to a standard overnight in-gel digestion at 37 °C with 3 μg of sequencing-grade trypsin in 100 mM ammonium bicarbonate (pH 7.8). After desalting with C18 Omix cartridges, the resulting tryptic peptide mixtures were injected onto a C18 reversed-phase nanocolumn (75-μm inner diameter and 50-cm length) using an EASY-nLC 1000 LC system and analysed in a continuous gradient consisting of 8–31% B for 130 min and 50–90% B for 1 min (B = 0.5% formic acid in ACN). Peptides were eluted from the reversed-phase nanocolumn at a flow rate of ∼200 nl min^−1^ to an emitter nanospray needle for real-time ionization and peptide fragmentation in either a Q-Exactive or Q-Exactive HF mass spectrometer. Each sample was analysed in two chromatographic runs covering different mass ranges (from 400 to 750 Da and 750 to 1,100 Da, respectively). The DiS cycle consisted of 175 sequential HCD MS/MS fragmentation events with 2-Da windows that covered the whole 350-Da range. All materials were from Thermo Fisher Scientific, as detailed in ref. ^21^. Alternatively, Skm BN-PAGE gel slices were digested with trypsin using an Opentrons OT-2 robot following standard protocols^71^, and LC–MS analysis was performed as above, although with a shorter gradient (40 min).

### Determination of stoichiometry between complexes in the metabolon

The approximate stoichiometry between CIII and ETF complexes was calculated as in ref. ^21^. In brief, the number of peptide spectrum matches (PSMs) for each protein, as calculated by proteomics, was shown to be approximately proportional to the number of tryptic peptides detectable by MS (NOP). The individual MS response of each protein was normalized to generate an estimation of the effective NOP. By plotting the PSMs of the proteins against their effective NOP, we estimated from the slopes the molar stoichiometry of the proteins (ETF and CIII complexes) within the metabolon in each BN-PAGE slice or IP sample.

### Structural studies

Structural studies were performed using publicly available crystal structures of mitochondrial CIII (5xte) and ETFDH (2GMH) in PDB format using PyMOL v.2.5. For docking and molecular dynamic studies, ClusPro (https://cluspro.bu.edu/), InterProSurf Protein–Protein Interaction Server (https://curie.utmb.edu/prosurf.html) and Missense3D (http://missense3d.bc.ic.ac.uk/) software were used.

### Mathematical studies

Numerical simulations were performed using Julia language from DifferentialEquations.jl. The ordinary differential equations (ODEs) of this phenomenological model describe the dynamics of each of the reactants of the equations described in Extended Data Fig. 9:

1. *r*_1_ = *k*_1_ × *x*_1_ × *x*_2_ × *x*_3_
2. *r*_2_ = *k*_2_ × *x*_3_ × *x*_4_ × *x*_7_ × *x*_8_
3. *r*_3_ = *k*_1_ × *x*_1_ × *x*_2_ × *x*_3_
4. *r*_4_ = *k*_2_ × *x*_3_ × *x*_4_ × *x*_7_ × *x*_8_
5. *r*_5_ = *k*_3_ × *x*_3_ × *x*_9_ × *x*_12_
6. *r*_6_ = *k*_4_ × *x*_3_ × *x*_10_ × *x*_13_ × *x*_14_
7. *r*_7_ = *k*_5_ × *x*_3_ × *x*_12_ × *x*_13_

ODEs were obtained from the chemical interactions by using a generalized formulation of the mass action law. The equations are as follows:

(8) d*x*_1_/d*t* = −*k*_1_ × *x*_1_ × *x*_2_ × *x*_3_ − *k*_1_ × *x*_1_ × *x*_2_ × *x*_3_ + *k*_4_ × *x*_3_ × *x*_10_ × *x*_13_ × *x*_14_ + *k*_5_ × *x*_3_ × *x*_12_ × *x*_13_
(9) d*x*_2_/d*t* = −*k*_1_ × *x*_1_ × *x*_2_ × *x*_3_ − *k*_1_ × *x*_1_ × *x*_2_ × *x*_3_
(10) d*x*_3_/d*t* = −*k*_1_ × *x*_1_ × *x*_2_ × *x*_3_ − *k*_2_ × *x*_3_ × *x*_4_ × *x*_7_ × *x*_8_ − *k*_1_ × *x*_1_ × *x*_2_ × *x*_3_ − *k*_2_ × *x*_3_ × *x*_4_ × *x*_7_ × *x*_8_ − *k*_3_ × *x*_3_ × *x*_9_ × *x*_12_ − *k*_4_ × *x*_3_ × *x*_10_ × *x*_13_ × *x*_14_ − *k*_5_ × *x*_3_ × *x*_12_ × *x*_13_
(11) d*x*_4_/d*t* = *k*_1_ × *x*_1_ × *x*_2_ × *x*_3_ − *k*_2_ × *x*_3_ × *x*_4_ × *x*_7_ × *x*_8_ + *k*_1_ × *x*_1_ × *x*_2_ × *x*_3_ − *k*_2_ × *x*_3_ × *x*_4_ × *x*_7_ × *x*_8_
(12) d*x*_5_/d*t* = *k*_1_ × *x*_1_ × *x*_2_ × *x*_3_ + *k*_1_ × *x*_1_ × *x*_2_ × *x*_3_
(13) d*x*_6_/d*t* = *k*_1_ × *x*_1_ × *x*_2_ × *x*_3_ + *k*_2_ × *x*_3_ × *x*_4_ × *x*_7_ × *x*_8_ + *k*_1_ × *x*_1_ × *x*_2_ × *x*_3_ + *k*_2_ × *x*_3_ × *x*_4_ × *x*_7_ × *x*_8_
(14) d*x*_7_/d*t* = *k*_4_ × *x*_3_ × *x*_10_ × *x*_13_ × *x*_14_ + *k*_5_ × *x*_3_ × *x*_12_ × *x*_13_
(15) d*x*_8_/d*t* = −*k*_2_ × *x*_3_ × *x*_4_ × *x*_7_ × *x*_8_ − *k*_2_ × *x*_3_ × *x*_4_ × *x*_7_ × *x*_8_ + *k*_3_ × *x*_3_ × *x*_9_ × *x*_12_ + *k*_4_ × *x*_3_ × *x*_10_ × *x*_13_ × *x*_14_
(16) d*x*_9_/d*t* = −*k*_3_ × *x*_3_ × *x*_9_ × *x*_12_
(17) d*x*_10_/d*t* = *k*_3_ × *x*_3_ × *x*_9_ × *x*_12_ − *k*_4_ × *x*_3_ × *x*_10_ × *x*_13_ × *x*_14_
(18) d*x*_11_/d*t* = *k*_4_ × *x*_3_ × *x*_10_ × *x*_13_ × *x*_14_
(19) d*x*_12_/d*t* = *k*_2_ × *x*_3_ × *x*_4_ × *x*_7_ × *x*_8_ − *k*_3_ × *x*_3_ × *x*_9_ × *x*_12_ − *k*_5_ × *x*_3_ × *x*_12_ × *x*_13_
(20) d*x*_13_/d*t* = *k*_2_ × *x*_3_ × *x*_4_ × *x*_7_ × *x*_8_ − *k*_4_ × *x*_3_ × *x*_10_ × *x*_13_ × *x*_14_ − *k*_5_ × *x*_3_ × *x*_12_ × *x*_13_
(21) d*x*_14_/d*t* = *k*_3_ × *x*_3_ × *x*_9_ × *x*_12_ − *k*_4_ × *x*_3_ × *x*_10_ × *x*_13_ × *x*_14_

The initial conditions used were as follows: *x*_1_ = QH_2_ = 0.6; *x*_2_ = Cyt *c*_ox_ = 1.1; *x*_3_ = e^−^ = 4.6; *x*_4_ = QH•_CIII–Qo_ = 0; *x*_5_ =Cyt *c*_red_ = 0; *x*_6_ = H^+^_P_ = 0; *x*_7_ = Q = 0.5; *x*_8_ = H^+^_N_ = 0.5; *x*_9_ = ETFDH_FADH2_ = 0, 0.5, 1; *x*_10_ = ETFDH_FADH_ = 0; *x*_11_ = ETFDH_FAD_ = 0; *x*_12_ = QH•_CIII–Qi–1step_ = 0; *x*_13_ = QH•_CIII–Qi–2step_ = 0; *x*_14_ = QH•_ETFDH_ = 0.

Kinetic parameter values for the interactions were tuned to ensure changes in the variables during the simulation and to obtain a response that qualitatively resembles the experiments. The results obtained are robust to moderate changes in the values of the kinetic constants chosen. The values of the kinetic rate constants for each reaction are as follows: *k*_1_ = 5; *k*_2_ = 5; *k*_3_ = 5; *k*_4_ = 5; *k*_5_ = 20; *k*_6_ = 21; *k*_7_ = 21.

### Statistics and reproducibility

In all figures, each experiment was repeated at least three independent times, with similar results. Data are presented as the mean ± s.e.m. of the indicated *n* and analysed using SPSS 17.0 and GraphPad Prism 9 software packages. Two-tailed Student’s *t* test was used for comparing two groups, and ordinary one-way ANOVA followed by Tukey’s, Dunnett’s or Šidák’s comparison tests were used for comparing multiple groups. Two-way ANOVA followed by Tukey’s or Šidák’s comparison tests were used to compare multiple variables. *P* < 0.05 was considered statistically significant. Exact *P* values for each graph are reported in Supplementary Table 3. The *n* used in each statistical test and the individual statistical analysis performed are indicated in the figure legends.

### Reporting summary

Further information on research design is available in the Nature Portfolio Reporting Summary linked to this article.

### Supplementary information

Supplementary Information Supplementary Tables 1–3 and Figs. 1 and 2.

Reporting Summary

### Source data

Source Data Fig. 1 Statistical source data.

Source Data Fig. 2 Statistical source data.

Source Data Fig. 3 Statistical source data.

Source Data Fig. 4 Statistical source data.

Source Data Figs. 1–4 and Extended Data Figs. 1, 2, 6 and 8 Unprocessed western blots.

Source Data Extended Data Fig. 1 Statistical source data.

Source Data Extended Data Fig. 2 Statistical source data.

Source Data Extended Data Fig. 4 Statistical source data.

Source Data Extended Data Fig. 5 Statistical source data.

Source Data Extended Data Fig. 6 Statistical source data.

Source Data Extended Data Fig. 7 Statistical source data.

Source Data Extended Data Fig. 8 Statistical source data.

## Data Availability

Data supporting this study are included within the article and supporting materials or have been deposited in public repositories. Accession for proteomics: ProteomeXchange through the PRIDE database (project name: C2C12 myoblast wt versus ETFDH-ko TMT). Project accession: PXD041825. Project: 10.6019/PXD041825. Project name: ETFDH, CIII and COQ2 common interactome. Project accession: PXD045351. Project: 10.6019/PXD045351. Project name: ETFDH comigrates with CIII in BN-PAGE. Project accession: PXD045352. Project: 10.6019/PXD045352. Project name: Antimycin A-treated versus ETFDH-ko myoblasts TMT. Project accession: PXD045588. Project: 10.6019/PXD045588. J.A. Enriquez kindly shared proteomics data available in ref. ^21^. PDB ID for ETFDH: 2GMH. All materials are available from the corresponding author on reasonable request or material transfer agreement. Source data are provided with this paper.
