# Supplementary material for: An ETFDH-driven metabolon supports OXPHOS efficiency in skeletal muscle by regulating coenzyme Q homeostasis
Source: Nat Metab. 2024 Jan 19;6(2):209–25. doi: 10.1038/s42255-023-00956-y (PMC10896730; doi:10.1038/s42255-023-00956-y)
Supplement: Supplementary file 1 — Supplementary Tables 1–3 and Figs. 1 and 2. [file 42255_2023_956_MOESM1_ESM.pdf]

nature metabolism

Letter

https://doi.org/10.1038/s42255-023-00956-y

An ETFDH-driven metabolon supports OXPHOS efficiency in skeletal muscle by regulating coenzyme Q homeostasis

In the format provided by the
authors and unedited

Inventory

- Supplementary Table 1
- Legend to Supplementary Table 1

- Supplementary Table 2
- Legend to Supplementary Table 2

- Supplementary Table 3
- Legend to Supplementary Table 3

- Supplementary Figure 1
- Legend to Supplementary Figure 1

- Supplementary Figure 2
- Legend to Supplementary Figure 2

Patient	Gene	Variant 1	Variant 2	Urine metabolites (mmol/mol creatinine) values (reference range)	Plasma Acylcarnitine (µmol/L) values (reference range)	Others
1	*ETFDH*	c.1351G>C (p.Val451Leu)	c.1852T>C (p.*618Gln)	lactic 223 (1-113)<br>ethylmalonic 29 (0-18)<br>glutaric 91 (1-10)<br>2-OH-glutaric 95 (3-48)<br>fumaric 43 (0-0.17)<br>proline 94.4 (22.8±20.0)	C4 0.86 (0.09-0.48)<br>C6 0.35 (0.00-0.13)<br>C8 0.62 (0.01-0.25<br>C10 1.48 (0.04-0.41)<br>C12 1.48 (0.02-0.11)<br>C14 2.62 (0.01-0.07)<br>C16 4.09 (0.04-0.28)<br>C18 1.75 (0.00-0.13)<br>C10:1 0.29 (0.02-0.24)<br>C12:1 0.58 (0.01-0.13)<br>C16:1 3.90 (0.00-0.07)<br>Increases of dicarboxylic acyl-carnitines and 3-OH (C14-C18)	
2	*ETFDH*	c.34+5G>C	c.244T>C (p.Ser82Pro)	lactic 3218 (1-121)<br>2-OH-butyric 15 (1-4)<br>3-OH-butyric 3132 (0-11)<br>acetoacetate 752 (0-2)<br>glutaric 980 (0-5)<br>2-OH-glutaric 290 (0-62)<br>4-OH-phenyl-lact 416 (0-17)<br>4-OH-phenyl-pyr 206 (0-74)<br>succinic 193 (3-83)<br>fumaric 144 (0-42)<br>malic 115 (0-35)	C4 1.76 (0.00-0.48)<br>C5 1.87 (0.03-0.35)<br>C6 1.78 (0.01-0.13)<br>C8 2.81 (0.01-0.26)<br>C10 3.18 (0.00-0.35)<br>C12:1 0.67 (0.00-0.14)<br>C12 1.63 (0.00-0.17)<br>C14:1 0.92 (0.00-0.17)<br>C14 1.22 (0.01-0.09)<br>C16:1 0.59 (0.00-0.08)<br>C16 2.68 (0,02-0.30)<br>C18:1 0.82 (0.01-0.26)<br>C18 0.46 (0.01-0.16)<br>Increases of dicarboxylic acyl carnitines and 3-OH (C14-C18)	
3	*ETFDH*	c.79C>T (p.Pro27Ser)	c.831delG	lactic 172 (1-113)<br>ethylmalonic 72 (0-18)<br>isobutyryl glycine 6 (0-3)<br>isovaleryl glycine 32 (1-4)<br>hexanoylglycine 7 (ND)<br>3-OH-glutaric 29 (1-12)<br>glycine 372,1(208,6±66)<br>alanine 478,3 (283±88)	C5 0.39 (0.03-0.20)<br>C6 0.27 (0.00-0.13)<br>C12 0.18 (0.02-0.11)<br>C14:1 0.39 (0.02-0.17)<br>C14 0.16 (0.01-0.07)<br>C16:2 0.12 (0.00-0.02)<br>C16:1 0.34 (0.00-0.07)<br>C18:1 0.32 (0.04-0.19)	
4	*BCS1L*			lactic 692 (1-121)<br>3-OH-butyric 833 (0-11)<br>acetoacetic 33 (0-2)<br>glutaric 6 (0-5)<br>succinic 131 (3-83)<br>fumaric 154 (0-42)<br>malic 328 (0-35)<br>proline 1211 (23±20)<br>citrulline 99 (4±5)<br>threonine 507 (63±47)<br>serine 623 (131±60)	C6 0.17 (0.03-0.13)<br>C10 0.46 (0.04-0.41)<br>C12 0.29 (0.06-0.17)<br>C16 1.27 (0.02-0.301)<br>C4-OH 0.14 (0.01-0.06)<br>C14:1 0.23 (0.00-0.17)<br>C18:1 0.47 (0.01-0.26)<br>(C16+C18:1)/C2= 0.262 (0.004-0.069)	
5	*BCS1L*	c.-147A>G	c.166C>T (p.Arg56*)	citrulline 108 (23-58)		Oxidation rates* of [1-^{14}C]-octanoate; [1-^{14}C]-butyrate and [9,10-^{3}H] -palmitoyl-carnitine measured in fibroblast were 0.16 nmol/h. mg protein (controls range 0.4-0.9); 0.38 nmol/h. mg protein (controls range 0.5-1.4) and 4.17 nmol/h. mg protein (controls range 5.1-8.7) respectively.

Table 1. Metabolomic analysis on urine and blood of patients presenting ETFDH mutations or CIII dysfunctions. *ETFDH* gene: NM_004453.4; *BCS1L* gene: NM_001079866.2; Biochemical data obtained at diagnosis. In parenthesis, reference values. 4-OH-phenyl-lact (4-OH-phenyl-lactic). 4-OH-phenyl-pyr (4-OH-phenyl-pyruvic).

Reagent or Resource	Source	RRID	Reference	Molecular Weight (kDa)	Origin	Use
**Antibodies**						
Anti-ACC	Cell Signaling Technology Inc	AB_2219400	3662	265	Rabbit/Polyclonal	WB (1:1000)
Anti-ACLY	Abcam	AB_722533	ab40793	140	Mouse/Monoclonal	WB (1:1000)
Anti-CDK1	Cell Signaling Technology Inc	AB_2716331	77055	34	Rabbit/Polyclonal	WB (1:1000)
Anti-COQ2	Santa Cruz	-	sc-517107	40	Mouse/Monoclonal	WB (1:1000), IP, PLA (1:100)
Anti-COXIV	Invitrogen	AB_1501840	459600	24	Mouse/Monoclonal	WB/BN (1:1000)
Anti-DRP1	BD Transduction Laboratories	AB_398423	611112	81	Mouse/Monoclonal	WB (1:1000)
Anti-ETFA	Abcam	AB_10865517	ab110316	35	Mouse/Monoclonal	WB (1:1000), IP
Anti-ETFB	Abcam	AB_10711450	ab104944	27	Goat/Polyclonal	WB/BN (1:1000)
Anti-ETFDH	Thermo Fisher	AB_10988351	PA5-19340	67	Rabbit/Polyclonal	WB/BN (1:1000), PLA (1:100)
Anti-FASN	BD Transduction Laboratories	AB_398275	610962	270	Mouse/Monoclonal	WB (1:1000)
Anti-H2AX	Abcam	AB_297813	ab11174	17	Rabbit/Polyclonal	WB (1:1000)
Anti-HADHA	Abcam	AB_2263836	ab54477	83	Rabbit/Polyclonal	WB (1:1000)
Anti-IF1	Homemade	-	Sánchez-Cenizo *et al.*, 2010	12	Mouse/Monoclonal	WB (1:1000)
Anti-MFN1	Abcam	AB_2142624	ab57602	85	Mouse/Monoclonal	WB (1:1000)
Anti-MFN2	Abcam	AB_2142629	ab56889	85	Mouse/Monoclonal	WB (1:1000)
Anti-Mouse IgG (H+L), HRP Conjugate	Nordic Immunology	-	RAM/IgG(H+L)/PO	-		WB (1:5000)

Anti-NDUFA9	Abcam	AB_301431	ab14713	42	Mouse/ Monoclonal	WB/BN (1:1000), IP
Anti-OPA1	BD Transduction Laboratories	AB_399888	612606	111	Mouse/ Monoclonal	WB (1:1000)
Anti-p53	Abcam	AB_303198	Pab 240	53	Rabbit/Polyclonal	WB (1:1000)
Anti-pACC	Cell Signaling Technology Inc	AB_330337	3661	265	Rabbit/Polyclonal	WB (Ser79 phosphorylation) (1:1000)
Anti-pCDK1	Cell Signaling Technology Inc	AB_560953	4539	34	Rabbit/Polyclonal	WB (Tyr15 phosphorylation) (1:1000)
Anti-pSMAD3	Abcam	AB_882596	ab52903	48	Mouse/ Monoclonal	WB (1:1000)
Anti-Rabbit IgG (H+L), HRP Conjugate	Nordic Immunology	-	GAR/IgG(H+L)/PO	-		WB (1:5000)
Anti-SDHA	Abcam	AB_301433	ab14715	72	Mouse/ Monoclonal	WB (1:1000), IP
Anti-SDHB	Invitrogen	AB_2532233	459230	32	Mouse/ Monoclonal	WB/BN (1:1000)
Anti-tubulin	Sigma-Aldrich	AB_477593	T9026	50	Mouse/ Monoclonal	WB (1:3000)
Anti-UQCRC2	Abcam	AB_2213640	ab14745	48	Mouse/ Monoclonal	WB/BN (1:1000), IP, PLA (1:200)
Anti-UQCRC2	Proteintech	AB_2241442	14742-1-AP	48	Rabbit/Polyclonal	PLA (1:100)
Anti-VDAC	Abcam	AB_2214787	ab15895	31	Mouse/ Monoclonal	WB/BN (1:1000)
Anti-β-actin	Sigma-Aldrich	AB_476697	A2228	47	Mouse/ Monoclonal	WB (1:1000)
Anti-β-F1ATPase	Homemade	-	Cuezva *et al.*, 2002	52	Rabbit/Polyclonal	WB/BN (1:3000)
Donkey anti-goat	Thermo Fisher	-	A-21447	-	Donkey	IF (1:3000)
Donkey anti-mouse	Sigma-Aldrich	-	SAB4600176-250UL	-	Donkey	IF (1:3000)
Goat anti-rabbit	Sigma-Aldrich	-	SAB4600185-250UL	-	Goat/Polyclonal	IF (1:3000)
**Chemicals, Peptides and Recombinant Proteins**						

[14C(U)]-L-Isoleucine	Perkin-Elmer	-	NEC278E050UC			
[14C(U)]-L-Leucine	Perkin-Elmer	-	NEC279E050UC			
[9,10-3H(N)]-Palmitic Acid	Perkin-Elmer	-	NET043001MC			
1 kb Plus and Ladder	Thermo Fisher		10787018			
2-isopropanol	Merk KGaA	-	K48640034			
4-Clorobenzoic acid (4-CBA)	Sigma-Aldrich	-	135585			
6-aminohexanoic acid	Sigma-Aldrich	-	7260			
ABI Prism 7900HT sequence detection system	Thermo Fisher Scientific	-	4317596			
Acetic acid	Sigma-Aldrich	-	71251			
Antimycin A	Sigma-Aldrich	-	A8474			
Bio-Rad Protein Assay	Bio-Rad	-	5000006			
Bis-Tris	Sigma-Aldrich	-	T0377			
Bovine Serum Albumin (BSA)	Nzytech	-	MB04602			
Bromophenol Blue	Merck	-	108122			
Calcein	Invitrogen	-	C1430			
Carboxin	Sigma-Aldrich	-	45371			
Carnitine	Sigma-Aldrich	-	C0283			
Chloroform	Merck		67-66-3			
cOmpleteTM Mini EDTA-free Protease Inhibitor Cocktail	Sigma-Aldrich	-	11836170001			
DCPIP	Sigma-Aldrich	-	1.03028			
DeStreak Rehydration Solution	GE Healthcare		17600319			
Digitonin	Sigma-Aldrich	-	D5628			
Dithionite	Sigma-Aldrich	-	1.06505			
DL-Dithiothreitol (DTT)	Sigma-Aldrich	-	D0632			

Doxycyline	Sigma-Aldrich	-	D9891			
EDTA	Sigma-Aldrich	-	ED2P			
EtOH	Merck KGaA	-	64-17-5			
Etomoxir	Sigma-Aldrich	-	E1905			
EZ View Red Protein G Affinity Gel	Sigma-Aldrich	-	E3403			
Fast SYBERMasterMix	Thermo Fisher	-	4385616			
FCCP	Sigma-Aldrich	-	C2920			
Fetal Bovine Serum (FBS)	Sigma-Aldrich	-	F7524			
FuGene™ Transfection Reagent	Promega	-	E2311			
Gel-Red® Nucleic Acid Gel Stain	Biotium	-	41003			
Glucose	Merck KGaA	-	K37462374			
Glutamate	Sigma-Aldrich	-	49621			
Glycerol	Sigma-Aldrich	-	G5516			
Glycine	VWR	-	101196X			
H2DCFDA (H2-DCF, DCF)	Invitrogen		D399			
Idebenone	Selleckchem	-	S2605			
Insulin	Sigma-Aldrich	-	I6634			
Iodoacetamide (IAM)	Sigma-Aldrich	-	I6125			
IPG buffer	GE Healthcare		17600178			
KCl	Merk	-	104936			
KCN	Sigma-Aldrich		207810-25G			
Lactate Dehydrogenase	Roche	-	LLDH-RO			
Lipofectamine 3000 Transfection Reagent	Thermo Fisher Scientific	-	L3000015			
Malonate	Sigma-Aldrich	-	M1296			
Metanol (MetOH)	Sigma-Aldrich	-	34860			
$MgCl_2$	Sigma-Aldrich	-	M8266			

MitoQ	Antipodean Pharmaceutical Inc.	-	Provided by Michael P. Murphy			
MitoSOX Red Mitochondrial superoxide indicator	Invitrogen	-	M36008			
Mowiol	Merck KGaA	-	475904-100GM			
Myxothiazol	Sigma-Aldrich	-	T5580			
Native PAGE Novex 3-12% Bis-Tris Protein Gel	Life Technologies		BN1001BOX			
Non-fatty dried milk	Central lechera Asturiana	-				
Novex ECL HRP Chemiluminiscent reagent	Invitrogen	-	WP20005			
Oleic Acid	Sigma-Aldrich	-	O1008			
Oligomycin	Sigma-Aldrich	-	O4876			
Paraformaldehyde	Santa Cruz Biotechnology	-	30525-89-4			
Phenazine methosulfate	Sigma-Aldrich		P-9625			
Phosphatase inhibitor cocktail 2	Sigma-Aldrich	-	P5726			
PI/RNase Staining Buffer (Cell-cycle buffer)	BD Biosciences	-	550825			
Ponceau Red	Sigma-Aldrich	-	P7170			
Rotenone	Sigma-Aldrich	-	R8875			
Serva Blue G dye	Serva	-	35050			
Sodium Palmitate	Sigma-Aldrich	-	P9767			
Sodium Succinate	Sigma-Aldrich	-	S7501			
Sucrose	Sigma-Aldrich	-	84100			
Tetramethylrhodamine methyl ester (TMRM)	Thermo Fisher Scientific		T668			

Tricine	Sigma-Aldrich	-	B9754			
Tris pH 8.3	Sigma-Aldrich	-	T1503			
TritonX-100	Merck KGaA	-	9030-19-5			
Trizol	Invitrogen	-	15596026			
Tween 20	EMD Millipore Corp.	-	817072			
Xylene	VWR	-	1330-20-7			
**Critical Commercial Assays**						
ATP Bioluminescence Assay Kit CLS II	Roche	-	11699695001			
CloneAmp HiFi PCR Premix	Takara	-	639298			
Complex I Immunocapture Kit	Abcam	-	ab109711			
Complex III Immunocapture Kit	Abcam	-	ab109800			
Duolink® In Situ Green Starter Kit Mouse/Rabbit	Sigma-Aldrich	-	DUO94102			
FAD Assay Kit	Abcam	-	ab204710			
High-Capacity cDNA Reverse Transcription Kit	Thermo Fisher	-	4368814			
KAPA Mouse Genotyping Kit	Kapa Biosystems	-	KK7302			
Mitochondrial Complex III Activity Assay Kit	Sigma-Aldrich	-	MAK360			
NAD/NADH Quantification Kit	Sigma-Aldrich	-	MAK037			
NADP/NADPH Quantification Kit	Sigma-Aldrich	-	MAK038			
Plasmid MAXI Kit	Qiagen	-	12123			
SkGM™-2 Skeletal Muscle Cell Growth	Lonza	-	CC-3245			

Medium-2 BulletKit™						
Wizard® plus SV Minipreps DNA Purification System	Promega	-	A1330			
Wizard® SV Gel-PCR Clean-Up System	Promega	-	A9281			
**Plasmids**						
pcDNA3.1(+)		-				
pCMV_SPORT6		-				
pGEM-t	Promega	-				
pSpCas9(BB)-2A-GFP	Addgene	-	48138			
**Software and Algorithms**						
ClusPro 2.0	Boston University	SCR_018248				
Clustal Omega 1.2.2	EMBL-EBI	SCR_001591				
Cytoscape v3.6.1	Cytoscape Consortium	SCR_015784				
DOEUMH	J. Morales, A.M. Mayoral, 2015	-				
FlowJo software v10.6.2	BD Biosciences	SCR_008520				
Cas-Designer	CRISPR RGEN Tools	-				
GraphPad Prism9	GraphPad	SCR_002798				
GSEA v4.1.0	Broad Institute Inc.	SCR_003199				
Image J analysis software 1.53t	NIH	SCR_003070				
InterProSurf	The University of Texas Medical Branch.	-				
Julia Programming Language v1.9.3	Julia Programming	SCR_021666				

	Language					
Mascot v2.7	Matrix Science	SCR_014322				
Missense 3D	Imperial College London	-				
PAXdb v5.0	University of Zurich	SCR_018910				
PEAKS Studio X search engine 11	Bioinformatics Solutions Inc.	-				
Proteome Discoverer 2.5	Thermo Fisher	SCR_014477				
PyMOL v.2.5	Schrodinger	SCR_000305				
Seahorse_Wave_Desktop_v2.4	Agilent technologies	SCR_014526				
Sequence Detector System (SDS) v.2.4	Thermo Fisher	SCR_015806				
SPSS 17.0	SPSS Inc.	SCR_002865				
SRplot	Shanghai Jiaotong University	-				
Thermo Xcalibur	Thermo Fisher	SCR_014593				
**Other**						
Grip Strength Test	Hardvard Apparatus	-	76-1068			
Nitrocellulose membrane, Amersham Protran 0.2mm NC	GE Healthcare	-				
Poly-Prep Chromatography column	Bio-Rad	-	7311550			
PVDF membrane, Immobilon-P, 0.45uM	Merck KGaA	-	IPVH00010			
Rotarod Machine	Sánchez-González *et al.,* 2022	-				
XF24 Flux Pack, Seahorse Bioscience	Agilent Technologies	-	100867-100			

XFe96 Flux Pack, Seahorse Bioscience	Agilent Technologies	-	102416-100			

Primer sequences utilized for RT-PCR

GENE	PRIMER	SEQUENCE
Cdk1	Fw:	GGGAATTGTGTTTTGCCACT
	Rv:	GATGTCAACCGGAGTGGAGT
Cdk2	Fw:	AAGATTGGAGAGGGCACGTA
	Rv:	CATCCAGCAGCTTGACGATA
Cdk4	Fw:	CTGGTACCGAGCTCCTGAAG
	Rv:	GTCGTCTTCTGGAGGCAATC
Cdk6	Fw:	TGTTTCAGCTTCTCCGAGGT
	Rv:	GACTGGAGCAGGACTTCTGG
Cyclin a2	Fw:	CAAGACTCGACGGGTTGCTC
	Rv:	GCTGGCCTCTTCTGAGTCTC
Cyclin b1	Fw:	TGTGTGAACCAGAGGTGGAA
	Rv:	GCGTCTACGTCACTCACTGC
Cyclin d1	Fw:	AGTGCGTGCAGAAGGAGATT
	Rv:	CACAACTTCTCGGCAGTCAA
Cyclin e	Fw:	CAGACTCTCCGCAAGAAACC
	Rv:	TAATGCAAGGGCTGATTCCT
p21	Fw:	GTACTTCCTCTGCCCTGCTG
	Rv:	TCTGCGCTTGGAGTGATAGA
p27	Fw:	TTGGGTCTCAGGCAAACTCT
	Rv:	TCTGTTCTGTTGGCCCTTTT
p57	Fw:	AGGAGCAGGACGAGAATCAA
	Rv:	ACGTTTGGAGAGGGACACC
Ink4a	Fw:	GTACCCCGATTCAGGTGATG
	Rv:	TCTTGATGTCCCCGCTCTT
Ink4c	Fw:	AACTGCGCTGCAGGTTATG
	Rv:	GGGCAGGTTCCCTTCATTAT
Tgf-β	Fw:	TGCGCTTGCAGAGATTAAAA
	Rv:	AGCCCTGTATTCCGTCTCCT
Smad3	Fw:	CACAGCCACCATGAATTACG
	Rv:	GAATATTGCTCTGGGGCTCA
Etfa	Fw:	TTGGAGGTGAAGTGTCCTGC
	Rv:	AGCTACTCTGGGCAGAAGGT
Etfb	Fw:	AAAGCCGGACAAGTCTGGAG
	Rv:	CAGTTCGGATGGTCTCCTGG
Dld	Fw:	GGCGGCCCTTTACTCAGAAT
	Rv:	GGCCATCCCTTCAACACAGA
Gpd2	Fw:	AACGATGCACGGATGAACCT
	Rv:	ATCATCCATTTTGCGCACGG
Prodh	Fw:	GGGCAGCAGAGATCGGTTAT
	Rv:	CAGCAGGATGCAGGCCTATC

Dhodh	Fw:	CCTGGTGGTTAACGTGTCCA
	Rv:	ATGATCAGTCCGTCGATGCC
Myh1	Fw:	AAGACCGAAGGCGGAACTAC
	Rv:	TGACAGTGACGCAGAACAGG
Myh7	Fw:	CTCAAGCTGCTCAGCAATCTATTT
	Rv:	GGAGCGCAAGTTTGTCATAAGT
Myo g	Fw:	GTGCCCAGTGAATGCAACTC
	Rv:	GCTGTCCACGATGGACGTAA
Myo d	Fw:	CTCCAACTGCTCTGATGGCA
	Rv:	GAGATGCGCTCCACTATGCT
Myf5	Fw:	TGAGGGAACAGGTGGAGAAC
	Rv:	AGCTGGACACGGAGCTTTTA
β-actin	Fw:	AACACAGTGCTGTCTGGTGGT
	Rv:	GATCCACATCTGCTGGAAGG
Gapdh	Fw:	TGCGACTTCAACAGCAACTC
	Rv:	GGATAGGGCCTCTCTTGCTC
18s	Fw:	AACGGTCTAGACAACAAGCTG
	Rv:	AGTGGTCTTGGTGTGCTGAC
**Oligonucleotides used for clonation**		
ETFDH	Fw: (HindIII)	GCGCGCAAGCTTATGCTGGTGCCGCTAGCCAAGC
	Rv: (XhoI)	GCGCGCCTCGAGTTACATTCCATTGTAAGCAGGT
Etfdh G273E	Fw:	AGACATACCTATGGAGAATCTTTCCT
	Rv:	GGAAAGATTCTCCATAGGTATGTCT
Etfdh Y271A	Fw:	AGACATACCGCTGGAGAATCTTTCCT
	Rv:	GGAAAGATTCTCCAGCGGTATGTCT
**Oligonucleotides used for CRISPR/Cas9 mediated KOs.**		
Mouse sgRNA *Etfa*	Fw:	CACCGACTGCAGCTGGACGTCTTGG
	Rv:	AAACCCAAGACGTCCAGCTGCAGTC
Mouse sgRNA *Etfb*	Fw:	CACCGTGACCACTCCAGACTTGTC
	Rv:	AAACGACAAGTCTGGAGTGGTCAC
Mouse sgRNA *Etfdh*	Fw:	CACCGAACATCTTGGAGCACACAG
	Rv:	AAACCTGTGTGCTCCAAGATGTTC
Mouse sgRNA *Prodh*	Fw:	CACCGCGAGGACCAGGAGTCTATC
	Rv:	AAACGATAGACTCCTGGTCCTCGC
Mouse sgRNA *Dld*	Fw:	CACCGAACTGAAGAAACTCCTTGT
	Rv:	AAACACAAGGAGTTTCTTCAGTTC
Mouse sgRNA *Dhodh*	Fw:	CACCGCCTGAAACGTAGCTCGAGGA
	Rv:	AAACTCCTCGAGCTACGTTTCAGGC
Mouse sgRNA *Coq2*	Fw:	CACCGATGCTGCGCTGGGGAGGCGC
	Rv:	AAACGCGCCTCCCCAGCGCAGCATC
**Other oligonucleotides**		
M13	Fw:	GTAAAACGACGGCCAGT
	Rv:	CAGGAAACAGCTATGAC
T7 promoter		TAATACGACTCACTATAGG

CMV promoter		GTGATGCGGTTTTGGCAGTA
SP6 promoter		TACGATTTAGGTGACACTATAG
U6 promoter		ATAAGGATCCGGTCTCGCTATGAGGGCCTATTTCCCATG
**Oligonucleotides used for mouse genotyping**		
Acta1	Fw:	CTGTTCGGCCTTGAATTGAT
	Rv:	CATGTCCAGATCGAAATCGTC
Etfdh 5' arm		CTGCTGTACCTCAGATTACCA
Etfdh 3' arm		GTGTGCTCACTATGCTTCAGA
Lar3		CAACGGGTTCTTCTGTTAGTCC

Table 2. Comprehensive list of reagents and resources used.

Figure	Statistical analysis	Comparison	p-value
**Figure 1**			
c	Ordinary one-way ANOVA, Tukey's multiple comparisons test	CRL vs. ETFDH-ko	<0.0001
		CRL vs. ETFDH-ko + ETFDH	0.145
		ETFDH-ko vs. ETFDH-ko + ETFDH	<0.0001
d	Ordinary one-way ANOVA, Tukey's multiple comparisons test	CRL vs. ETFDH-ko	<0.0001
		CRL vs. ETFDH-ko + ETFDH	<0.0001
		ETFDH-ko vs. ETFDH-ko + ETFDH	<0.0001
e	2way ANOVA, Sidak's multiple comparisons test	CRL-ETFDH at 0 h	>0.9999
		CRL-ETFDH at 24 h	0.0554
		CRL-ETFDH at 36 h	<0.0001
f	2way ANOVA, Sidak's multiple comparisons test	CRL-ETFDH at 0 h	0.9998
		CRL-ETFDH at 24 h	0.6236
		CRL-ETFDH at 36 h	<0.0001
g	2way ANOVA, Sidak's multiple comparisons test	CRL-ETFDH at 0 h, 4.5 mM glucose	>0.9999
		CRL-ETFDH at 24 h, 4.5 mM glucose	0.9997
		CRL-ETFDH at 36 h, 4.5 mM glucose	0.2102
		CRL-ETFDH at 0 h, 10 mM glucose	0.9215
		CRL-ETFDH at 24 h, 10 mM glucose	>0.9999
		CRL-ETFDH at 36 h, 10 mM glucose	0.1387
h	Ordinary one-way ANOVA, Tukey's multiple comparisons test	CRL vs. ETFDH-ko	0.0232
		CRL vs. ETFDH-ko + ETFDH	0.3926
		ETFDH-ko vs. ETFDH-ko + ETFDH	0.0002
i	Ordinary one-way ANOVA, Tukey's multiple comparisons test	CRL vs. ETFDH-ko	0.0282
		CRL vs. ETFDH-ko + ETFDH	>0.9999
		ETFDH-ko vs. ETFDH-ko + ETFDH	0.06
m	Ordinary one-way ANOVA, Tukey's multiple comparisons test	CRL vs. ETFDH-ko	<0.0001
		CRL vs. ETFDH-ko + ETFDH	0.0016
		ETFDH-ko vs. ETFDH-ko + ETFDH	0.0319
**Figure 2**			
a	Two-tailed Student's t-test		<0.0001
b	2way ANOVA, Sidak's multiple comparisons test	Vehicle:CRL vs. Vehicle:ETFDH-ko	0.0005
		Vehicle:CRL vs. Antimycin A:CRL	<0.0001
		Vehicle:CRL vs. Antimycin A:ETFDH-ko	<0.0001
		Vehicle:ETFDH-ko vs. Antimycin A:CRL	0.8851

		Vehicle:ETFDH-ko vs. Antimycin A:ETFDH-ko	0.0999
		Antimycin A:CRL vs. Antimycin A:ETFDH-ko	0.4636
c	Ordinary one-way ANOVA, Tukey's multiple comparisons test	CRL vs. ETFDH-ko	<0.0001
		CRL vs. ETFDH-ko + ETFDH	0.0797
		ETFDH-ko vs. ETFDH-ko + ETFDH	<0.0001
d	Ordinary one-way ANOVA, Tukey's multiple comparisons test	CRL vs. ETFDH-ko	<0.0001
		CRL vs. ETFDH-ko + ETFDH	0.6003
		ETFDH-ko vs. ETFDH-ko + ETFDH	<0.0001
e	2way ANOVA, Tukey's multiple comparisons test	VEHICLE: CRL vs. ETFDH-ko	0.9729
		VEHICLE: CRL vs. ETFDH-ko + ETFDH	0.9849
		VEHICLE: ETFDH-ko vs. ETFDH-ko + ETFDH	0.9439
		ROTENONE: CRL vs. ETFDH-ko	0.9464
		ROTENONE: CRL vs. ETFDH-ko + ETFDH	0.9585
		ROTENONE: ETFDH-ko vs. ETFDH-ko + ETFDH	0.9999
		MALONATE: CRL vs. ETFDH-ko	0.8804
		MALONATE: CRL vs. ETFDH-ko + ETFDH	0.9698
		MALONATE: ETFDH-ko vs. ETFDH-ko + ETFDH	0.976
		ANTIMYCIN A: CRL vs. ETFDH-ko	<0.0001
		ANTIMYCIN A: CRL vs. ETFDH-ko + ETFDH	0.5629
		ANTIMYCIN A: ETFDH-ko vs. ETFDH-ko + ETFDH	<0.0001
j	Ordinary one-way ANOVA, Tukey's multiple comparisons test	CRL vs ETFDH-ko	<0.0001
		CRL vs ETFDH-ko + ETFDH	0.4293
		CRL vs ETFDH-ko + Y271A + G273E	<0.0001
		ETFDH-ko vs ETFDH-ko + ETFDH	<0.0001
		ETFDH-ko vs ETFDH-ko + Y271A + G273E	0.9896
		ETFDH-ko + ETFDH vs ETFDH-ko + Y271A + G273E	<0.0001
l	Ordinary one-way ANOVA, Tukey's multiple comparisons test	CRL vs ETFDH-ko	<0.0001
		CRL vs CRL + AOX	0.0008
		CRL vs ETFDH-ko + AOX	0.4252
		ETFDH-ko vs CRL + AOX	<0.0001
		ETFDH-ko vsETFDH-ko + AOX	<0.0001
		CRL + AOX vs ETFDH-ko + AOX	<0.0001
m		CRL vs ETFDH-ko	0.0028
		CRL vs CRL + AOX	0.8022

	Ordinary one-way ANOVA, Tukey's multiple comparisons test	CRL vs ETFDH-ko + AOX	0.0433
		ETFDH-ko vs CRL + AOX	0.0002
		ETFDH-ko vsETFDH-ko + AOX	0.7188
		CRL + AOX vs ETFDH-ko + AOX	0.0043
n	Ordinary one-way ANOVA, Tukey's multiple comparisons test	CRL vs ETFDH-ko	<0.0001
		CRL vs CRL + AOX	0.0236
		CRL vs ETFDH-ko + AOX	<0.0001
		ETFDH-ko vs CRL + AOX	0.0949
		ETFDH-ko vs ETFDH-ko + AOX	0.9325
		CRL + AOX vs ETFDH-ko + AOX	0.0284
o	Ordinary one-way ANOVA, Tukey's multiple comparisons test	CRL vs ETFDH-ko	<0.0001
		CRL vs ETFDH-ko + AOX	<0.0001
		ETFDH-ko vs ETFDH-ko + AOX	0.0142
**Figure 3**			
a	Two-tailed Student's t-test		0.0601
f	2way ANOVA, Sidak's multiple comparisons test	MYOD: Myoblast:CRL vs. Myoblast:ETFDH-ko	<0.0001
		MYOD: Myoblast:CRL vs. Myocyte:CRL	<0.0001
		MYOD: Myoblast:CRL vs. Myocyte:ETFDH-ko	<0.0001
		MYOD: Myoblast:ETFDH-ko vs. Myocyte:CRL	0.0028
		MYOD: Myoblast:ETFDH-ko vs. Myocyte:ETFDH-ko	0.0006
		MYOD: Myocyte:CRL vs. Myocyte:ETFDH-ko	0.6785
		MYF5: Myoblast:CRL vs. Myoblast:ETFDH-ko	0.8594
		MYF5: Myoblast:CRL vs. Myocyte:CRL	0.0019
		MYF5: Myoblast:CRL vs. Myocyte:ETFDH-ko	0.0001
		MYF5: Myoblast:ETFDH-ko vs. Myocyte:CRL	0.0078
		MYF5: Myoblast:ETFDH-ko vs. Myocyte:ETFDH-ko	0.0004
		MYF5: Myocyte:CRL vs. Myocyte:ETFDH-ko	0.143
		MYOG: Myoblast:CRL vs. Myoblast:ETFDH-ko	0.0004
		MYOG: Myoblast:CRL vs. Myocyte:CRL	<0.0001
		MYOG: Myoblast:CRL vs. Myocyte:ETFDH-ko	<0.0001
		MYOG: Myoblast:ETFDH-ko vs. Myocyte:CRL	<0.0001
		MYOG: Myoblast:ETFDH-ko vs. Myocyte:ETFDH-ko	<0.0001
		MYOG: Myocyte:CRL vs. Myocyte:ETFDH-ko	<0.0001

		MYHC1: Myoblast:CRL vs. Myoblast:ETFDH-ko	0.8866
		MYHC1: Myoblast:CRL vs. Myocyte:CRL	<0.0001
		MYHC1: Myoblast:CRL vs. Myocyte:ETFDH-ko	<0.0001
		MYHC1: Myoblast:ETFDH-ko vs. Myocyte:CRL	<0.0001
		MYHC1: Myoblast:ETFDH-ko vs. Myocyte:ETFDH-ko	<0.0001
		MYHC1: Myocyte:CRL vs. Myocyte:ETFDH-ko	0.0253
		MYHC7: Myoblast:CRL vs. Myoblast:ETFDH-ko	0.0006
		MYHC7: Myoblast:CRL vs. Myocyte:CRL	0.0097
		MYHC7: Myoblast:CRL vs. Myocyte:ETFDH-ko	<0.0001
		MYHC7: Myoblast:ETFDH-ko vs. Myocyte:CRL	0.2085
		MYHC7: Myoblast:ETFDH-ko vs. Myocyte:ETFDH-ko	<0.0001
		MYHC7: Myocyte:CRL vs. Myocyte:ETFDH-ko	<0.0001
j	Two-tailed Student's t-test		<0.0001
k	Ordinary one-way ANOVA, Dunnett's multiple comparisons test	Riboflavin	0.8878
		Q10H2	0.0095
		4CBA 1 mM	0.0666
		4CBA 10 mM	<0.0001
		COQ2-ko	<0.0001
l	2way ANOVA, Sidak's multiple comparisons test	Vehicle	<0.0001
		4-CBA	0.5046
**Figure 4**			
d	Two-tailed Student's t-test	Day: 40	0.7043
		Day: 60	0.5613
		Day: 70	0.2864
		Day: 80	0.7209
		Day: 90	0.2063
		Day: 100	0.6709
		Day: 110	0.1470
		Day: 120	0.6405
		Day: 130	0.1894
		Day: 140	0.0092
		Day: 170	0.0055
		Day: 180	0.0307
e	Two-tailed Student's t-test		0.0648
f	2way ANOVA, Sidak's multiple comparisons test	Fed	0.9264
		Fasted	<0.0001
g		Rpm: 8	>0.9999

	2way ANOVA, Sidak's multiple comparisons test	Rpm: 16	0.6369
		Rpm: 24	0.2181
		Rpm: 32	<0.0001
		Rpm: 40	<0.0001
k	Two-tailed Student's t-test		0.0002
n	Ordinary one-way ANOVA, Tukey's multiple comparisons test	*Etfdh+/+* vs *Etfdh+/-*	0.0976
		Etfdh+/+ vs *Etfdh-/-*	0.0004
		Etfdh+/- vs *Etfdh-/-*	0.0197
**Extended data Figure 1**			
b	Two-tailed Student's t-test	Basal	0.6443
		OSR	0.3281
		Max	0.0449
c	2way ANOVA, Sidak's multiple comparisons test	Vehicle:CRL vs. Vehicle:ETFDH-ko	<0.0001
		Vehicle:CRL vs. Carnitine:CRL	0.009
		Vehicle:CRL vs. Carnitine:ETFDH-ko	<0.0001
		Vehicle:ETFDH-ko vs. Carnitine:CRL	<0.0001
		Vehicle:ETFDH-ko vs. Carnitine:ETFDH-ko	0.8742
		Carnitine:CRL vs. Carnitine:ETFDH-ko	<0.0001
d	Two-tailed Student's t-test		<0.0001
e	Two-tailed Student's t-test	Basal	<0.0001
		OSR	<0.0001
		Max	0.0003
f	Ordinary one-way ANOVA, Tukey's multiple comparisons test	CRL vs ETFDH-ko	0.0014
		CRL vs ETFDH-ko + ETFDH	0.0438
		ETFDH-ko vs ETFDH-ko + ETFDH	<0.0001
g	Ordinary one-way ANOVA, Tukey's multiple comparisons test	CRL vs ETFDH-ko	0.0038
		CRL vs ETFDH-ko + ETFDH	0.9451
		ETFDH-ko vs ETFDH-ko + ETFDH	0.0173
h	Two-tailed Student's t-test	NDUFA9	0.0003
		UQCRC2	0.0169
**Extended data Figure 2**			
d	Two-tailed Student's t-test	CI	0.247
		CII	0.8745
		CIV	0.5216
e	Ordinary one-way ANOVA, Tukey's multiple comparisons test	CRL vs ETFDH-ko	0.0002
		CRL vs ETFDH-ko + ETFDH	0.0898
		ETFDH-ko vs ETFDH-ko + ETFDH	0.0303
f	Two-tailed Student's t-test		0.0008
g	2way ANOVA, Sidak's multiple comparisons test	VEHICLE:CRL vs. VEHICLE:ETFDH-ko	>0.9999

		VEHICLE:CRL vs. Antimycin:CRL	<0.0001
		VEHICLE:CRL vs. Antimycin:ETFDH-ko	0.0001
		VEHICLE:CRL vs. Myxothiazol:CRL	<0.0001
		VEHICLE:CRL vs. Myxothiazol:ETFDH-ko	<0.0001
		VEHICLE:ETFDH-ko vs. Antimycin:CRL	<0.0001
		VEHICLE:ETFDH-ko vs. Antimycin:ETFDH-ko	0.0001
		VEHICLE:ETFDH-ko vs. Myxothiazol:CRL	<0.0001
		VEHICLE:ETFDH-ko vs. Myxothiazol:ETFDH-ko	<0.0001
		Antimycin:CRL vs. Antimycin:ETFDH-ko	<0.0001
		Antimycin:CRL vs. Myxothiazol:CRL	0.0023
		Antimycin:CRL vs. Myxothiazol:ETFDH-ko	0.024
		Antimycin:ETFDH-ko vs. Myxothiazol:CRL	<0.0001
		Antimycin:ETFDH-ko vs. Myxothiazol:ETFDH-ko	0.0002
		Myxothiazol:CRL vs. Myxothiazol:ETFDH-ko	<0.0001
h	2way ANOVA, Sidak's multiple comparisons test	CRL: Vehicle vs. Piericidin A	<0.0001
		CRL: Vehicle vs. Rotenone	<0.0001
		CRL: Piericidin A vs. Rotenone	0.993
		ETFDH-ko: Vehicle vs. Piericidin A	<0.0001
		ETFDH-ko: Vehicle vs. Rotenone	<0.0001
		ETFDH-ko: Piericidin A vs. Rotenone	0.9105
i	2way ANOVA, Sidak's multiple comparisons test	Vehicle	0.9997
		Etomoxir	<0.0001
		- BCAA	<0.0001
		- Glycine	<0.0001
j	Ordinary one-way ANOVA, Tukey's multiple comparisons test	CRL vs ETFDH-ko	<0.0001
		CRL vs ETF-ko	<0.0001
		CRL vs Triple-ko	<0.0001
		ETFDH-ko vs ETF-ko	0.9381
		ETFDH-ko vs Triple-ko	0.9992
		ETF-ko vs Triple-ko	0.8927
k	Two-tailed Student's t-test	CoQ9H2	0.0046
		CoQ10H2	0.0186
l	Ordinary one-way ANOVA, Sidak's multiple comparisons test	Vehicle	<0.0001
		MitoQH2	0.9878

**Extended data Figure 4**			
d	Two-tailed Student's t-test		0.8386
e	Two-tailed Student's t-test		0.1536
f	Ordinary one-way ANOVA, Tukey's multiple comparisons tests	Basal: CRL vs ETFDH-ko	0.0025
		Basal: CRL vs CRL + AOX	0.2054
		Basal: CRL vs ETFDH-ko + AOX	0.0663
		Basal: ETFDH-ko vs CRL + AOX	<0.0001
		Basal: ETFDH-ko vs ETFDH-ko + AOX	0.5733
		Basal: CRL + AOX vs ETFDH-ko + AOX	0.0004
		Max: CRL vs ETFDH-ko	0.0028
		Max: CRL vs CRL + AOX	0.8022
		Max: CRL vs ETFDH-ko + AOX	0.0433
		Max: ETFDH-ko vs CRL + AOX	0.0002
		Max: ETFDH-ko vs ETFDH-ko + AOX	0.7188
		Max: CRL + AOX vs ETFDH-ko + AOX	0.0043
		Inhibited: CRL vs ETFDH-ko	0.8688
		Inhibited: CRL vs CRL + AOX	<0.0001
		Inhibited: CRL vs ETFDH-ko + AOX	<0.0001
		Inhibited: ETFDH-ko vs CRL + AOX	<0.0001
		Inhibited: ETFDH-ko vs ETFDH-ko + AOX	<0.0001
		Inhibited: CRL + AOX vs ETFDH-ko + AOX	0.0104
g	Ordinary one-way ANOVA, Tukey's multiple comparisons tests	Basal: CRL vs CRL + AOX	0.0506
		Basal: CRL vs ETFDH-ko + AOX	0.0031
		Basal: CRL + AOX vs ETFDH-ko + AOX	<0.0001
		Max: CRL vs CRL + AOX	0.0379
		Max: CRL vs ETFDH-ko + AOX	0.0007
		Max: CRL + AOX vs ETFDH-ko + AOX	<0.0001
		Inhibited: CRL vs CRL + AOX	<0.0001
		Inhibited: CRL vs ETFDH-ko + AOX	0.4671
		Inhibited: CRL + AOX vs ETFDH-ko + AOX	0.0035
**Extended data Figure 5**			
e	Ordinary one-way ANOVA, Dunnett's multiple comparisons tests	CRL vs LDL-ko	0.5792
		CRL vs PRODH-ko	0.3244
		CRL vs DHODH-ko	0.998
**Extended data Figure 6**			
a	Ordinary one-way ANOVA, Dunnett's multiple comparisons tests	CRL vs M4	<0.0001
		CRL vs M3	<0.0001
		CRL vs M2	<0.0001

		CRL vs M9	<0.0001
d	Two-tailed Student's t-test	ETFDH-UQCRC2	<0.0001
		ETFDH-COQ2	<0.0001
		UQCRC2-COQ2	0.0032
**Extended data Figure 7**			
b	Two-tailed Student's t-test	CRL	0.2614
		M9	0.6621
		M4	0.564
		M3	0.8245
		M2	0.6656
c	2way ANOVA, Dunnett's Multiple comparisons test	CRL: Vehicle vs. Riboflavin	0.7276
		CRL: Vehicle vs. Q10H2	>0.9999
		CRL: Vehicle vs. 4-CBA (3 mM)	0.5415
		CRL: Vehicle vs. 4-CBA (10 mM)	0.9155
		CRL: Vehicle vs. COQ2-ko	0.5425
		ETFDH-ko: Vehicle vs. Riboflavin	0.8458
		ETFDH-ko: Vehicle vs. Q10H2	0.0018
		ETFDH-ko: Vehicle vs. 4-CBA (3 mM)	0.0271
		ETFDH-ko: Vehicle vs. 4-CBA (10 mM)	<0.0001
		ETFDH-ko: Vehicle vs. COQ2-ko	<0.0001
e	Two-tailed Student's t-test		0.0003
f	Two-tailed Student's t-test		0.0417
g	Ordinary one-way ANOVA, Tukey's multiple comparisons test	CRL vs ETFDH-ko	0.06
		CRL vs ETFDH-ko + 4-CBA	0.1436
		ETFDH-ko vs ETFDH-ko + 4-CBA	0.9949
**Extended data Figure 8**			
b	Two-tailed Student's t-test	females	0.0214
		males	0.6991
c	2way ANOVA, Šídák's multiple comparisons test	Females: Fed	>0.9999
		Females: Fasting	<0.0001
		Males: Fed	0.8793
		Males: Fasting	0.0002
d	2way ANOVA, Šídák's multiple comparisons test	Females: 8	>0.9999
		Females: 16	0.9968
		Females: 24	0.9293
		Females: 32	0.0006
		Females: 40	<0.0001
		Males: 8	>0.9999
		Males: 16	0.6169
		Males: 24	0.2968
		Males: 32	0.0047
		Males: 40	0.0001
e	2way ANOVA, Šídák's multiple comparisons test	All: Fed	0.2085
		All: Fasting	<0.0001

		Females: Fed	0.8239
		Females: Fasting	<0.0001
		Males: Fed	0.266
		Males: Fasting	0.0194
f	2way ANOVA, Šídák's multiple comparisons test	All: 8	>0.9999
		All: 16	0.7675
		All: 24	0.0006
		All: 32	<0.0001
		All: 40	<0.0001
		Females: 8	>0.9999
		Females: 16	0.9994
		Females: 24	0.0273
		Females: 32	<0.0001
		Females: 40	<0.0001
		Males: 8	>0.9999
		Males: 16	0.7381
		Males: 24	0.0557
		Males: 32	0.002
		Males: 40	<0.0001
g	Two-tailed Student's t-test		0.07

Table 3. Exact p values for each graph in Figures and Extended Data Figures.

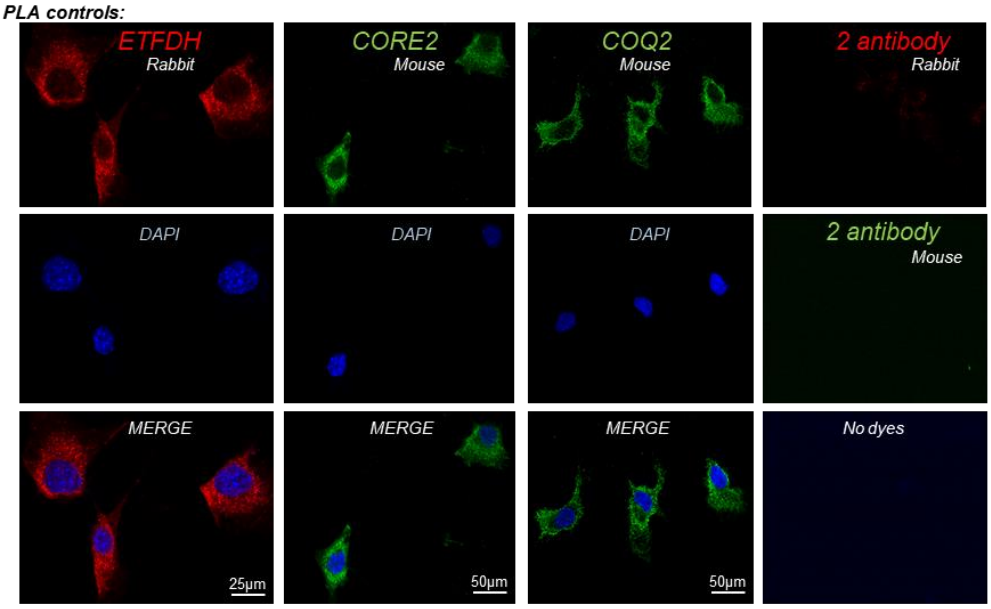

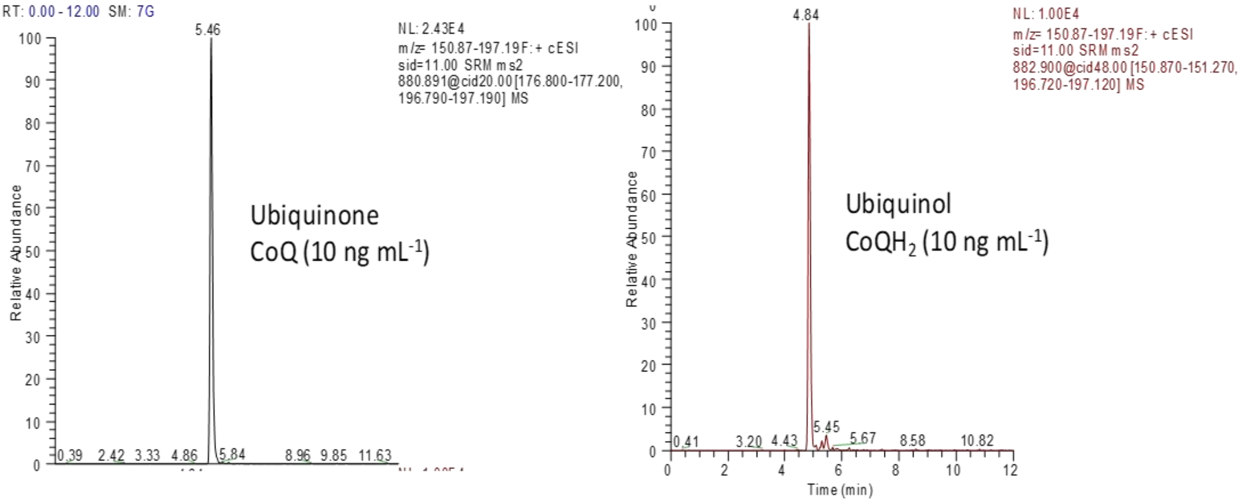

Supplementary Fig. 1

a) Controls for Proximity ligation assay (PLA) in Fig 1Q and 3J. Specific mitochondrial staining for ETFDH, CIII-CORE2 and COQ2 are shown. DAPI was used for nuclear staining. Anti-mouse and anti-rabbit secondary antibody staining without preincubation with primary antibody was also shown.

b) Representative HPLC-MS/MS spectrum of oxidized (Q) and reduced (QH_2) coenzyme Q standards.

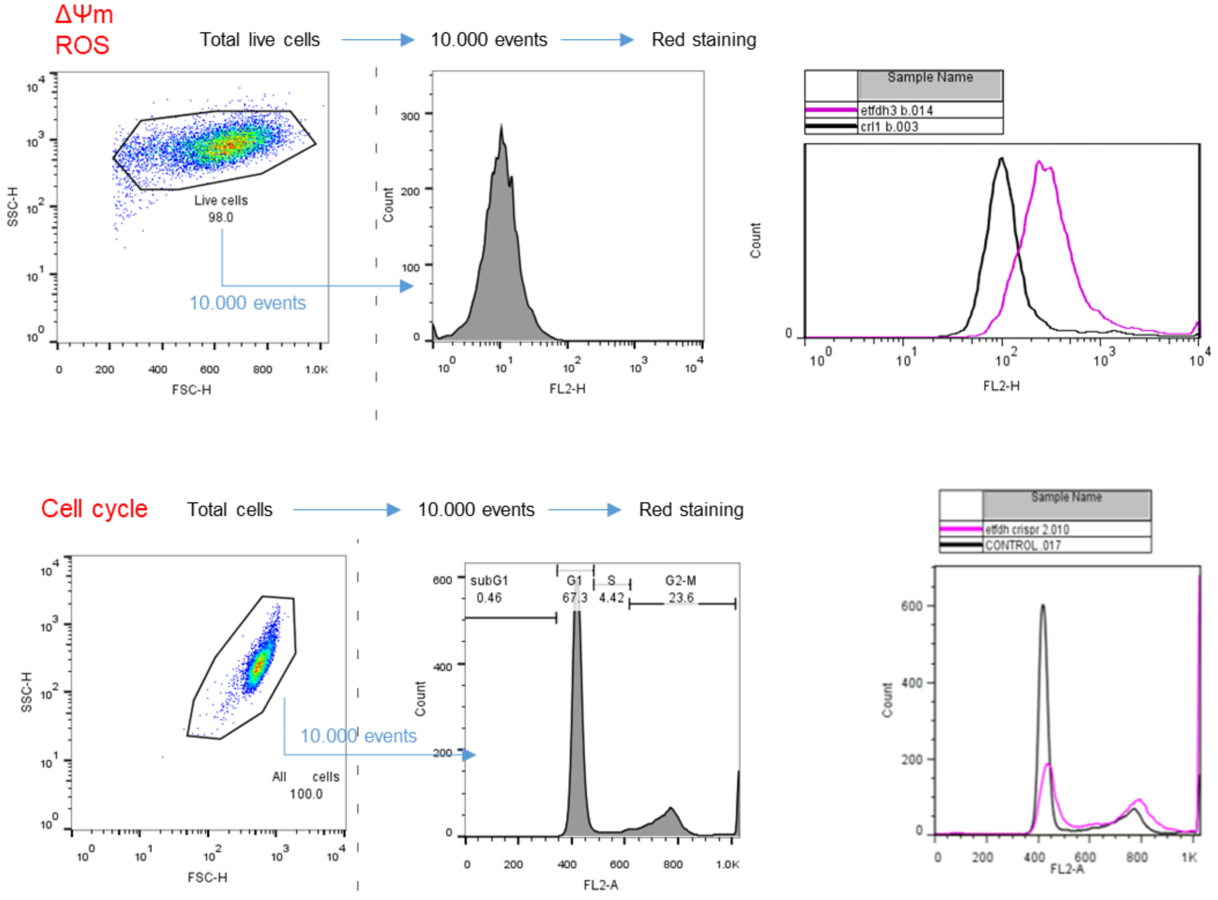

Supplementary Fig. 2

Gating strategies for flow-cytometry experiments.
