# Supplementary material for: An ETFDH-driven metabolon supports OXPHOS efficiency in skeletal muscle by regulating coenzyme Q homeostasis
Source: Nat Metab. 2024 Jan 19;6(2):209–25. doi: 10.1038/s42255-023-00956-y (PMC10896730; doi:10.1038/s42255-023-00956-y)
Supplement: Supplementary file 2 — Reporting Summary [file 42255_2023_956_MOESM2_ESM.pdf]

nature portfolio

Corresponding author(s): Laura Formentini

Last updated by author(s): Nov 2, 2023

Reporting Summary

Nature Portfolio wishes to improve the reproducibility of the work that we publish. This form provides structure for consistency and transparency in reporting. For further information on Nature Portfolio policies, see our Editorial Policies and the Editorial Policy Checklist.

Statistics

For all statistical analyses, confirm that the following items are present in the figure legend, table legend, main text, or Methods section.

n/a	Confirmed	
☐	☒	The exact sample size (n) for each experimental group/condition, given as a discrete number and unit of measurement
☐	☒	A statement on whether measurements were taken from distinct samples or whether the same sample was measured repeatedly
☐	☒	The statistical test(s) used AND whether they are one- or two-sided<br>*Only common tests should be described solely by name; describe more complex techniques in the Methods section.*
☒	☐	A description of all covariates tested
☐	☒	A description of any assumptions or corrections, such as tests of normality and adjustment for multiple comparisons
☐	☒	A full description of the statistical parameters including central tendency (e.g. means) or other basic estimates (e.g. regression coefficient) AND variation (e.g. standard deviation) or associated estimates of uncertainty (e.g. confidence intervals)
☐	☒	For null hypothesis testing, the test statistic (e.g. F, t, r) with confidence intervals, effect sizes, degrees of freedom and P value noted<br>*Give P values as exact values whenever suitable.*
☒	☐	For Bayesian analysis, information on the choice of priors and Markov chain Monte Carlo settings
☒	☐	For hierarchical and complex designs, identification of the appropriate level for tests and full reporting of outcomes
☒	☐	Estimates of effect sizes (e.g. Cohen's d, Pearson's r), indicating how they were calculated

Our web collection on statistics for biologists contains articles on many of the points above.

Software and code

Policy information about availability of computer code

Data collection	Numerical simulations were performed using Julia language and the package DifferentialEquations.jl.
Data analysis	Comprehensive information on all software used in this study is provided in Table S2.

For manuscripts utilizing custom algorithms or software that are central to the research but not yet described in published literature, software must be made available to editors and reviewers. We strongly encourage code deposition in a community repository (e.g. GitHub). See the Nature Portfolio guidelines for submitting code & software for further information.

Data

Policy information about availability of data

All manuscripts must include a data availability statement. This statement should provide the following information, where applicable:

- Accession codes, unique identifiers, or web links for publicly available datasets
- A description of any restrictions on data availability
- For clinical datasets or third party data, please ensure that the statement adheres to our policy

Data supporting this study are included within the article, supporting materials, or have been deposited in public repositories. Accession for proteomis: ProteomeXchange via the PRIDE database: Project Name: C2C12 myoblast wt vs ETFDH-ko TMT. Project accession: PXD041825. Project DOI: 10.6019/PXD041825. Project Name: ETFDH, CIII and COQ2 common interactome. Project accession: PXD045351. Project DOI: 10.6019/PXD045351. Project Name: ETFDH comigrates with CIII in BN-PAGE. Project accession: PXD045352. Project DOI: 10.6019/PXD045352. Project name: Antimycin A-treated vs ETFDH-ko myoblasts TMT. Project

accession: PXD045588. Project DOI: 10.6019/PXD045588. Professor J.A. Enriquez kindly shared proteomics data available in Calvo et al.21 . PDB ID for ETFDH: 2GMH.
All materials are available from the corresponding author upon reasonable request or material transfer agreement.

Research involving human participants, their data, or biological material

Policy information about studies with human participants or human data. See also policy information about sex, gender (identity/presentation), and sexual orientation and race, ethnicity and racism.

Reporting on sex and gender	Patient-derived cells and patients' urine and blood were collected for genetic diagnosis and studies from individuals suspected of having an inborn error of metabolism at CEDEM, Spain (ethical approvals: CEI-129-2655, CEI-105-2052) and stored at the CEDEM Biobank. In the method section, we reported the sex of the patients from whom the cells are derived. However, no prevalence of MADD-disease in males or females have been reported. Our findings apply to both sexes.
Reporting on race, ethnicity, or other socially relevant groupings	no information on population characteristics was collected for the study
Population characteristics	Pediatric age; no additional information on population characteristics is collected for the study.
Recruitment	individuals suspected of having an inborn error of metabolism
Ethics oversight	All human studies were performed following EU ethical guidelines and have the approval of Institutional Committees (UAM University and Madrid Community, Spain; CEI-129-2655, CEI-105-2052). We have obtained informed consent from all participants. Participants have not received compensation for this study.

Note that full information on the approval of the study protocol must also be provided in the manuscript.

Field-specific reporting

Please select the one below that is the best fit for your research. If you are not sure, read the appropriate sections before making your selection.

☒ Life sciences ☐ Behavioural & social sciences ☐ Ecological, evolutionary & environmental sciences

For a reference copy of the document with all sections, see nature.com/documents/nr-reporting-summary-flat.pdf

Life sciences study design

All studies must disclose on these points even when the disclosure is negative.

Sample size	In order to minimize the number of animals we used power analysis to calculate the minimum sample size using the free software DOEUMH (https://samplesizeumh.shinyapps.io/DOEUMH) based on the TrialSize library of the R program (R Core Team). We selected the procedure KMeans – ANOVA, fixing the significance to 0.05, power to 0.08 and a drop-out of 5%. We took into consideration differences between averages of about 1.5-2 fold. Minimum number of mice/group: 8 mice/group.
Data exclusions	No exclusions
Replication	Data represented in this study are the mean ± SEM of at least 3 experiments. All attempts at replications were successful.
Randomization	Randomization was assessed by equally distributing experimental groups across multiple cages, and balancing the location of the mouse cages on the racks.
Blinding	All tests were performed in a blinded fashion.

Reporting for specific materials, systems and methods

We require information from authors about some types of materials, experimental systems and methods used in many studies. Here, indicate whether each material, system or method listed is relevant to your study. If you are not sure if a list item applies to your research, read the appropriate section before selecting a response.

Materials & experimental systems

n/a	Involved in the study	
☐	☒	Antibodies
☐	☒	Eukaryotic cell lines
☒	☐	Palaeontology and archaeology
☐	☒	Animals and other organisms
☒	☐	Clinical data
☒	☐	Dual use research of concern
☒	☐	Plants

Methods

n/a	Involved in the study	
☒	☐	ChIP-seq
☐	☒	Flow cytometry
☒	☐	MRI-based neuroimaging

Antibodies

Antibodies used	Table S2 contains information about all antibodies, including the supplier name, RRID, reference, molecular weight, origin, and details about the protocol.
Validation	All antibodies were validated by manufacturer. We validated them on mouse tissue and cell lines (previous results were reported in Sanchez-Gonzalez C. et al., EMBO J 2020, 39, e103812).

Eukaryotic cell lines

Policy information about cell lines and Sex and Gender in Research

Cell line source(s)	We used: Mouse-derived primary myoblasts (from male mice); C2C12 mouse myoblasts (ATCC), CRL and AOX-expressing mouse fibroblasts (kindly gifted by Professor José Antonio Enriquez and used in PMID: 27052170) and patient-derived fibroblasts (collected for genetic diagnosis from individuals suspected of having an inborn error of metabolism at CEDEM, Spain, ethical approvals: CEI-129-2655, CEI-105-2052).
Authentication	C2C12: ATCC, catalog number: CRL-1772, recently purchased and validated by morphology
Mycoplasma contamination	We confirm that all cell lines tested negative for mycoplasma.
Commonly misidentified lines (See ICLAC register)	*Name any commonly misidentified cell lines used in the study and provide a rationale for their use.*

Animals and other research organisms

Policy information about studies involving animals; ARRIVE guidelines recommended for reporting animal research, and Sex and Gender in Research

Laboratory animals	Animal studies were performed following EU ethical and ARRIVE guidelines.<br>The B6;C3-Tg(Acta1-rtTA,tetO-cre, Skm-Cre mice)102MonK/J mouse was purchased from The Jackson Laboratories. The Etfdh-tm1a (EUCOMM) Hmgu (EMMA ID:09069) mouse was purchased from EMMA INFRAFRONTIER and bred with the C57BL/6-FLPe mice to obtain the Etfdh-Tm1c mouse (E mice). The Etfdh-/+, Acta1-Cre+ (Etfdh+/-) and Etfdh-/-, Acta1-Cre+ (Etfdh-/-) mice were obtained by breeding Skm-Cre with E mice during 2 generations. Mice were maintained on the (C57BL/6x C3H)F2 background. Animals were maintained in 12 h light/12 h dark, ~18-23°C with 40-60% humidity. Administration of 2 mg/mL doxycycline in the drinking water for at least 1 week was used to turn on the Skm expression of the Cre protein. All experiments were performed on age-matched 4- and 6-month-old male and female littermate wt and Etfdh-/- mice.
Wild animals	no wild animals were used in the study.
Reporting on sex	Male and female mice were used.
Field-collected samples	no field-collected samples were used in the study.
Ethics oversight	Procedures have the approval of the Institutional Review Board (UAM University and Madrid Community Ethical Committees, Spain; PROEX 183/17, PROEX 207.5/22).

Note that full information on the approval of the study protocol must also be provided in the manuscript.

Flow Cytometry

Plots

Confirm that:

☒ The axis labels state the marker and fluorochrome used (e.g. CD4-FITC).

☒ The axis scales are clearly visible. Include numbers along axes only for bottom left plot of group (a 'group' is an analysis of identical markers).

☒ All plots are contour plots with outliers or pseudocolor plots.

☒ A numerical value for number of cells or percentage (with statistics) is provided.

Methodology

Sample preparation	PI, MitoSox, H2DCFDA or TMRM dyes were used. Cells were stained and resuspended in FACS.
Instrument	BD FACScan
Software	FlowJo software v10.6.2.
Cell population abundance	10.000 events (cells) were analyzed
Gating strategy	All cells were gated and the intensity of red staining (dye) measured.

☒ Tick this box to confirm that a figure exemplifying the gating strategy is provided in the Supplementary Information.
