# Supplementary material for: An ETFDH-driven metabolon supports OXPHOS efficiency in skeletal muscle by regulating coenzyme Q homeostasis
Source: Nat Metab. 2024 Jan 19;6(2):209–25. doi: 10.1038/s42255-023-00956-y (PMC10896730; doi:10.1038/s42255-023-00956-y)

Figure 1

b

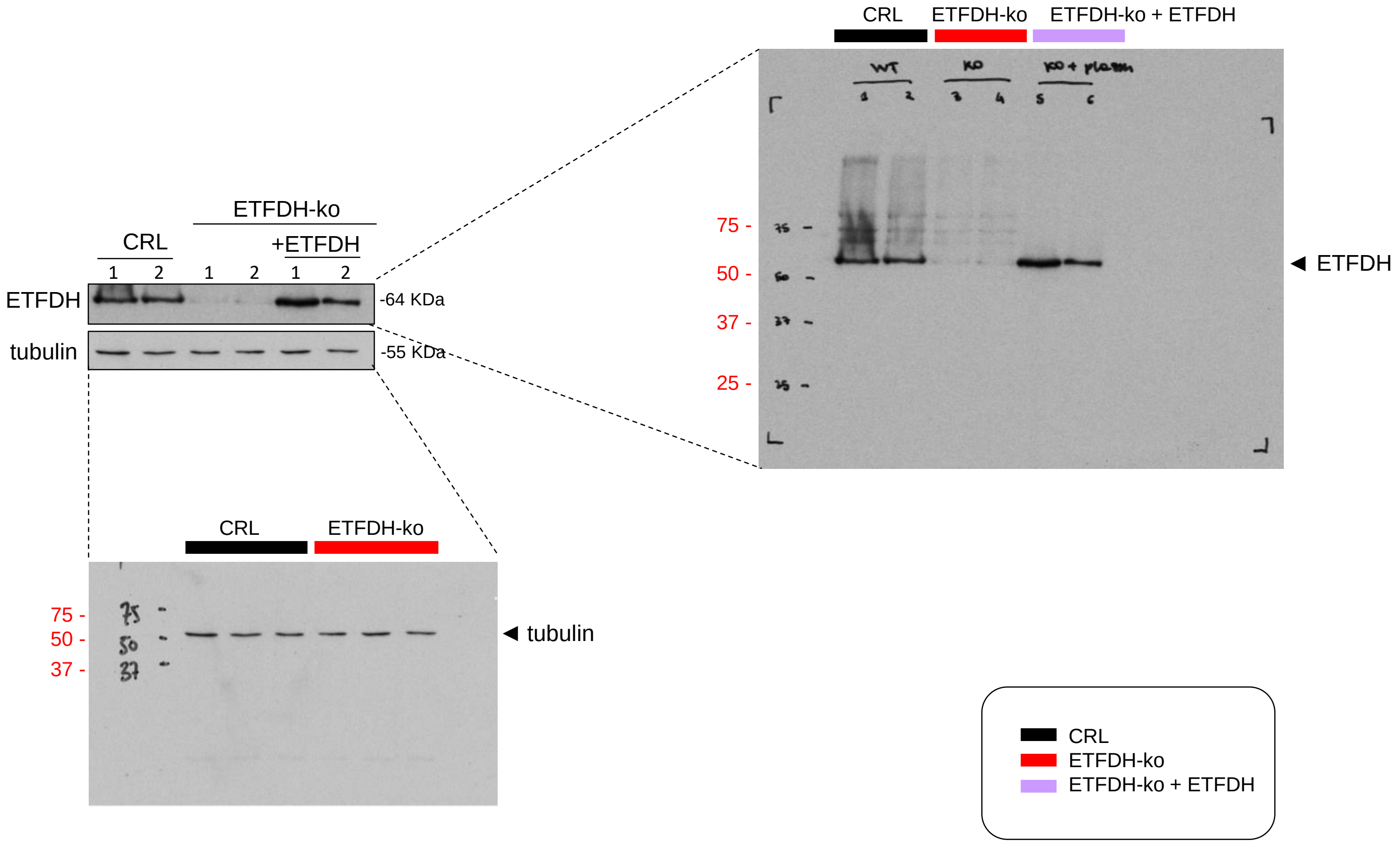

ETFDH-ko
CRL
+ETFDH
1
2
1
2
1
2
ETFDH
tubulin
-64 KDa
-55 KDa
CRL
ETFDH-ko
ETFDH-ko + ETFDH
75 -
50 -
37 -
25 -
◄ ETFDH
CRL
ETFDH-ko
75 -
50 -
37 -
◄ tubulin
CRL
ETFDH-ko
ETFDH-ko + ETFDH

Figure 1

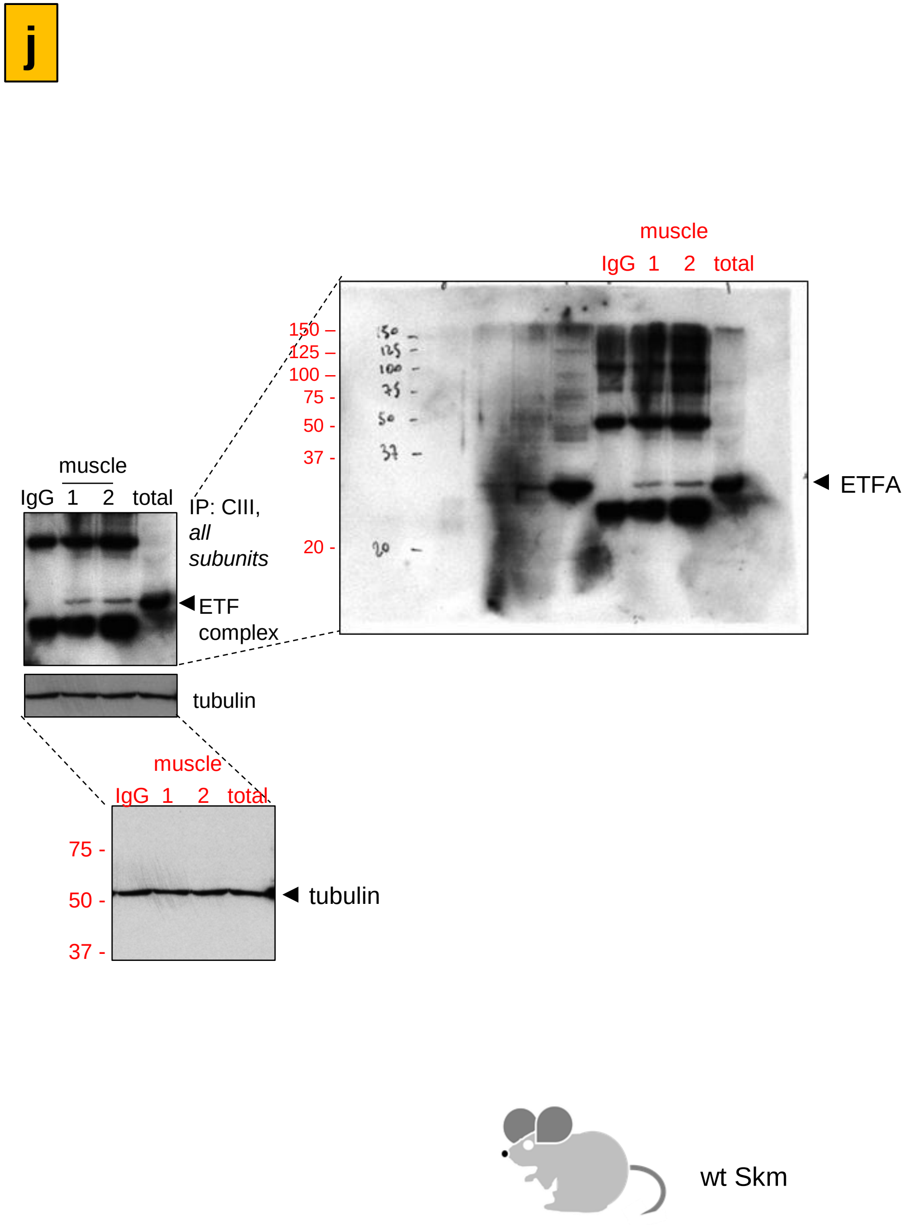
j
muscle
IgG 1 2 total
IP: CIII,
all
subunits
ETF
complex
tubulin
150 –
125 –
100 –
75 -
50 -
37 -
20 -
ETFA
muscle
IgG 1 2 total
75 -
50 -
37 -
tubulin
wt Skm

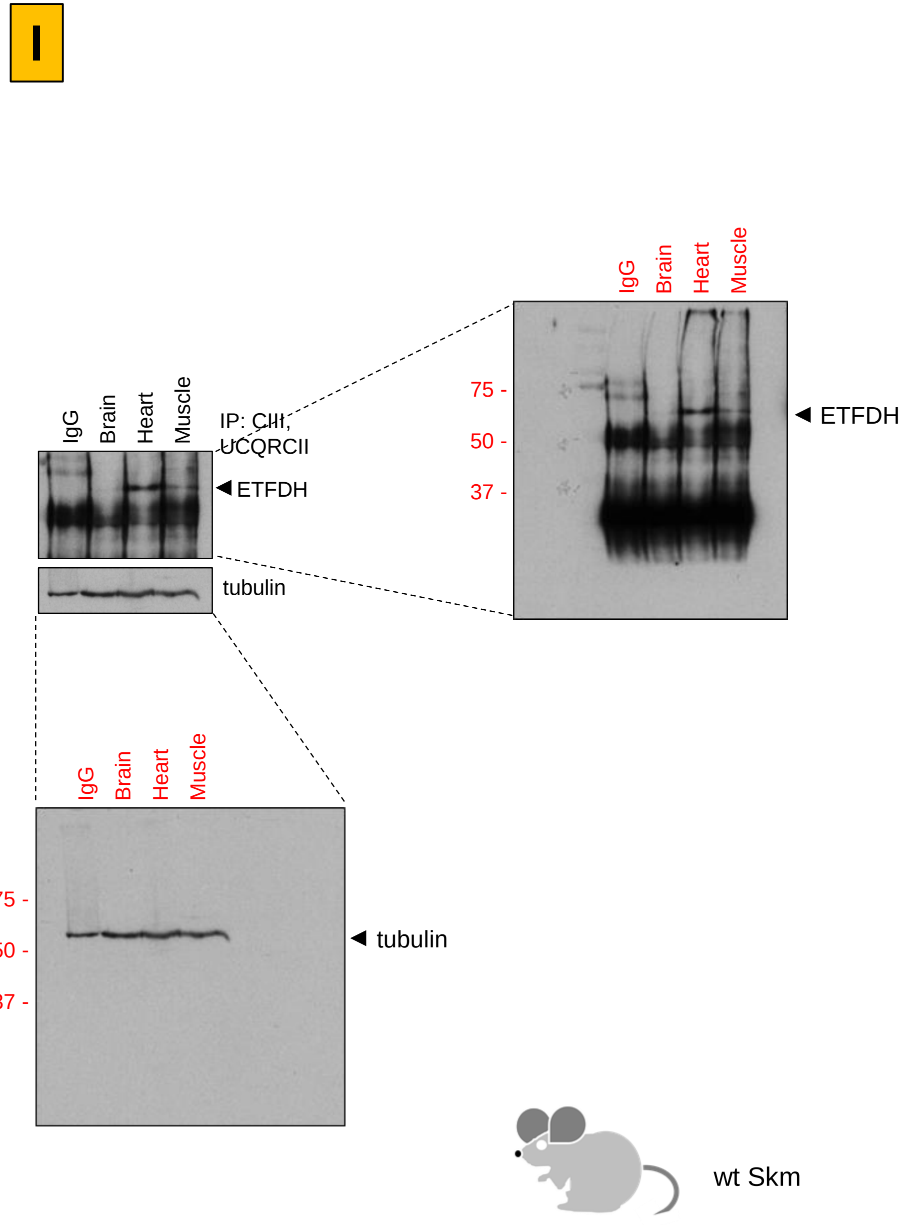
l
IgG
Brain
Heart
Muscle
IP: CIII,
UCQRCII
ETFDH
tubulin
75 -
50 -
37 -
ETFDH
IgG
Brain
Heart
Muscle
75 -
50 -
37 -
tubulin
wt Skm

Figure 1

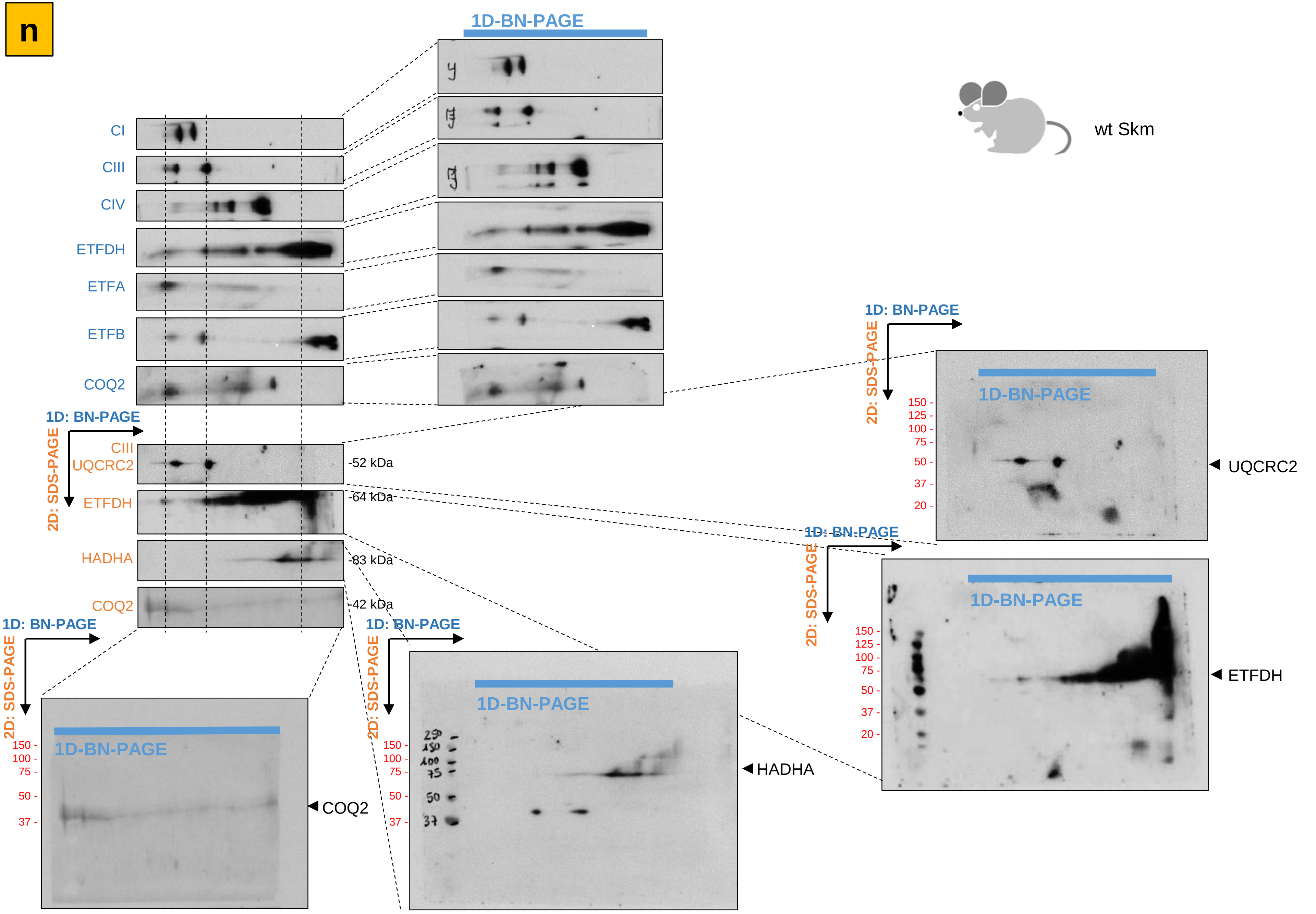
n
1D-BN-PAGE
wt Skm
CI
CIII
CIV
ETFDH
ETFA
ETFB
COQ2
1D: BN-PAGE
2D: SDS-PAGE
CIII
UQCRC2
ETFDH
HADHA
COQ2
-52 kDa
-64 kDa
-83 kDa
-42 kDa
UQCRC2
ETFDH
HADHA
COQ2
150 -
125 -
100 -
75 -
50 -
37 -
20 -

Figure 2

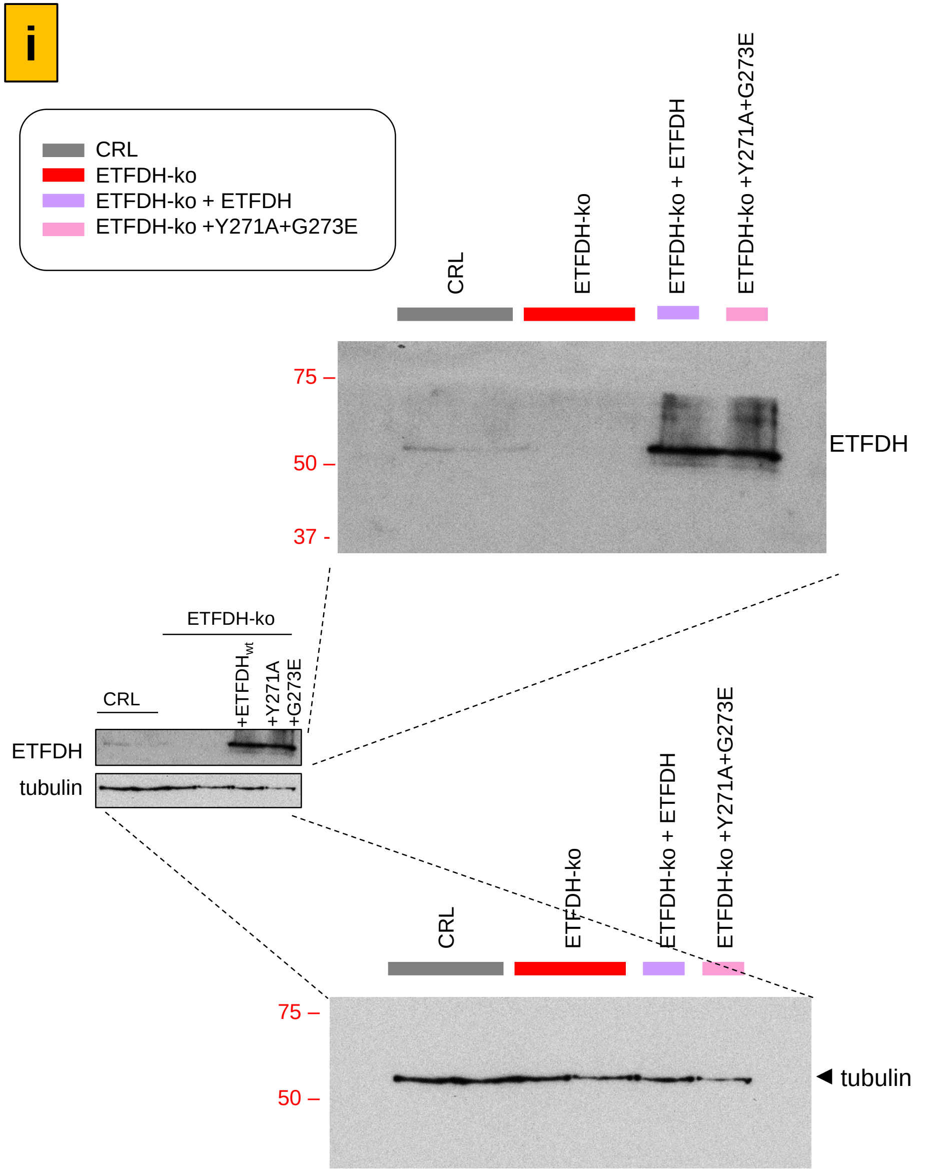

i
CRL
ETFDH-ko
ETFDH-ko + ETFDH
ETFDH-ko +Y271A+G273E
75 –
50 –
37 -
ETFDH
ETFDH-ko
CRL
+ETFDH$_{wt}$
+Y271A
+G273E
ETFDH
tubulin
75 –
50 –
tubulin

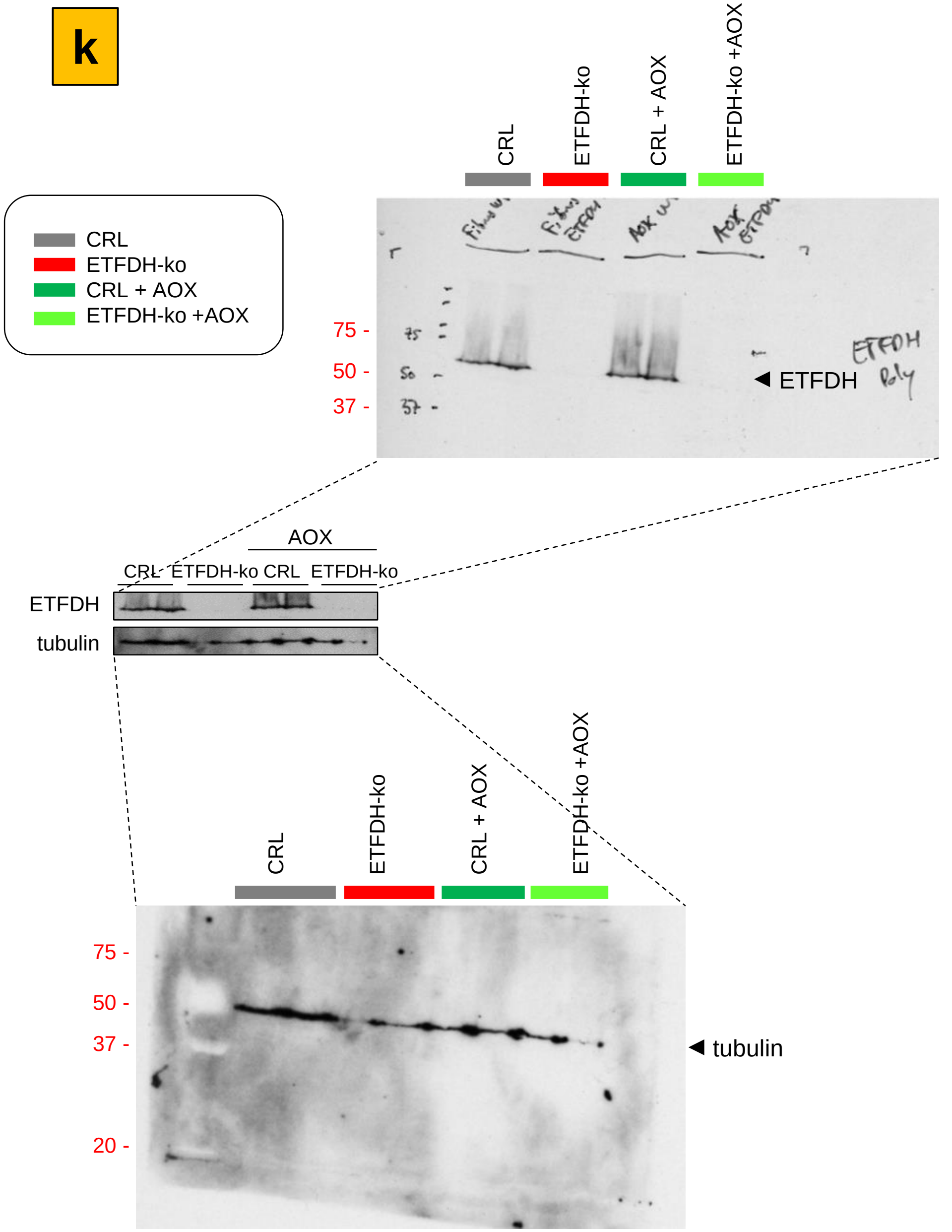

k
CRL
ETFDH-ko
CRL + AOX
ETFDH-ko +AOX
75 -
50 -
37 -
ETFDH
AOX
CRL
ETFDH-ko
CRL
ETFDH-ko
ETFDH
tubulin
75 -
50 -
37 -
20 -
tubulin

Figure 3

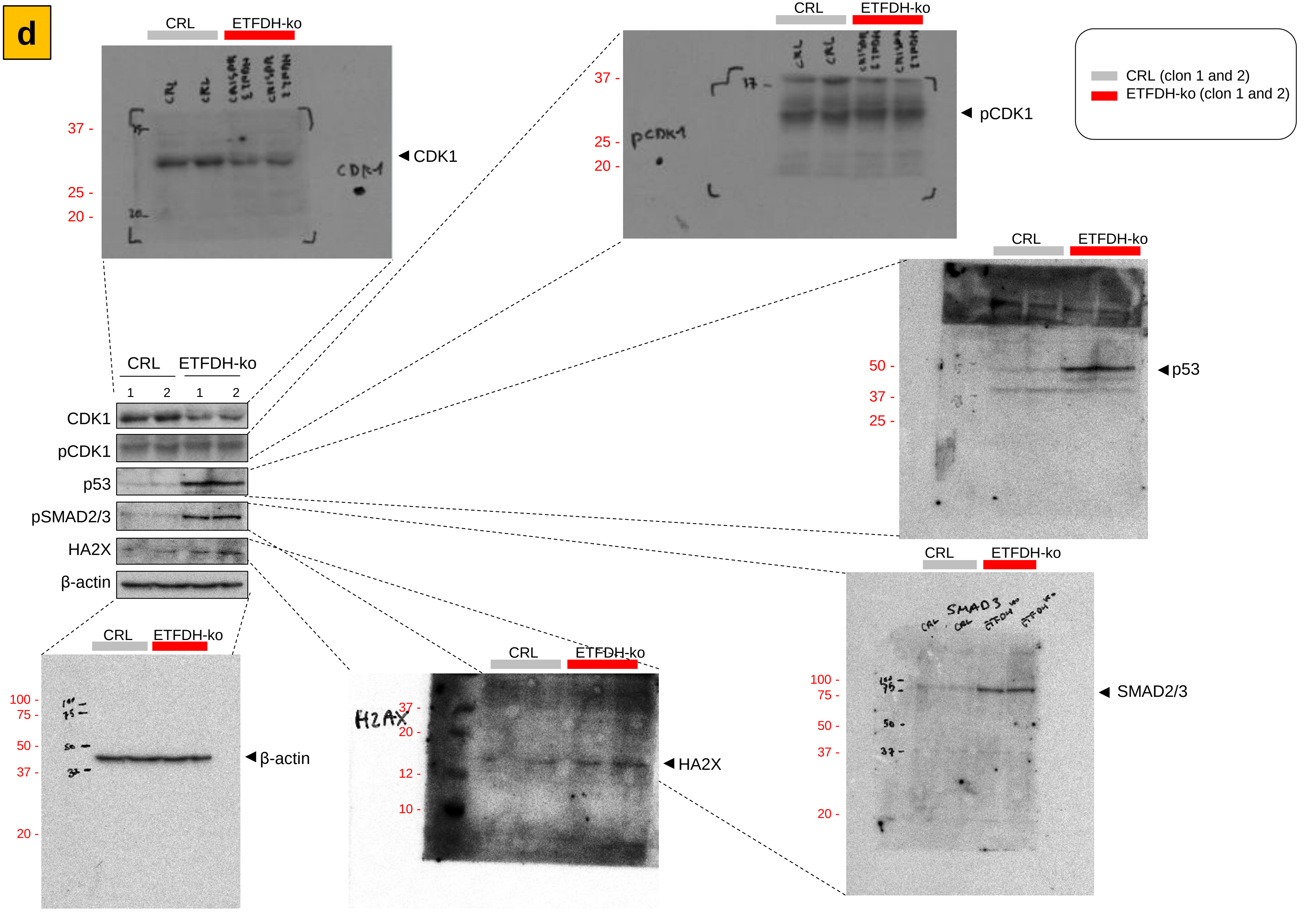

d
CRL
ETFDH-ko
CRL (clon 1 and 2)
ETFDH-ko (clon 1 and 2)
CDK1
pCDK1
p53
pSMAD2/3
HA2X
β-actin
SMAD2/3
37 -
25 -
20 -
50 -
100 -
75 -
12 -
10 -
1
2

Figure 3

i

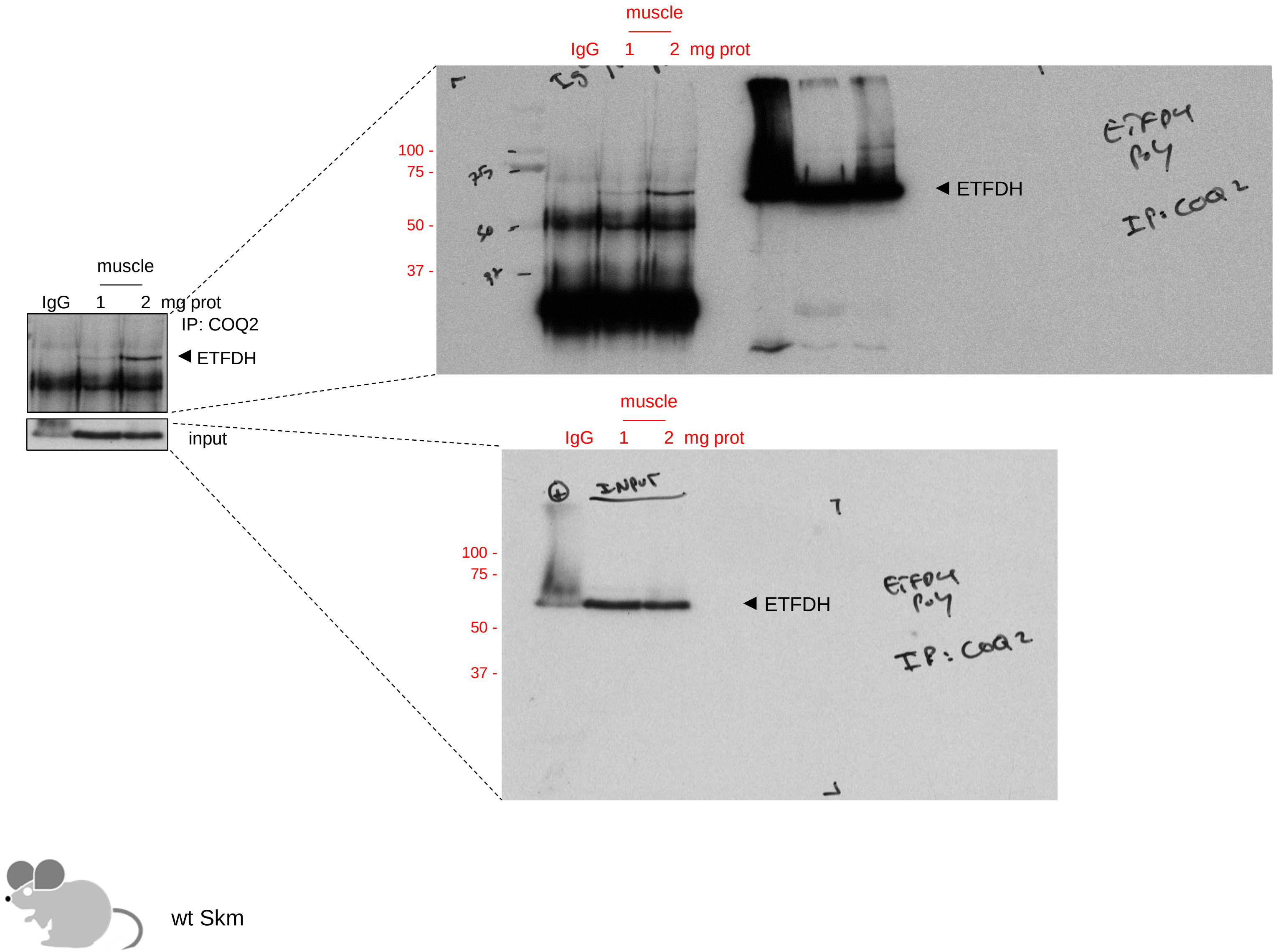
muscle
IgG 1 2 mg prot
IP: COQ2
ETFDH
input
muscle
IgG 1 2 mg prot
100 -
75 -
50 -
37 -
ETFDH
muscle
IgG 1 2 mg prot
100 -
75 -
50 -
37 -
ETFDH
wt Skm

Figure 4

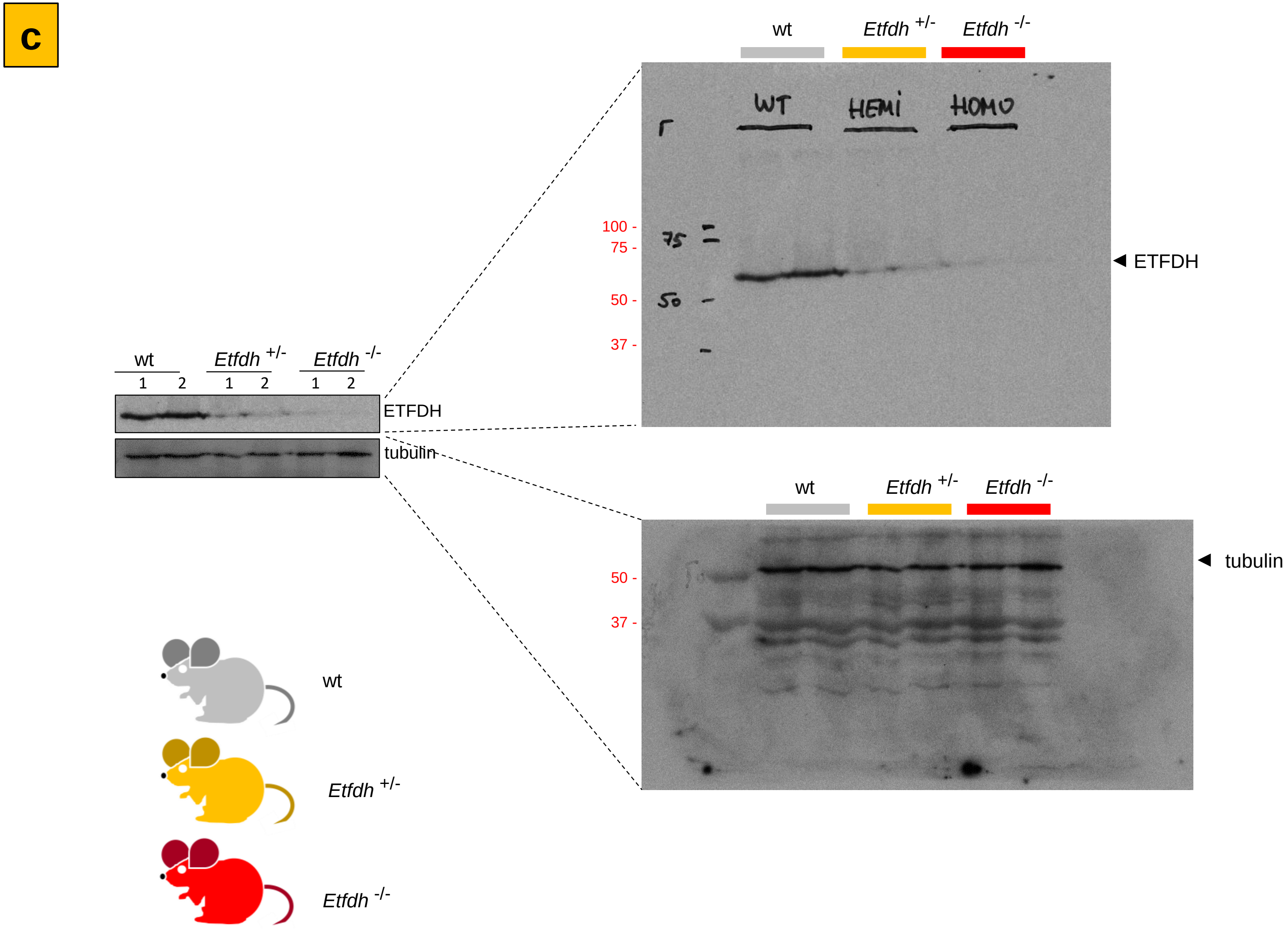
c
wt
Etfdh +/-
Etfdh -/-
1 2 1 2 1 2
ETFDH
tubulin
WT
HEMI
HOMO
100 -
75 -
50 -
37 -
75
50
ETFDH
wt
Etfdh +/-
Etfdh -/-
50 -
37 -
tubulin
wt
Etfdh +/-
Etfdh -/-

Figure 4

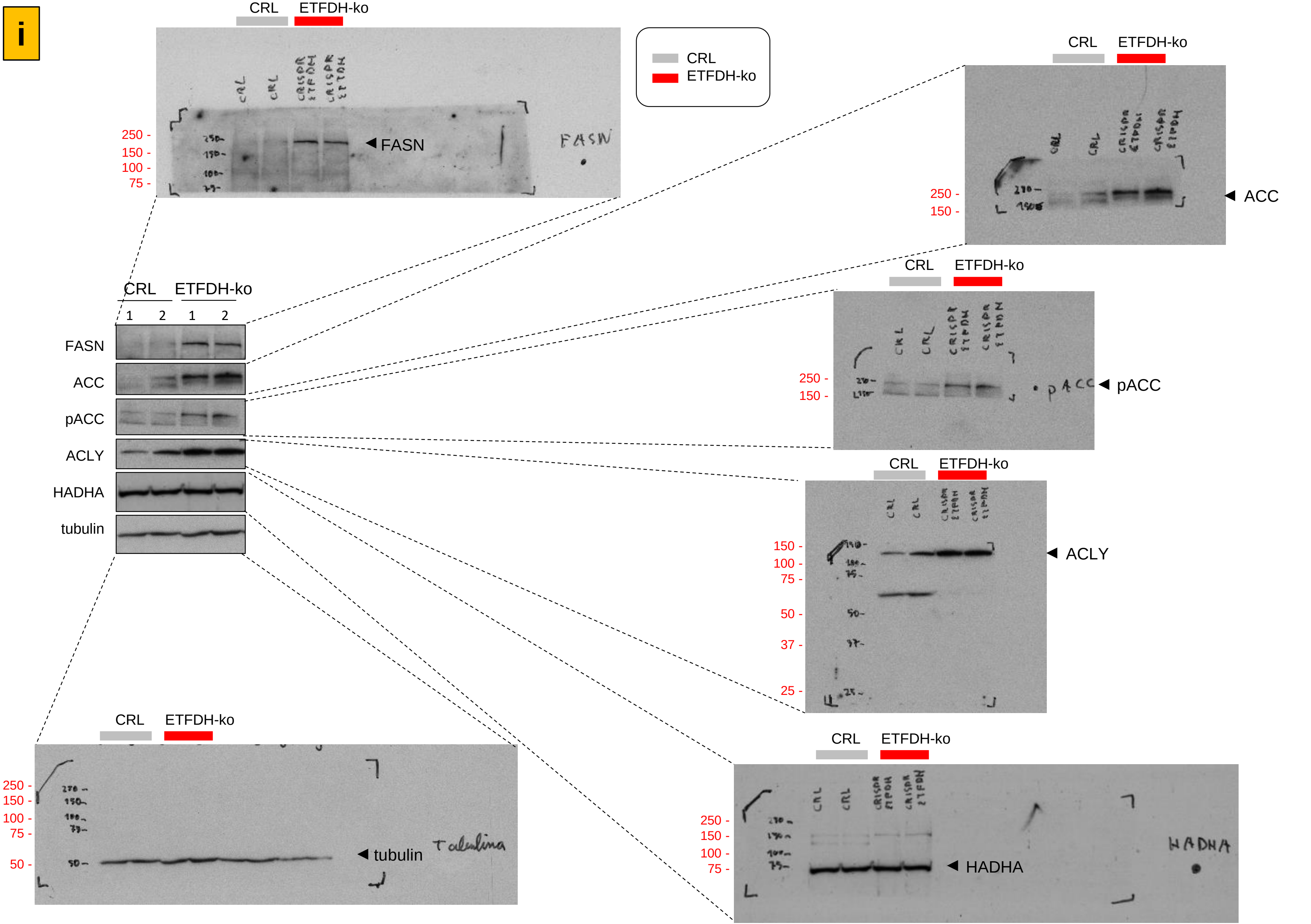

i
CRL
ETFDH-ko
FASN
ACC
pACC
ACLY
HADHA
tubulin
1
2
250 -
150 -
100 -
75 -
50 -
37 -
25 -

Extended data Figure 1

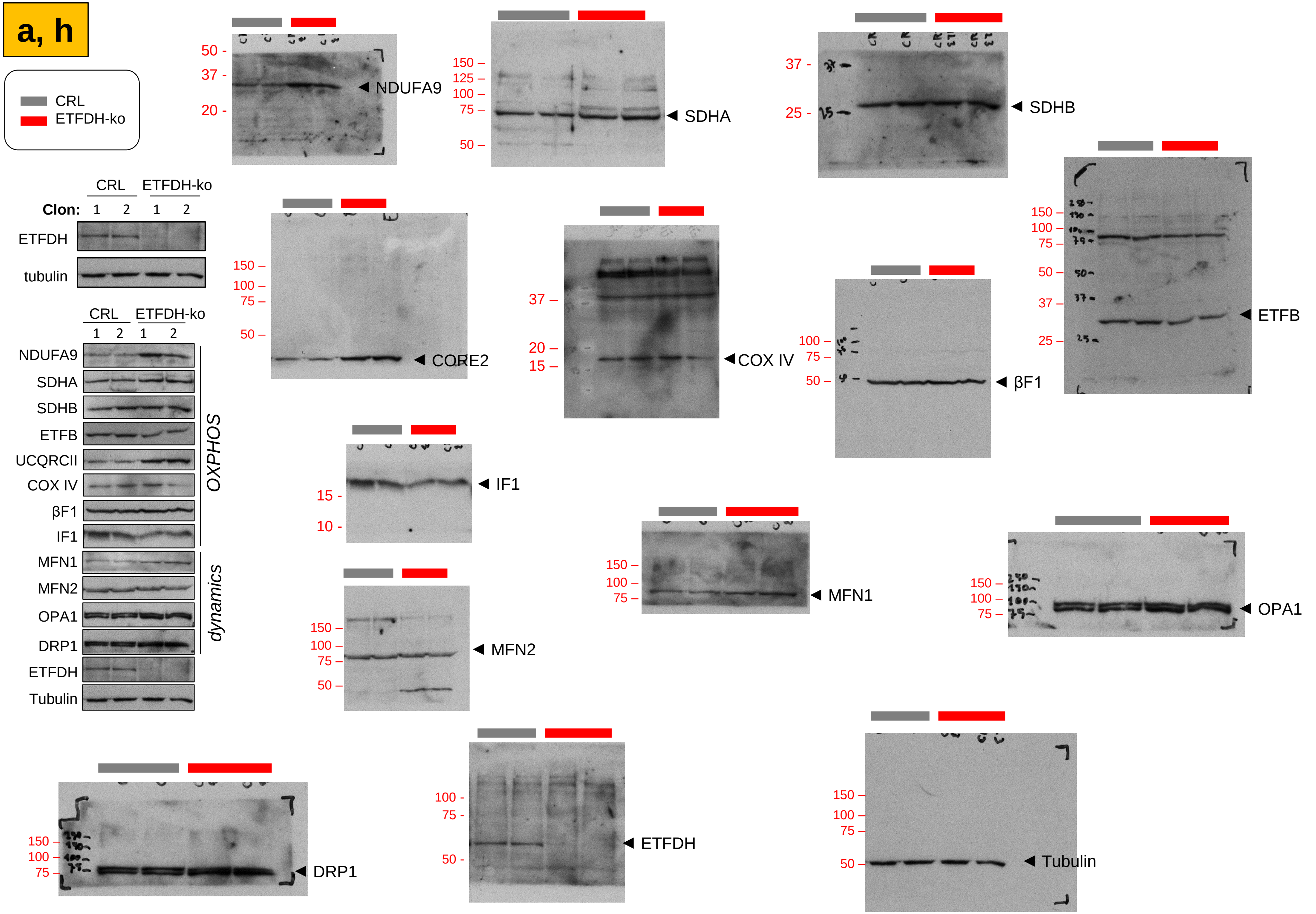

Extended data Figure 1

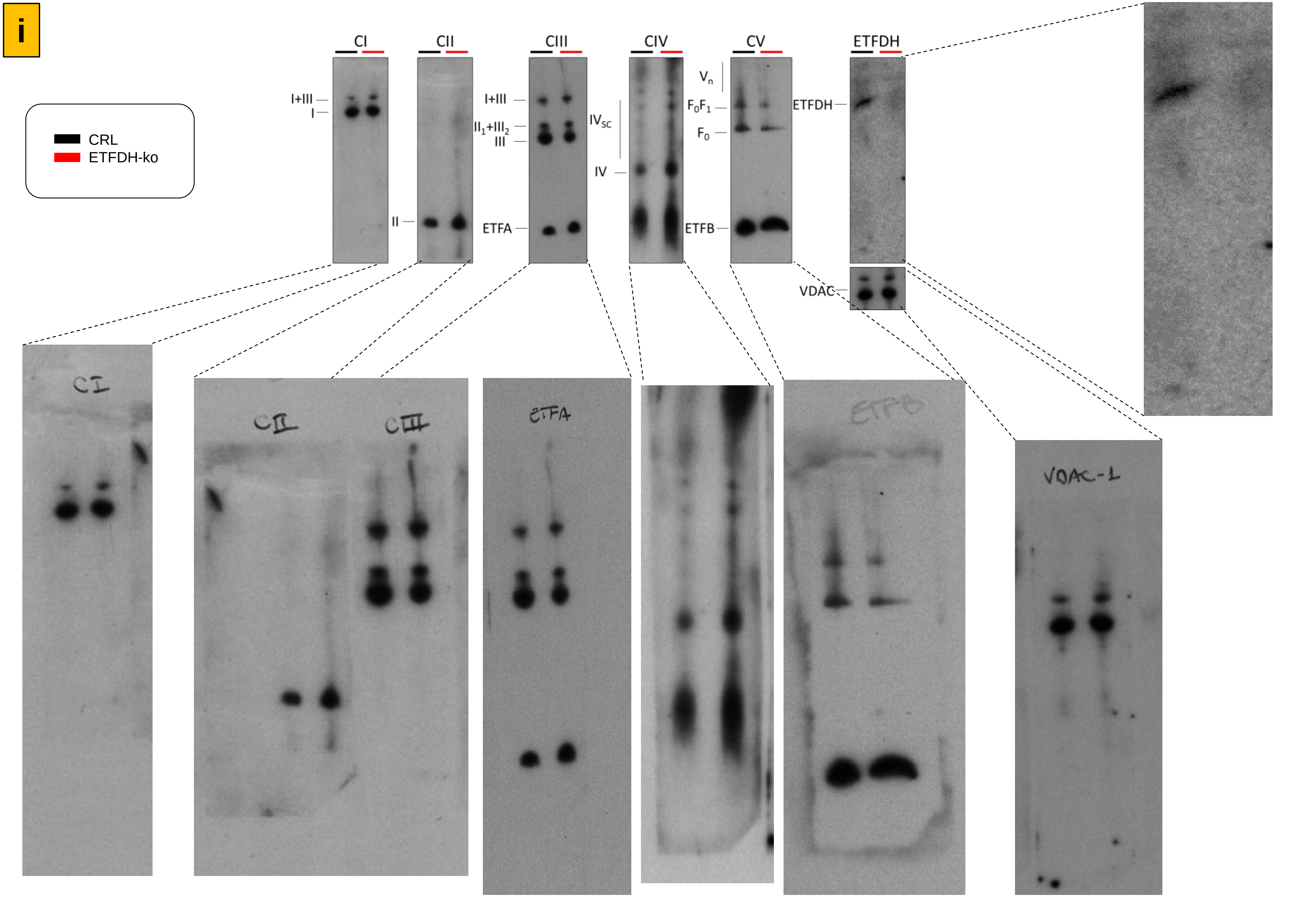

Extended data Figure 1

l

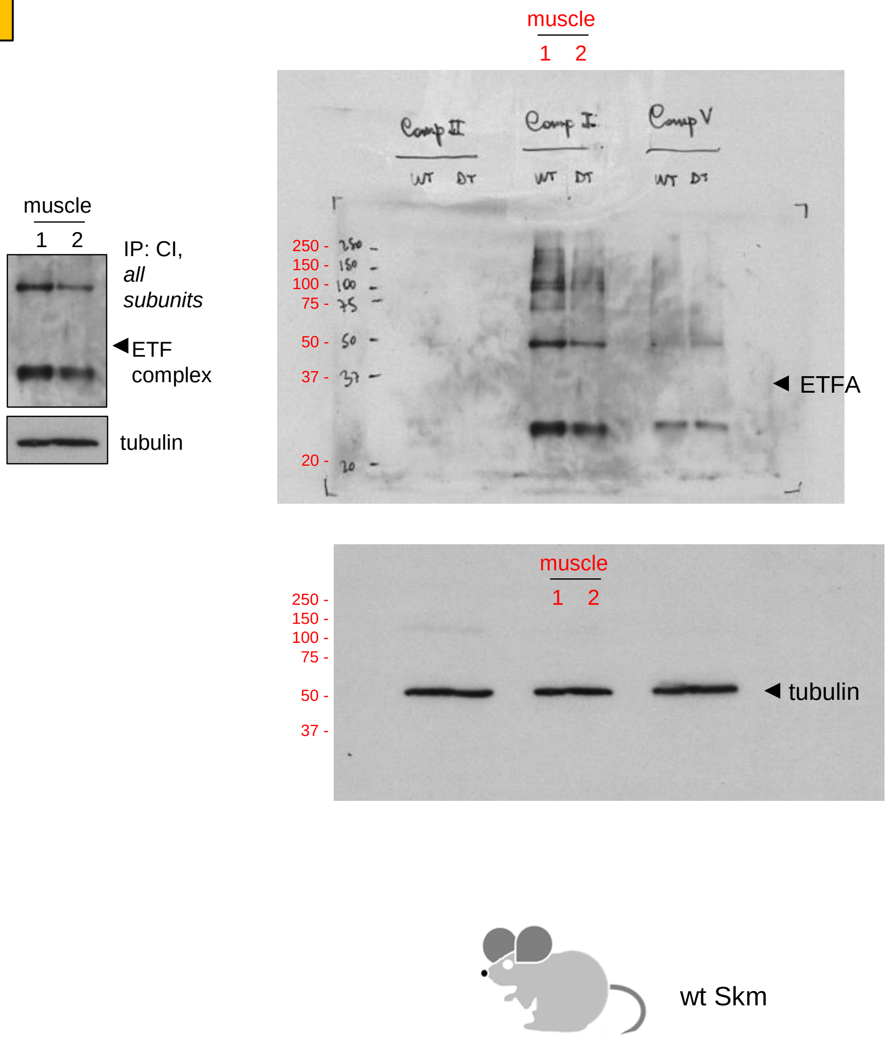

i

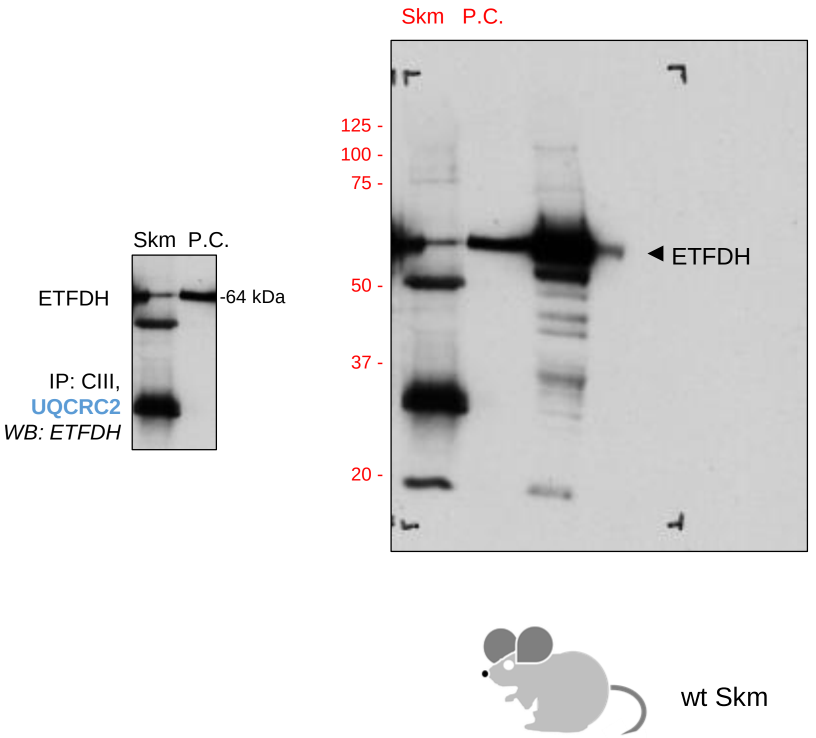

Extended data Figure 2

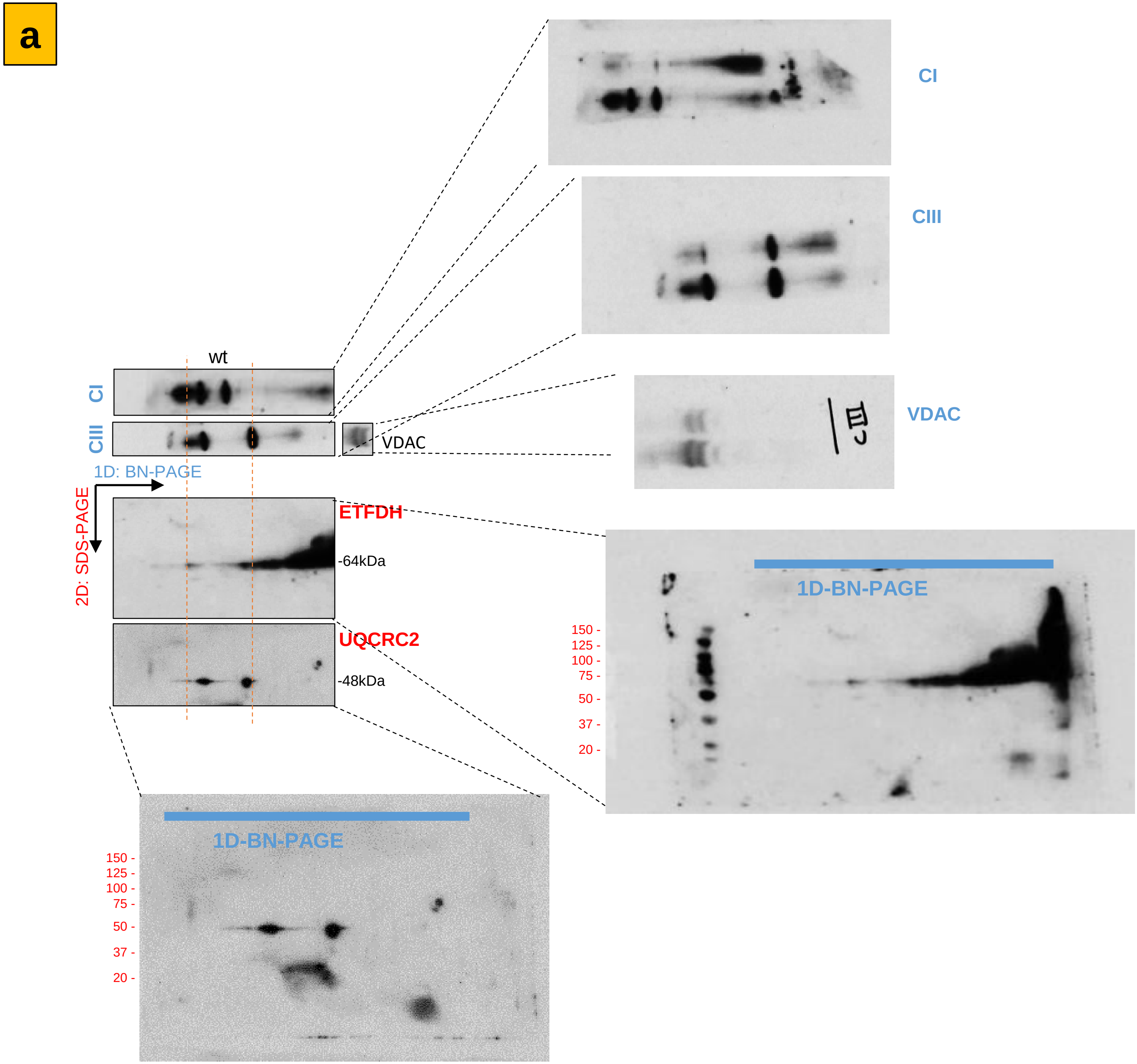

Extended data Figure 2

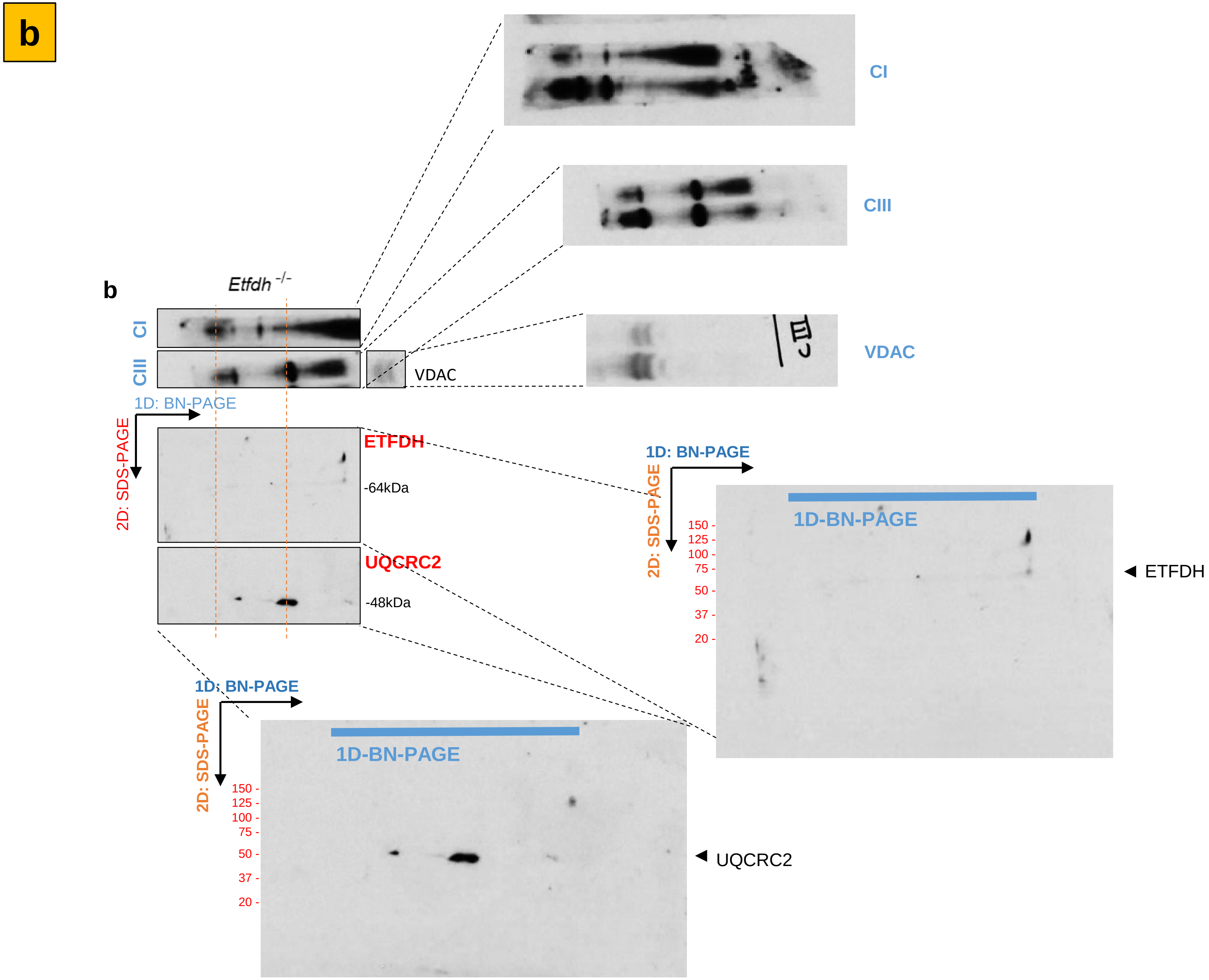

Extended data Figure 2

c

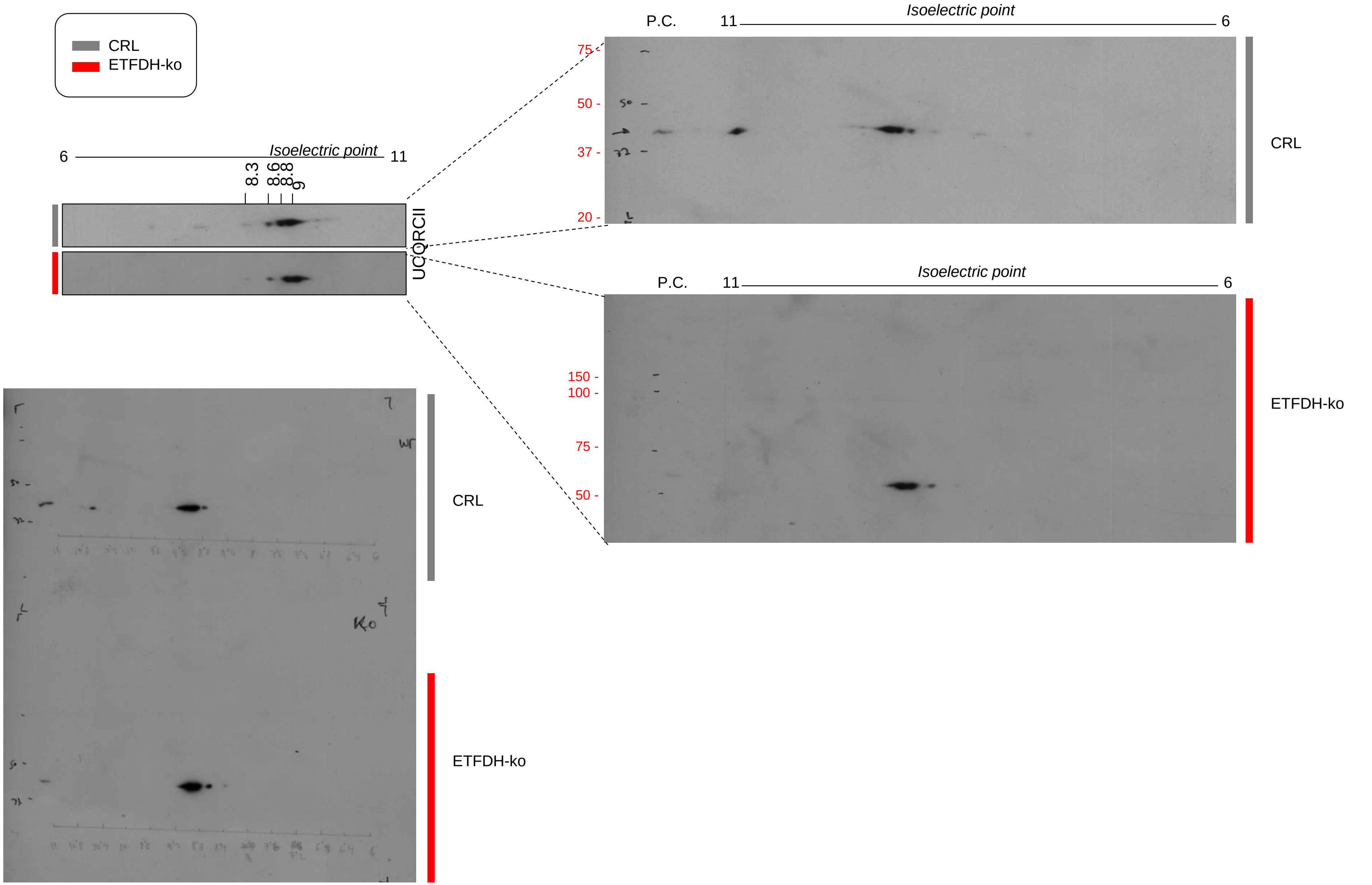

Extended data Figure 2

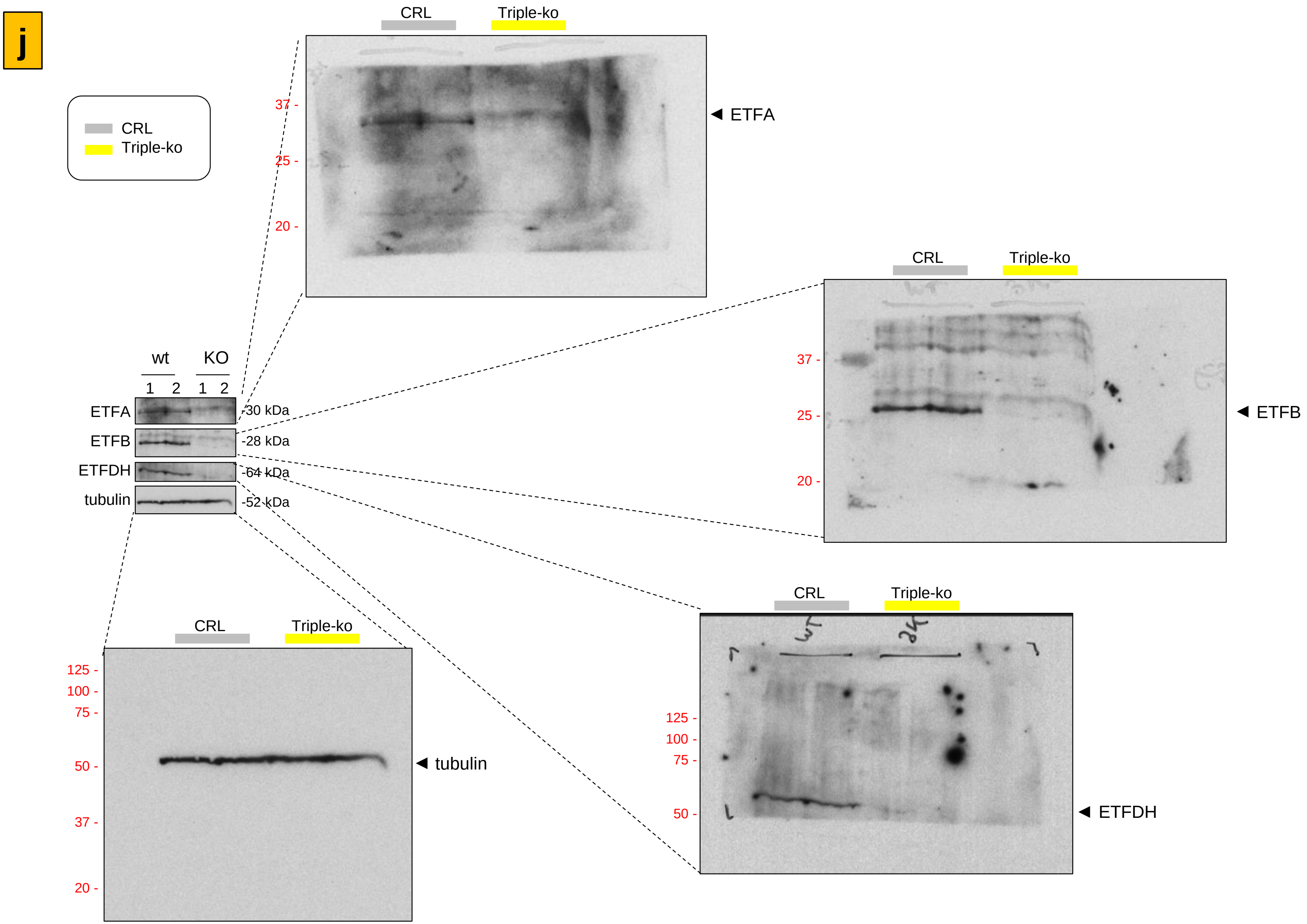

Extended data Figure 6

c

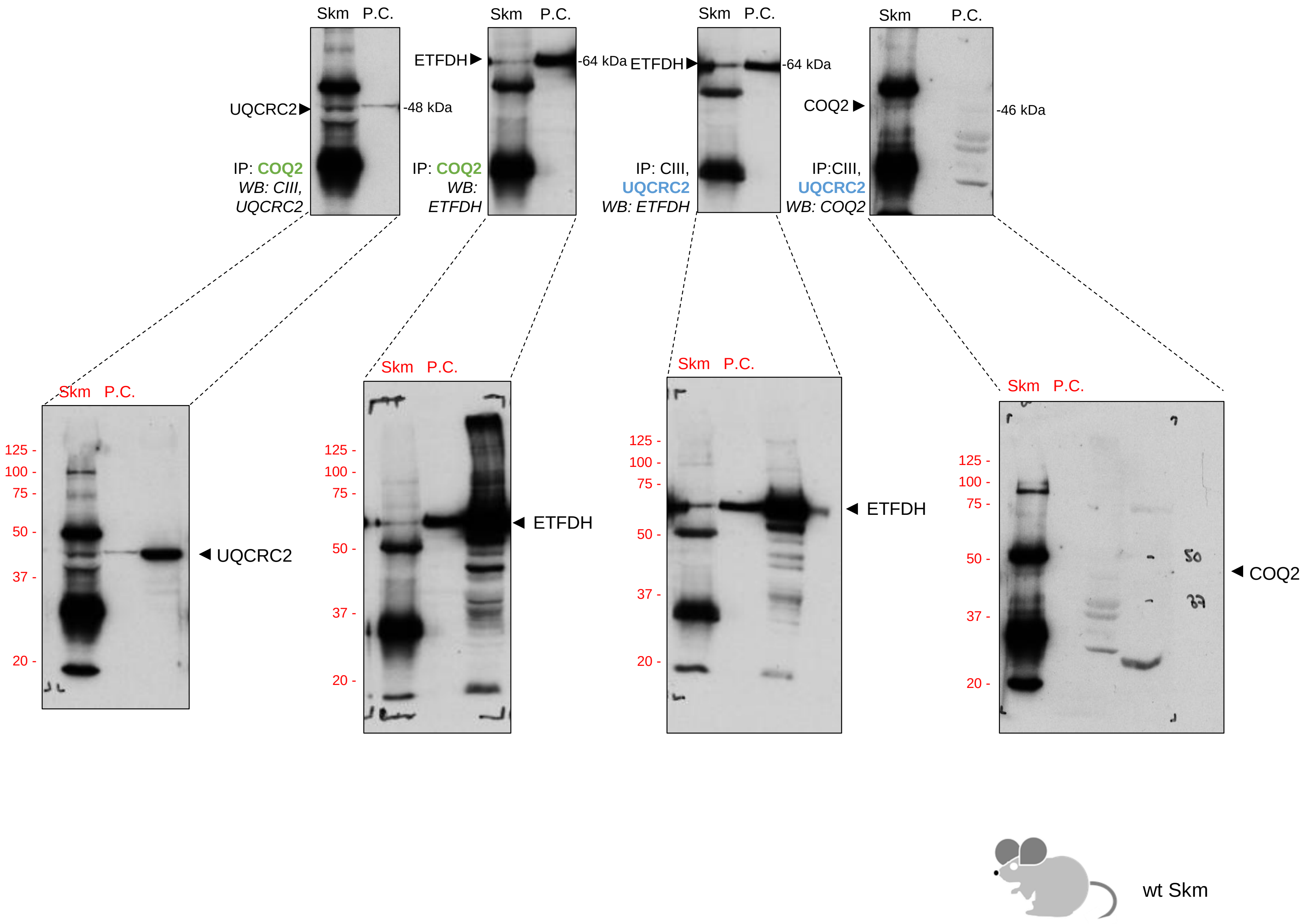

Extended data Figure 8

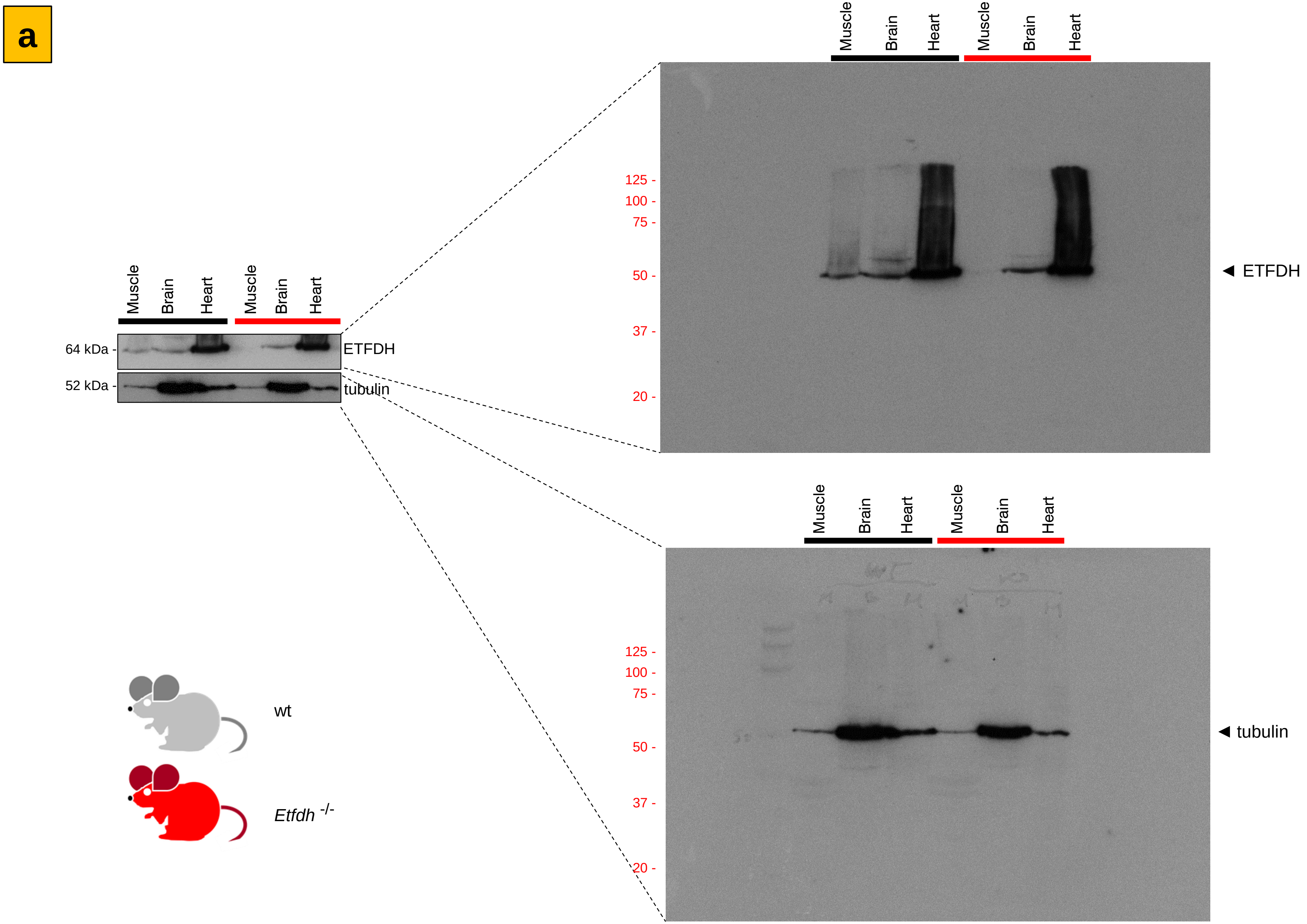

Supplement: Supplementary file 7 — Unprocessed western blots. [file 42255_2023_956_MOESM7_ESM.pdf]
